# Supplementary material for: Bone mineral density reference standards for Chinese children aged 3–18: cross-sectional results of the 2013–2015 China Child and Adolescent Cardiovascular Health (CCACH) Study
Source: BMJ Open. 2017 May 29;7(5):e014542. doi: 10.1136/bmjopen-2016-014542 (PMC5729998; doi:10.1136/bmjopen-2016-014542)
Supplement: Supplementary figure and Tables [file bmjopen-2016-014542supp001.pdf]

Supplemental Figure 1. Geographical distribution of sampled pediatric participants

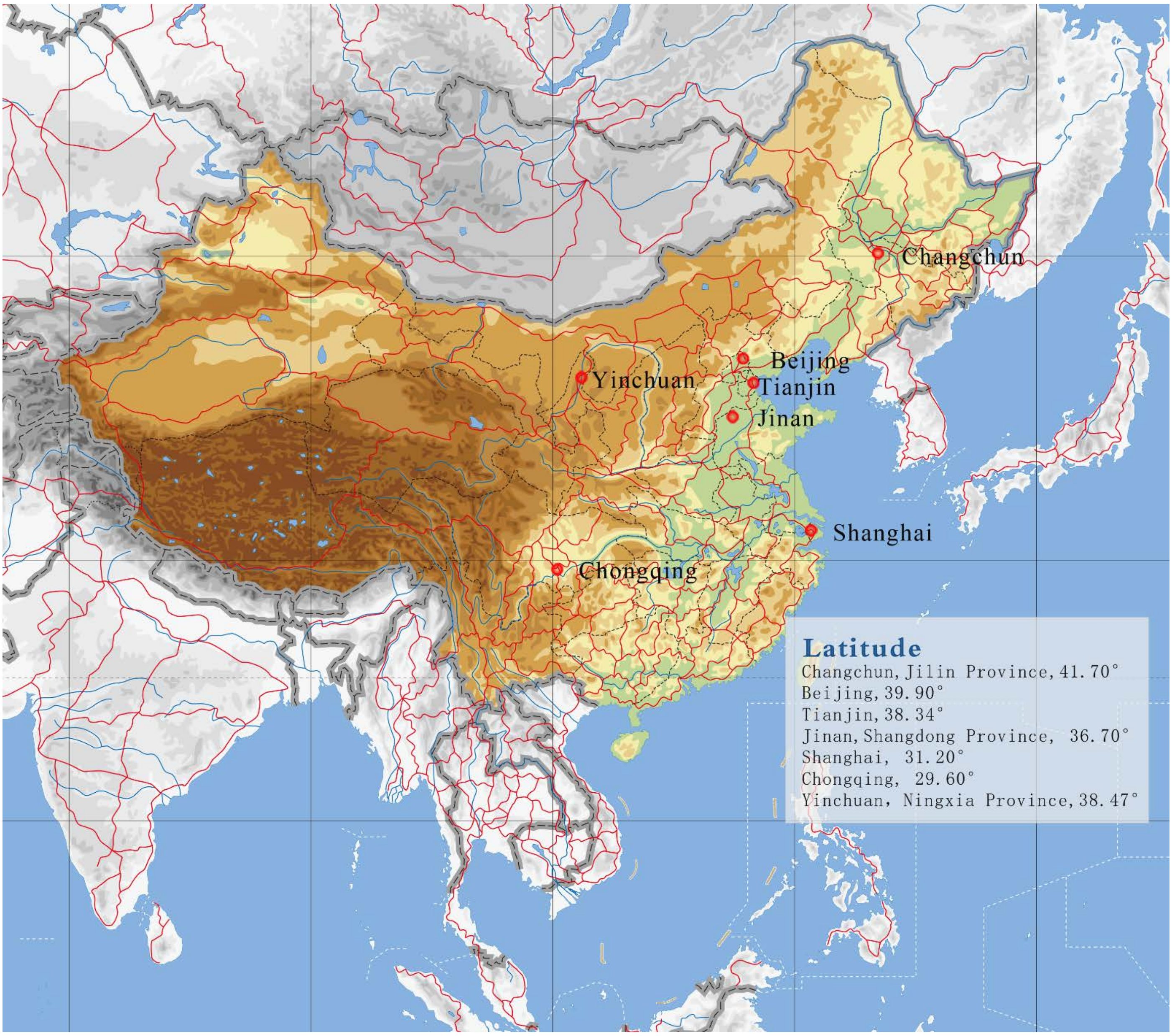

Supplemental Table 1. LMS parameters and reference centiles of TBLH BMD /g·cm^{-2} by age in boys

Age/yrs	L	S	Mean-2SD[a]	Mean-1SD[b]	Mean[c]	Mean+1SD[d]	Mean+2SD[e]	Percentiles					
								P3	P10	P25	P75	P90	P97
**3**	-0.251	0.073	0.342	0.367	0.395	0.425	0.458	0.345	0.360	0.376	0.415	0.434	0.454
**4**	-0.251	0.074	0.364	0.391	0.421	0.454	0.490	0.367	0.383	0.400	0.443	0.463	0.485
**5**	-0.251	0.076	0.399	0.429	0.463	0.500	0.541	0.402	0.420	0.440	0.487	0.511	0.536
**6**	-0.251	0.078	0.433	0.468	0.505	0.547	0.592	0.437	0.458	0.479	0.533	0.559	0.587
**7**	-0.251	0.080	0.476	0.515	0.557	0.604	0.655	0.480	0.503	0.528	0.588	0.618	0.649
**8**	-0.251	0.082	0.508	0.550	0.596	0.648	0.705	0.513	0.538	0.565	0.630	0.663	0.698
**9**	-0.251	0.084	0.532	0.577	0.627	0.682	0.743	0.537	0.564	0.593	0.663	0.699	0.736
**10**	-0.251	0.085	0.555	0.603	0.656	0.715	0.781	0.560	0.589	0.620	0.695	0.733	0.773
**11**	-0.251	0.087	0.579	0.630	0.687	0.751	0.821	0.585	0.615	0.648	0.729	0.770	0.813
**12**	-0.251	0.089	0.609	0.664	0.725	0.793	0.870	0.615	0.648	0.683	0.770	0.814	0.860
**13**	-0.251	0.091	0.645	0.704	0.771	0.845	0.928	0.652	0.687	0.725	0.820	0.867	0.918
**14**	-0.251	0.093	0.683	0.747	0.818	0.899	0.989	0.690	0.728	0.769	0.872	0.923	0.978
**15**	-0.251	0.095	0.713	0.782	0.858	0.944	1.042	0.721	0.762	0.806	0.915	0.971	1.030
**16**	-0.251	0.096	0.738	0.810	0.891	0.983	1.086	0.747	0.789	0.836	0.952	1.011	1.073
**17**	-0.251	0.098	0.765	0.841	0.927	1.024	1.134	0.774	0.819	0.868	0.991	1.054	1.120
**18**	-0.251	0.100	0.781	0.860	0.949	1.050	1.165	0.790	0.836	0.888	1.016	1.081	1.151

Abbreviations: TBLH BMD, total body less head bone mineral density; SD, standard deviation.

[a] Approximately 2.28th percent

[b] Approximately 15.87th percent

[c] Approximately 50th percent

[d] Approximately 84.13th percent

[e] Approximately 97.72th percent

Supplemental Table 2. LMS parameters and reference centiles of TBLH BMD /g·cm^{-2} by age in girls

Age/yrs	L	S	Mean-2SD[a]	Mean-1SD[b]	Mean[c]	Mean+1SD[d]	Mean+2SD[e]	Percentiles					
								P3	P10	P25	P75	P90	P97
**3**	-0.480	0.061	0.342	0.363	0.385	0.409	0.436	0.345	0.357	0.370	0.401	0.417	0.433
**4**	-0.417	0.064	0.358	0.381	0.406	0.433	0.463	0.361	0.374	0.389	0.424	0.441	0.459
**5**	-0.293	0.070	0.391	0.419	0.449	0.482	0.518	0.395	0.411	0.428	0.471	0.492	0.513
**6**	-0.166	0.076	0.423	0.456	0.491	0.531	0.573	0.427	0.446	0.467	0.517	0.542	0.568
**7**	-0.035	0.082	0.455	0.493	0.535	0.581	0.630	0.459	0.482	0.506	0.565	0.594	0.624
**8**	0.092	0.087	0.482	0.527	0.575	0.626	0.682	0.487	0.514	0.542	0.609	0.642	0.676
**9**	0.205	0.091	0.508	0.558	0.612	0.670	0.733	0.514	0.544	0.576	0.651	0.687	0.725
**10**	0.294	0.094	0.537	0.592	0.652	0.715	0.783	0.543	0.576	0.611	0.694	0.734	0.775
**11**	0.358	0.095	0.573	0.633	0.696	0.764	0.836	0.580	0.615	0.653	0.742	0.784	0.827
**12**	0.388	0.092	0.613	0.676	0.743	0.813	0.887	0.620	0.658	0.697	0.790	0.834	0.878
**13**	0.375	0.088	0.651	0.714	0.781	0.852	0.927	0.658	0.696	0.736	0.829	0.873	0.918
**14**	0.314	0.084	0.680	0.742	0.807	0.877	0.950	0.687	0.724	0.763	0.854	0.897	0.941
**15**	0.208	0.080	0.699	0.759	0.823	0.891	0.963	0.706	0.741	0.779	0.868	0.911	0.954
**16**	0.071	0.078	0.714	0.772	0.835	0.902	0.975	0.720	0.755	0.792	0.880	0.922	0.966
**17**	-0.087	0.077	0.725	0.783	0.845	0.913	0.987	0.732	0.766	0.802	0.890	0.933	0.978
**18**	-0.257	0.076	0.732	0.788	0.850	0.918	0.993	0.738	0.771	0.807	0.895	0.938	0.984

Abbreviations: TBLH BMD, total body less head bone mineral density; SD, standard deviation.

[a] Approximately 2.28th percent

[b] Approximately 15.87th percent

[c] Approximately 50th percent

[d] Approximately 84.13th percent

[e] Approximately 97.72th percent

Supplemental Table 3. LMS parameters and reference centiles of TBLH BMD /g·cm^{-2} by height/cm in boys

Height/cm	L	S	Mean-2SD[a]	Mean-1SD[b]	Mean[c]	Mean+1SD[d]	Mean+2SD[e]	Percentiles					
								P3	P10	P25	P75	P90	P97
95	-1.272	0.045	0.343	0.358	0.374	0.392	0.412	0.345	0.354	0.363	0.386	0.397	0.409
100	-1.272	0.050	0.360	0.377	0.395	0.416	0.439	0.362	0.372	0.382	0.409	0.422	0.436
105	-1.272	0.054	0.380	0.399	0.420	0.444	0.472	0.382	0.393	0.405	0.436	0.452	0.468
110	-1.272	0.059	0.403	0.424	0.449	0.477	0.510	0.405	0.418	0.432	0.468	0.486	0.506
115	-1.272	0.064	0.429	0.454	0.483	0.516	0.554	0.432	0.447	0.463	0.504	0.526	0.549
120	-1.272	0.068	0.458	0.486	0.519	0.558	0.603	0.461	0.478	0.496	0.544	0.570	0.597
125	-1.272	0.072	0.486	0.518	0.555	0.599	0.651	0.490	0.509	0.530	0.584	0.613	0.645
130	-1.272	0.076	0.512	0.547	0.588	0.637	0.696	0.516	0.537	0.560	0.620	0.652	0.688
135	-1.272	0.079	0.537	0.575	0.620	0.674	0.739	0.541	0.564	0.589	0.655	0.691	0.731
140	-1.272	0.082	0.560	0.601	0.649	0.708	0.780	0.564	0.588	0.615	0.687	0.726	0.770
145	-1.272	0.085	0.581	0.624	0.677	0.740	0.819	0.585	0.611	0.640	0.718	0.761	0.809
150	-1.272	0.087	0.604	0.651	0.708	0.776	0.862	0.609	0.637	0.668	0.752	0.798	0.851
155	-1.272	0.090	0.634	0.684	0.745	0.820	0.915	0.639	0.669	0.703	0.794	0.845	0.902
160	-1.272	0.093	0.667	0.722	0.788	0.870	0.974	0.673	0.706	0.742	0.841	0.897	0.960
165	-1.272	0.095	0.701	0.760	0.831	0.919	1.032	0.707	0.742	0.781	0.888	0.948	1.017
170	-1.272	0.097	0.733	0.795	0.871	0.965	1.087	0.739	0.776	0.818	0.932	0.997	1.071
175	-1.272	0.098	0.762	0.827	0.908	1.008	1.138	0.769	0.808	0.852	0.973	1.041	1.121
180	-1.272	0.100	0.790	0.859	0.943	1.049	1.187	0.797	0.838	0.884	1.012	1.084	1.169
185	-1.272	0.101	0.818	0.890	0.979	1.091	1.236	0.826	0.868	0.917	1.051	1.128	1.217
190	-1.272	0.102	0.847	0.922	1.015	1.133	1.286	0.855	0.899	0.950	1.091	1.171	1.265
195	-1.272	0.103	0.876	0.955	1.052	1.175	1.337	0.884	0.931	0.984	1.132	1.216	1.315

Abbreviations: TBLH BMD, total body less head bone mineral density; SD, standard deviation.

[a] Approximately 2.28th percent

[b] Approximately 15.87th percent

[c] Approximately 50th percent

[d] Approximately 84.13th percent

[e] Approximately 97.72th percent

Supplemental Table 4. LMS parameters and reference centiles of-TBLH BMD /g·cm^{-2} by height/cm in girls

**Height/cm**	**L**	**S**	**Mean-2SD[a]**	**Mean-1SD[b]**	**Mean[c]**	**Mean+1SD[d]**	**Mean+2SD[e]**	**Percentiles**					
								**P3**	**P10**	**P25**	**P75**	**P90**	**P97**
95	-0.926	0.047	0.339	0.354	0.371	0.389	0.409	0.340	0.350	0.359	0.383	0.395	0.407
100	-1.168	0.050	0.355	0.371	0.390	0.411	0.434	0.356	0.366	0.377	0.404	0.417	0.431
105	-1.413	0.054	0.376	0.395	0.416	0.439	0.467	0.378	0.389	0.401	0.431	0.447	0.463
110	-1.665	0.058	0.398	0.419	0.443	0.471	0.504	0.401	0.413	0.427	0.461	0.480	0.500
115	-1.871	0.064	0.423	0.446	0.474	0.508	0.550	0.425	0.439	0.455	0.496	0.519	0.544
120	-1.980	0.072	0.449	0.476	0.509	0.550	0.603	0.452	0.468	0.486	0.536	0.563	0.596
125	-2.008	0.078	0.477	0.508	0.546	0.595	0.659	0.480	0.499	0.520	0.578	0.611	0.650
130	-1.950	0.083	0.501	0.536	0.579	0.634	0.708	0.505	0.526	0.549	0.614	0.653	0.698
135	-1.776	0.088	0.521	0.560	0.607	0.668	0.750	0.525	0.548	0.574	0.647	0.689	0.739
140	-1.499	0.093	0.545	0.589	0.642	0.710	0.798	0.550	0.576	0.605	0.686	0.732	0.786
145	-1.146	0.096	0.580	0.630	0.690	0.763	0.856	0.585	0.615	0.648	0.737	0.787	0.844
150	-0.764	0.095	0.626	0.682	0.747	0.824	0.916	0.632	0.665	0.702	0.798	0.848	0.905
155	-0.417	0.089	0.668	0.727	0.793	0.869	0.954	0.674	0.709	0.747	0.843	0.892	0.944
160	-0.159	0.083	0.698	0.756	0.821	0.892	0.970	0.704	0.739	0.777	0.868	0.913	0.961
165	-0.004	0.078	0.721	0.779	0.843	0.912	0.986	0.727	0.762	0.800	0.889	0.932	0.977
170	0.096	0.077	0.741	0.800	0.864	0.933	1.007	0.747	0.783	0.821	0.910	0.953	0.998
175	0.187	0.076	0.760	0.822	0.887	0.956	1.030	0.767	0.804	0.842	0.933	0.976	1.021
180	0.281	0.075	0.781	0.844	0.910	0.980	1.054	0.788	0.826	0.865	0.957	1.000	1.045

Abbreviations: TBLH BMD, total body less head bone mineral density; SD, standard deviation.

[a] Approximately 2.28th percent

[b] Approximately 15.87th percent

[c] Approximately 50th percent

[d] Approximately 84.13th percent

[e] Approximately 97.72th percent

Supplemental Table 5. LMS parameters and reference centiles of FMP by age in boys

**Age/yrs**	**L**	**S**	**Mean-2SD**[a]	**Mean-1SD**[b]	**Mean**[c]	**Mean+1SD**[d]	**Mean+2SD**[e]	**Percentiles**					
								**P3**	**P10**	**P25**	**P75**	**P90**	**P97**
**3**	-0.651	0.101	26.1	28.6	31.5	35.0	39.1	26.3	27.8	29.5	33.8	36.1	38.6
**4**	-0.585	0.120	24.4	27.2	30.6	34.7	39.7	24.7	26.4	28.3	33.3	36.0	39.0
**5**	-0.483	0.151	22.2	25.4	29.4	34.3	40.7	22.5	24.4	26.6	32.6	36.0	39.8
**6**	-0.388	0.179	20.5	24.1	28.7	34.5	42.1	20.9	23.0	25.5	32.5	36.5	41.1
**7**	-0.301	0.204	19.4	23.3	28.4	35.1	43.9	19.8	22.1	24.9	32.7	37.3	42.7
**8**	-0.230	0.224	18.6	22.9	28.5	35.9	45.7	19.1	21.6	24.6	33.2	38.4	44.4
**9**	-0.184	0.240	18.1	22.7	28.7	36.6	47.4	18.6	21.2	24.4	33.8	39.4	45.9
**10**	-0.163	0.252	17.6	22.3	28.6	37.0	48.4	18.1	20.9	24.2	34.0	39.9	46.9
**11**	-0.168	0.261	17.0	21.7	28.0	36.6	48.4	17.5	20.3	23.6	33.5	39.6	46.8
**12**	-0.198	0.267	16.3	20.8	27.0	35.5	47.4	16.7	19.4	22.6	32.4	38.4	45.8
**13**	-0.250	0.269	15.5	19.8	25.6	33.9	45.7	15.9	18.4	21.5	30.9	36.8	44.1
**14**	-0.318	0.270	14.8	18.8	24.3	32.2	43.9	15.2	17.5	20.4	29.3	35.1	42.3
**15**	-0.397	0.269	14.3	18.0	23.2	30.9	42.5	14.7	16.8	19.5	28.1	33.7	40.9
**16**	-0.481	0.266	14.0	17.5	22.5	29.9	41.6	14.3	16.4	18.9	27.1	32.6	39.9
**17**	-0.564	0.264	13.8	17.1	21.9	29.1	40.9	14.1	16.1	18.5	26.4	31.8	39.2
**18**	-0.645	0.261	13.6	16.7	21.3	28.3	40.3	13.9	15.7	18.0	25.7	31.1	38.5

Abbreviations: FMP, fat mass percentage (%); SD, standard deviation.

[a] Approximately 2.28th percent

[b] Approximately 15.87th percent

[c] Approximately 50th percent

[d] Approximately 84.13th percent

[e] Approximately 97.72th percent

Supplemental Table 6. LMS parameters and reference centiles of FMP by age in girls

Age/yrs	L	S	Mean-2SD[a]	Mean-1SD[b]	Mean[c]	Mean+1SD[d]	Mean+2SD[e]	Percentiles					
								P3	P10	P25	P75	P90	P97
**3**	0.285	0.106	27.8	31.1	34.7	38.5	42.6	28.2	30.2	32.2	37.2	39.6	42.1
**4**	0.285	0.118	26.6	30.1	34.0	38.1	42.7	27.0	29.1	31.3	36.7	39.4	42.1
**5**	0.285	0.140	24.5	28.4	32.8	37.6	42.9	24.9	27.3	29.8	36.0	39.0	42.2
**6**	0.285	0.158	22.8	27.1	31.8	37.2	43.1	23.3	25.8	28.6	35.4	38.8	42.4
**7**	0.285	0.173	21.6	26.1	31.1	36.9	43.3	22.1	24.8	27.7	34.9	38.6	42.5
**8**	0.285	0.183	20.9	25.4	30.7	36.7	43.4	21.4	24.1	27.1	34.6	38.5	42.6
**9**	0.285	0.187	20.5	25.1	30.5	36.6	43.5	21.0	23.8	26.8	34.5	38.4	42.6
**10**	0.285	0.188	20.4	25.0	30.4	36.5	43.4	20.9	23.7	26.7	34.4	38.3	42.5
**11**	0.285	0.185	20.6	25.1	30.4	36.4	43.2	21.1	23.8	26.8	34.4	38.2	42.3
**12**	0.285	0.179	21.0	25.5	30.6	36.5	43.1	21.5	24.1	27.1	34.5	38.3	42.3
**13**	0.285	0.173	21.6	26.0	31.1	36.8	43.2	22.1	24.7	27.6	34.8	38.5	42.4
**14**	0.285	0.165	22.4	26.8	31.7	37.3	43.5	22.9	25.5	28.3	35.4	38.9	42.7
**15**	0.285	0.157	23.4	27.6	32.5	37.9	43.9	23.8	26.4	29.1	36.0	39.5	43.1
**16**	0.285	0.150	24.2	28.4	33.1	38.4	44.2	24.7	27.2	29.9	36.6	39.9	43.5
**17**	0.285	0.143	24.9	29.0	33.5	38.6	44.2	25.3	27.8	30.4	36.9	40.1	43.5
**18**	0.285	0.137	25.3	29.3	33.7	38.5	43.9	25.8	28.1	30.7	36.9	40.0	43.2

Abbreviations: FMP, fat mass percentage (%); SD, standard deviation.

[a] Approximately 2.28th percent

[b] Approximately 15.87th percent

[c] Approximately 50th percent

[d] Approximately 84.13th percent

[e] Approximately 97.72th percent

Supplemental Table 7. LMS parameters and reference centiles of FMI by age in boys

Age/yrs	L	S	Mean-2SD[a]	Mean-1SD[b]	Mean[c]	Mean+1SD[d]	Mean+2SD[e]	Percentiles					
								P3	P10	P25	P75	P90	P97
**3**	-0.682	0.133	3.93	4.41	5.01	5.76	6.72	3.98	4.27	4.59	5.50	6.00	6.59
**4**	-0.667	0.167	3.57	4.12	4.83	5.77	7.05	3.63	3.95	4.33	5.43	6.09	6.88
**5**	-0.645	0.220	3.13	3.75	4.61	5.84	7.72	3.19	3.56	4.00	5.38	6.29	7.45
**6**	-0.625	0.268	2.85	3.54	4.53	6.08	8.71	2.92	3.32	3.82	5.49	6.68	8.31
**7**	-0.608	0.311	2.70	3.44	4.57	6.45	9.99	2.77	3.20	3.75	5.72	7.22	9.43
**8**	-0.593	0.347	2.63	3.43	4.70	6.92	11.46	2.71	3.17	3.77	6.04	7.86	10.70
**9**	-0.578	0.373	2.62	3.47	4.86	7.40	12.91	2.70	3.19	3.84	6.38	8.51	11.97
**10**	-0.565	0.392	2.61	3.51	5.00	7.79	14.07	2.70	3.21	3.91	6.66	9.03	12.97
**11**	-0.553	0.404	2.59	3.51	5.05	7.97	14.70	2.68	3.20	3.92	6.78	9.29	13.52
**12**	-0.541	0.410	2.55	3.46	5.02	7.97	14.80	2.63	3.16	3.88	6.77	9.30	13.60
**13**	-0.530	0.412	2.49	3.40	4.93	7.84	14.52	2.58	3.09	3.80	6.65	9.14	13.34
**14**	-0.520	0.410	2.44	3.32	4.82	7.65	14.04	2.52	3.03	3.72	6.50	8.91	12.93
**15**	-0.510	0.407	2.40	3.27	4.73	7.47	13.53	2.48	2.98	3.66	6.36	8.68	12.48
**16**	-0.501	0.402	2.37	3.23	4.66	7.31	13.05	2.45	2.95	3.61	6.24	8.47	12.07
**17**	-0.492	0.398	2.35	3.20	4.60	7.16	12.61	2.43	2.92	3.58	6.13	8.27	11.69
**18**	-0.483	0.393	2.33	3.16	4.54	7.01	12.18	2.41	2.89	3.53	6.02	8.08	11.32

Abbreviations: FMI, fat mass index (Fat mass (kg)/height(m)^2) ; SD, standard deviation.

[a] Approximately 2.28th percent

[b] Approximately 15.87th percent

[c] Approximately 50th percent

[d] Approximately 84.13th percent

[e] Approximately 97.72th percent

Supplemental Table 8. LMS parameters for the reference centiles of FMI by age in girls

Age/yrs	L	S	Mean-2SD[a]	Mean-1SD[b]	Mean[c]	Mean+1SD[d]	Mean+2SD[e]	Percentiles					
								P3	P10	P25	P75	P90	P97
**3**	-0.731	0.146	4.11	4.66	5.36	6.25	7.45	4.17	4.49	4.87	5.94	6.55	7.28
**4**	-0.697	0.167	3.87	4.46	5.23	6.25	7.65	3.93	4.28	4.69	5.88	6.60	7.46
**5**	-0.635	0.206	3.47	4.13	5.01	6.25	8.08	3.54	3.92	4.38	5.79	6.69	7.82
**6**	-0.577	0.239	3.19	3.89	4.86	6.29	8.51	3.26	3.67	4.17	5.76	6.81	8.19
**7**	-0.523	0.266	3.00	3.74	4.79	6.38	8.94	3.07	3.50	4.04	5.79	6.98	8.57
**8**	-0.472	0.287	2.90	3.69	4.82	6.56	9.41	2.98	3.44	4.01	5.91	7.22	8.98
**9**	-0.423	0.300	2.90	3.73	4.95	6.82	9.89	2.98	3.47	4.08	6.12	7.53	9.44
**10**	-0.376	0.308	2.96	3.84	5.14	7.12	10.34	3.04	3.56	4.21	6.38	7.87	9.87
**11**	-0.331	0.309	3.06	4.00	5.38	7.45	10.74	3.16	3.70	4.39	6.67	8.22	10.26
**12**	-0.288	0.306	3.21	4.21	5.65	7.79	11.09	3.32	3.90	4.62	6.99	8.57	10.61
**13**	-0.246	0.299	3.40	4.46	5.95	8.11	11.35	3.51	4.12	4.88	7.31	8.89	10.89
**14**	-0.205	0.288	3.62	4.72	6.25	8.41	11.54	3.73	4.37	5.16	7.62	9.18	11.10
**15**	-0.165	0.276	3.85	4.99	6.53	8.66	11.64	3.97	4.63	5.44	7.89	9.40	11.23
**16**	-0.126	0.263	4.06	5.22	6.76	8.83	11.64	4.18	4.86	5.67	8.08	9.53	11.26
**17**	-0.088	0.250	4.24	5.40	6.91	8.90	11.52	4.36	5.04	5.84	8.19	9.56	11.17
**18**	-0.051	0.237	4.38	5.53	7.01	8.90	11.33	4.50	5.18	5.97	8.23	9.52	11.01

Abbreviations: FMI, fat mass index (Fat mass (kg)/height(m)^2) ; SD, standard deviation.

[a] Approximately 2.28th percent

[b] Approximately 15.87th percent

[c] Approximately 50th percent

[d] Approximately 84.13th percent

[e] Approximately 97.72th percent

Supplemental Table 9. LMS parameters and reference centiles of FFMI by age in boys

Age/yrs	L	S	Mean-2SD[a]	Mean-1SD[b]	Mean[c]	Mean+1SD[d]	Mean+2SD[e]	Percentiles					
								P3	P10	P25	P75	P90	P97
**3**	-0.656	0.058	9.58	10.12	10.71	11.36	12.07	9.64	9.96	10.30	11.14	11.55	11.99
**4**	-0.656	0.062	9.66	10.25	10.90	11.61	12.41	9.73	10.08	10.45	11.37	11.83	12.31
**5**	-0.656	0.070	9.80	10.46	11.20	12.04	12.98	9.87	10.27	10.69	11.76	12.29	12.87
**6**	-0.656	0.078	9.86	10.60	11.43	12.38	13.47	9.94	10.38	10.86	12.06	12.68	13.34
**7**	-0.656	0.085	9.92	10.73	11.66	12.73	13.98	10.01	10.49	11.02	12.37	13.06	13.82
**8**	-0.656	0.093	10.08	10.98	12.01	13.22	14.64	10.18	10.71	11.30	12.81	13.60	14.46
**9**	-0.656	0.100	10.22	11.20	12.34	13.70	15.31	10.33	10.91	11.55	13.23	14.12	15.10
**10**	-0.656	0.108	10.35	11.41	12.66	14.15	15.96	10.47	11.09	11.79	13.63	14.62	15.73
**11**	-0.656	0.114	10.61	11.75	13.12	14.76	16.78	10.74	11.41	12.17	14.19	15.29	16.52
**12**	-0.656	0.118	11.06	12.29	13.78	15.59	17.82	11.19	11.92	12.75	14.96	16.17	17.53
**13**	-0.656	0.121	11.66	12.99	14.60	16.56	19.00	11.81	12.59	13.48	15.88	17.19	18.68
**14**	-0.656	0.122	12.30	13.72	15.42	17.51	20.11	12.46	13.29	14.24	16.78	18.18	19.77
**15**	-0.656	0.121	12.78	14.24	16.00	18.16	20.84	12.94	13.80	14.78	17.41	18.85	20.49
**16**	-0.656	0.120	13.02	14.50	16.27	18.43	21.12	13.19	14.06	15.04	17.68	19.13	20.77
**17**	-0.656	0.118	13.19	14.66	16.43	18.57	21.22	13.35	14.22	15.20	17.83	19.26	20.88
**18**	-0.656	0.116	13.36	14.82	16.56	18.68	21.29	13.52	14.38	15.35	17.95	19.36	20.95

Abbreviations: FFMI, free fat mass index (Free fat mass (kg)/height(m)^2) ; SD, standard deviation.

[a] Approximately 2.28th percent

[b] Approximately 15.87th percent

[c] Approximately 50th percent

[d] Approximately 84.13th percent

[e] Approximately 97.72th percent

Supplemental Table 10. LMS parameters and reference centiles of FFMI by age in girls

Age/yrs	L	S	Mean-2SD[a]	Mean-1SD[b]	Mean[c]	Mean+1SD[d]	Mean+2SD[e]	Percentiles					
								P3	P10	P25	P75	P90	P97
**3**	-0.485	0.060	8.97	9.50	10.09	10.72	11.42	9.03	9.35	9.69	10.51	10.91	11.33
**4**	-0.484	0.064	9.00	9.57	10.19	10.87	11.62	9.06	9.40	9.76	10.64	11.07	11.53
**5**	-0.482	0.071	9.06	9.69	10.39	11.16	12.03	9.13	9.50	9.91	10.90	11.40	11.92
**6**	-0.480	0.078	9.11	9.81	10.59	11.46	12.44	9.19	9.60	10.05	11.16	11.72	12.32
**7**	-0.479	0.084	9.19	9.96	10.81	11.79	12.90	9.28	9.73	10.22	11.46	12.09	12.76
**8**	-0.477	0.091	9.34	10.17	11.11	12.19	13.43	9.43	9.92	10.46	11.82	12.52	13.28
**9**	-0.475	0.096	9.55	10.46	11.49	12.68	14.06	9.66	10.19	10.78	12.27	13.04	13.88
**10**	-0.474	0.101	9.84	10.81	11.93	13.22	14.74	9.94	10.52	11.15	12.78	13.63	14.55
**11**	-0.472	0.104	10.17	11.21	12.40	13.80	15.44	10.29	10.90	11.58	13.32	14.24	15.23
**12**	-0.470	0.106	10.51	11.60	12.87	14.34	16.08	10.63	11.28	11.99	13.84	14.81	15.86
**13**	-0.469	0.106	10.81	11.94	13.24	14.77	16.57	10.94	11.60	12.34	14.25	15.25	16.34
**14**	-0.467	0.106	11.05	12.19	13.51	15.06	16.88	11.17	11.85	12.60	14.53	15.54	16.65
**15**	-0.465	0.104	11.21	12.35	13.67	15.21	17.01	11.34	12.01	12.76	14.68	15.69	16.78
**16**	-0.463	0.102	11.31	12.44	13.74	15.25	17.01	11.44	12.11	12.84	14.73	15.72	16.79
**17**	-0.462	0.099	11.39	12.50	13.77	15.24	16.94	11.52	12.17	12.89	14.73	15.69	16.73
**18**	-0.460	0.096	11.48	12.56	13.80	15.22	16.87	11.60	12.24	12.94	14.74	15.66	16.66

Abbreviations: FFMI, free fat mass index (Free fat mass (kg)/height(m)^2) ; SD, standard deviation.

[a] Approximately 2.28th percent

[b] Approximately 15.87th percent

[c] Approximately 50th percent

[d] Approximately 84.13th percent

[e] Approximately 97.72th percent
