# Supplementary material for: Bone mineral density reference standards for Chinese children aged 3–18: cross-sectional results of the 2013–2015 China Child and Adolescent Cardiovascular Health (CCACH) Study
Source: BMJ Open. 2017 May 29;7(5):e014542. doi: 10.1136/bmjopen-2016-014542 (PMC5729998; doi:10.1136/bmjopen-2016-014542)
Supplement: Author's manuscript [file bmjopen-2016-014542.draft_revisions.pdf]

BMJ Open

Body mineral density interpretation and reporting among Chinese children aged 3-18: The China Child and Adolescent Cardiovascular Health (CCACH)

Journal:	*BMJ Open*
Manuscript ID	bmjopen-2016-014542
Article Type:	Research
Date Submitted by the Author:	06-Oct-2016

Complete List of Authors:	Liu, Junting; Capital Institute of Pediatrics, Department of Epidemiology<br>Wang, Liang; East Tennessee State University, ; East Tennessee State University<br>Sun, Jinghui ; The First Hospital of Jilin University<br>Liu, Gongshu; Tianjin Women's and Children's Health Center,<br>Yan, Weili; Fudan Univ,<br>Xi, Bo; Shandong University<br>Xiong, Feng; Children's Hospital of Chongqing Medical University, Department of Endocrinology<br>Ding, Wenqing; Ningxia Medical University, Department of Public Health<br>Huang, Guimin; Capital Institute of Pediatrics, Department of Epidemiology<br>Heymsfield, Steven; Pennington Biomedical Research Center, Metabolism - Body Composition<br>Mi, Jie; Capital Institute of Pediatrics, Department of Epidemiology

**Primary Subject Heading**:	Epidemiology
Secondary Subject Heading:	Paediatrics, Public health
Keywords:	BMD, DXA, Children, China

SCHOLARONE™ Manuscripts

Body mineral density interpretation and reporting among Chinese children aged 3-18: The China Child and Adolescent Cardiovascular Health (CCACH)

Junting Liu[1#], MS, Liang Wang[2#],MD, DrPH, MPH, Jinghui Sun, MD[3†],Gongshu Liu, MD [4†] ,Weili Yan, PhD[5†],Bo Xi[6†], PhD, Feng Xiong[7†], MS, Wenqing Ding[8†],PhD, Guimin Huang[1], MS, Steven B. Heymsfield, PhD,[9] Jie Mi[1*],MD,PhD[1]

[# †]These authors contributed equally to the work.

[1]Department of Epidemiology, Capital Institute of Pediatrics, Beijing 100020, China
[2]Department of Biostatistics and Epidemiology, College of Public Health, East Tennessee State University, TN 37614, USA
[3]Department of Cardiovascular Medicine, The First Hospital of Jilin University, Changchun 130021, China
[4]Project Office, Tianjin Women's and Children's Health Center, Tianjin 300070, China
[5]Department of Clinical Epidemiology, Children's Hospital of Fudan University, Shanghai 200433, China
[6]Department of Public Health, Shandong University, Jinan 250012, China
[7]Department of Endocrinology, Children's Hospital of Chongqing Medical University, Chongqing 400014, China
[8]Department of Public Health, Ningxia Medical University, Yinchuan 750003, China
[9]Department of Metabolism-Body Composition, Pennington Biomedical Research Center, Baton Rouge, LA 70808, USA

Email:
Junting Liu, Junting_liu@163.com; Liang Wang, wangl2@etsu.edu; Jinghui Sun, 13756661617@126.com; Gongshu Liu, Liugongshu727@163.com; Weili Yan, yanwl@fudan.edu.cn; Bo Xi, swei006@163.com; Feng Xiong, xiongfw2013@163.com; Wenqing Ding,13519598269@163.com; Guimin Huang, guiminhuang@163.com; Steven B. Heymsfield, steven.heymsfield@pbrc.edu; Jie Mi, Jiemi@vip.163.com

Corresponding author
Jie Mi, Department of Epidemiology, Capital Institute of Pediatrics, 2 Yabao Road, Beijing 100020, China. Tel: +86-10-85695591; Fax: +86-10-85632799; jiemi@vip.163.com.

Word count for main text: 2703

Number of table: 3

Number of figure: 1

Number of supplemental table: 2

Number of supplemental figure: 1

For peer review only

For peer review only - http://bmjopen.bmj.com/site/about/guidelines.xhtml

Abstract

Objectives: No nationwide pediatric reference data for bone mineral density (BMD) are available in China. We aimed to provide sex-specific BMD reference values for Chinese children and adolescents (3-18 years).

Methods: Data (10,818 participants aged 3-18 years) were obtained from cross-sectional surveys of the China Child and Adolescent Cardiovascular Health (CCACH) in 2015, including four municipality cities and three provinces. BMD was measured using Hologic Discovery Dual Energy X-ray Absorptiometry (DXA) scanner. The DXA measures were modeled against age, with height as an independent variable. The LMS statistical method using a curve fitting procedure was used to construct reference smooth cross sectional centile curves for dependent versus independent variables.

Results: Children residing in Northeast China had the highest total body less head (TBLH) BMD while children residing in Shandong Province had the lowest values. Among children, TBLH BMD was higher for boys as compared to girls; but it increased with age and height in both sexes. Furthermore, TBLH BMD was higher among U.S. children as compared to Chinese children. There was a large difference in BMD for height among children from the two countries. U.S. children had a much higher BMD at each percentile (P) than Chinese children, with the difference being the largest at P50 and P3 and being the smallest at P97.

Conclusions: This is the first study to present a sex-specific reference dataset for 3-18 years old Chinese children. The data can help clinicians improve interpretation, assessment, and monitoring of densitometry results.

Key words: BMD, DXA, China, Children

For peer review only - http://bmjopen.bmj.com/site/about/guidelines.xhtml

Strengths and limitations of this study

- It is the first study to present a sex-specific reference dataset for 3-18 years old Chinese children.
- A number of reference curves were developed using objective measures from DXA.
- The large sample size enabled us to construct stable reference curves and extended previous studies though covering a wide-range age from 3 to 18.
- The data we collected is not nationally representative and hence we could not generalize the results to the entire Chinese pediatric population.
- Our developed reference curve could not be used alone as clinical criteria for the diagnosis of osteoporosis.

For peer review only

For peer review only - http://bmjopen.bmj.com/site/about/guidelines.xhtml

Introduction

Osteoporosis, as a widespread metabolic bone disease, has become a global public health problem. More than 200 million people worldwide and 10 million people in the United States (U.S.) were afflicted with osteoporosis in 2013[1]. In China, the population with osteoporosis in 1997 was 83.9 million, which is projected to increase to 212 million by 2050[2]. Furthermore, the prevalence of osteoporosis in older Chinese adults was around 15.7%[3].

The burdens associated with osteoporosis are heavy, with the most common being the fractures. In 2005, the acute and the long-term medical care expenses associated with fractures costed the U.S. approximately 17 billion dollars. The cumulative cost over the next twenty years is predicted to be about $474 billion. Other burdens include losing work time or inability to perform daily living activities due to pain and disability associated with osteoporosis-related fractures[4]. It has been estimated that the treatment cost for hip fracture, in China, would be more than 85 billion Chinese Yuan (i.e., about 13 billion U.S. dollars) in 2020, and 1800 billion Chinese Yuan (i.e., about 271 U.S. dollars) in 2050 [5].

Peak bone mass is an important determinant for osteoporotic fracture risk. A higher peak bone mass is associated with lower risk of osteoporotic fractures later in life[6]. In general, individuals have peak bone mass in their early 20s; however, it may be occur earlier in women[7,8]. Thus, investigating the pattern of bone mineralization in children is of great interest. Dual-energy X-ray absorptiometry (DXA) has been recommended by the International Society for Clinical Densitometry (ISCD)[9] as a current criterion for measuring body mineral content (BMC) and bone mineral density (BMD). This measurement method has high precision, accuracy, speed, low radiation exposure, ease of use, and strong correlation with established methods[10-16]. To date, DXA is the most widely used bone densitometry technique in children[9].

For peer review only - http://bmjopen.bmj.com/site/about/guidelines.xhtml

For children, DXA can be used to measure whole body bone area (cm^2) and whole body BMC (g), leading to BMD, i.e., BMC divided by bone area (g/cm^2) which indicates the pediatric bone status. The use and correct identification of indicators for BMD is particularly important; which requires appropriate reference data that are matched for multiple factors including sex [17-19], chronological age, height, weight, pubertal development and ethnicity[17,20,21]. However, to date, no pediatric reference data for BMD are available in China. Furthermore, utilization of U.S. measures based on reference values derived from the U.S. National Health and Nutrition Examination Survey (NHANES) data might not be applicable for Chinese pediatric population. Therefore, it is critically important and urgent to develop reference values of BMD for Chinese children based upon data gathered from Chinese children. The reference data can help clinicians have a better estimation of the percentile on which Chinese children's DXA-measured bone parameter falls in reference to their age or height. The aim of this study is two-folds. First, develop sex-specific reference percentile curves for Chinese children based on a large, nationwide sample. Second, compare our findings with those of the study conducted by Kelly and colleagues [22] using U.S. NHANES data.

Methods

Participants

The American Heart Association defined a new concept of "ideal cardiovascular health (CVH)" for American adults and children in 2010[23], which included four health behaviors (nonsmoking, physically active, normal BMI, healthy diet) and three health factors (normal blood pressure, total cholesterol, fasting glucose). Based on these seven health behaviors and factors, the construct of CVH comprehensively combined the traditional risk factors proposed by the Framingham Heart Study, as well as life style behaviors, emphasizing the importance of

For peer review only - http://bmjopen.bmj.com/site/about/guidelines.xhtml

overall strategies in achieving optimal CV health. Our data were obtained from the China Child and Adolescent Cardiovascular Health Study (CCACHs). It is a large and nationwide ongoing study designed to examine CVH, BMD, body composition, and daily sodium intake among Chinese children ages 3-18. Stratified cluster random sampling method was used to extract 100 children per age group in each cluster (50 boys and 50 girls). Due to a majority of children are Han, accounting for 90% of the total Chinese pediatric population, CCACHs mainly collected CVH data among Han children. Children were recruited from seven provinces (centers) covering geographical areas of North, East, Northeast, Northwest, and Southwest of China, across latitude 29.6° north to latitude 41.7° north (Supplement Figure 1). The exclusion criteria were: (1) inability to give informed consent, (2) any condition, or use of any drug known to affect bone health, (3) non-removable objects (e.g., prostheses, implants, or casts), (4) body weight greater than 204 kg, height greater than 197.5 cm, or weight or height beyond the range of measure of machine, (5) participation in research involving ionizing radiation in the past year, (6) no history of fracture, (7) some chronic diseases, and (8) pregnancy. Data was collected on 11,057 children, out of which 109 were excluded due to kidney or thyroid disease and 130 were excluded because of implausible values of weight (light or heavy, BMI ≥ 3 standard deviations). Consequently, 10,818 children (5,509 males and 5,309 females) were used for the final analysis.

The study was approved by the Research Ethics Committee of Capital Institute of Pediatrics of China. The written informed consent documents for children were obtained from their parents or guardians.

DXA acquisition and quality control

The whole-body scans were performed using Hologic Discovery (A, W, and Wi) fan-beam densitometers (Hologic, Inc., Bedford, Massachusetts, USA). Following recommendations

For peer review only - http://bmjopen.bmj.com/site/about/guidelines.xhtml

of the ISCD, a high level of quality control was maintained throughout the DXA data collection and scan analysis. The specific procedures were as follows: (1) Standard phantom was checked before scanning the participants and calibrating DXA machines every morning; (2) an operator of each study location was trained and administered by the same technician certified by ISCD. The training materials included the ISCD official technician hands-on training materials and manufacturer's handbook including testing procedures and operation methods; (3) at each center, lumbar spine and hip joints of 15 participants were scanned for 3 times for the calculation. After each scan, they had to leave the scanner to repose for the next scan. All our operator staff passed the precision quality control required by ISCD, which controlled the coefficient of variation (CV %) to less than 1.9% for lumbar spine scans, 1.8% for hip joint scans, and 2.5% for femoral neck scans (Table 1); and (4) all participants were requested to remove outer garments and objects that would potentially interfere with testing. All DXA values were analyzed using Hologic Apex software version 4.0 following the manufacturer's guidelines.

Statistical analysis

The DXA measures for our pediatric participants were modeled against age or height as the independent variable. The LMS statistical method (LMS ChartMaker Pro Version 2.54)[24,25] using a curve fitting procedure was employed to construct reference smooth cross sectional centile curves for BMD/body composition versus age or height. The LMS technique estimates three parameters: median (M), coefficient of variation (S), and power in the Box-Cox transformation (L). This summarizes the distribution of the dependent variable given the covariate by L depending on the skewness of the distribution, M and S giving smooth curves for the dependence of L, M, and S on the covariate. These three parameters vary as a function of independent variables fully describing the reference data. Once these parameters are estimated,

For peer review only - http://bmjopen.bmj.com/site/about/guidelines.xhtml

then centile curves can be constructed using the formula that has been described elsewhere[10,22]. To avoid overfitting of the curves, parsimonious models were used instead of complex models when they had similar goodness of fit. After fitting the curves, the distribution of z-scores was observed and outlier analysis was performed to verify that all values were within physiological range (e.g., observations ≥ 3 standard deviations were removed). All the analyses were performed using SAS Version 9.4 (SAS Institute, Cary, NC).The z-scores were be calculated by the following equation:

$$Z=\frac{\left(\frac{X}{M}\right)^{L}-1}{LS}$$

If L=0,

$$Z=\ln(X/M)/S$$

where X is the TBLH BMD, L is the power transformation, M is the median value, and S is the population standard deviation[25]. Percentiles were obtained from z-scores, e.g., z-scores of -1.645, -1.282, 0, 1.282, and 1.645 correspond to the 5th, 10th, 50th, 90th and 95th percentiles, respectively.

Results

Among 10,818 children residing in seven geographically distributed centers across China, children from Beijing accounted for the largest proportion of 16.2%, while children from Ningxia accounts for the smallest proportion of 12.2% (Table 1). In addition, the age- and sex specific TBLH BMD was significant different among seven centers. Children residing in Northeast China had the highest TBLH BMD while children residing in Shandong Province had the lowest (Table 2). TBLH BMD of children by sex and height are listed in Table 3. TBLH BMD increased with age for both sexes, e.g., from 0.412 g/cm^2 at age 3 to 0.948 g/cm^2 at age 18 for boys, and from 0.399 g/cm^2 to 0.853 g/cm^2 for girls. Similarly, TBLH BMD also increased with height for boys and girls, e.g., from 0.401 at height of 100 cm to 0.977 at height of 180 cm

For peer review only - http://bmjopen.bmj.com/site/about/guidelines.xhtml

For peer review only

for boys, and from 0.396 g/cm^2 to 0.878 g/cm^2 for girls. Overall, TBLH BMD was higher in boys than in girls.

The percentiles of TBLH BMD among Chinese and U.S. children were compared and are demonstrated by Figure 1. TBLH BMD increased with age among boys and girls in both countries; however, TBLH BMD was comparatively higher in children living in U.S. than those in China. Among boys, the differences at each percentile (i.e., P3, P50, and P97) between China and the U.S. were similar in general, which was small in young ages and peaked at age 18. BMD at P3, P50, and P97 was higher in American boys than in Chinese boys, i.e., 13.1%, 13.4%, and 9.9%, respectively. There was a gradual increase in BMD among girls and boys after age 14 and 16, respectively. Furthermore, BMD for age was higher in boys than in girls in both countries.

BMD by height in China and the U.S. is shown in Figure 1. It was found that, in both countries, BMD increases with height in both sexes; however, there was a large difference in BMD for height among children from China as compared to those from U.S. Children from U.S. had a much higher BMD at each percentile than Chinese children, demonstrating the greatest difference at P50 and P3 and the least at P97. BMD values at P3 and P50 among U.S. boys were similar to BMD values at P50 and P97 among Chinese boys. The increasing rate of BMD for height was stable, with an obvious increase from height 155 cm to 175 cm among U.S. children. At each percentile (i.e., P3, P50, and P97), BMD for height was higher in U.S. children than in Chinese children, ranging from 4.9% (height: 195 cm) to 12.4% (height: 170 cm) at P3, from 8.9% (height: 195 cm) to 14.2 (height: 125 cm) at P50, and from 2.7% (height: 195 cm) to 11.4% (height: 125 cm) at P97. Among girls with low heights (e.g., <135 cm), BMD value at P3 in U.S. children was similar to BMD value at P50 in Chinese children. A clear increase in BMD for

For peer review only - http://bmjopen.bmj.com/site/about/guidelines.xhtml

height was observed from 150 cm to165 cm among U.S. girls as compared to increase in BMD from 145 to 155 cm among Chinese girls. At height greater than 155 cm, the difference in BMD became even larger. BMD peaked at 180 cm where BMD at P3 and P50 in the U.S. children were close to that at P50 and P97 of children living in China. BMD for height was also higher in boys than in girls.

Discussion

In this study, we developed sex-specific reference data using Hologic Discovery DXA scanner in a large nationwide sample of Chinese children aged 3-18 years. In addition, we compared the results from this study and references used in NHANES data, and found that BMD was lower in Chinese children than in U.S. children. To the best of our knowledge, this study is the first attempt to develop reference BMD values for Chinese children using a large nationwide pediatric population in China, and compare it with alternative available references.

China has a vast territory with large population. Disparities in their lifestyle behaviors, socioeconomic status, obesity and metabolic disorders may contribute to differences in BMD among children and adolescents. There is an urgent need to establish a unified national wide of BMD reference standards for children and adolescent, to help better evaluate their growth status. In addition, adequate peak bone mass at an early age was positively associated with lifelong health (e.g., osteoporosis at an older age) [26,27]; therefore, an accurate assessment of BMD is essential in assisting development of future preventive methods to reduce the physical and economic burden of osteoporosis.

The difference in BMD among children may be due to their different dietary patterns. Compared to children eating traditional Chinese diet (predominately consists of vegetables and

For peer review only - http://bmjopen.bmj.com/site/about/guidelines.xhtml

grains, and features plant-based protein, high in fiber, and low in cholesterol and fat), those in western countries are more likely to consume foods that consists of more meats and milk, and thus is high in energy density, fat and protein etc[28]. There are no normative reference standards of BMD available for the Chinese pediatric population based on a large dataset. The present study utilized all body and regional DXA data for establishing age-, sex-, and height specific reference values for Chinese children aged 3-18 years, and provided centile curves of BMD, which can be used in both research and clinical settings.

DXA, a widely available technology, is capable of providing regional measures of fat and lean mass. The distribution of fat and lean mass is likely to predict health outcomes[22], as shown previously in adult sample [25]. DXA measurements provide precise body composition analysis with a low radiation dose [29], and are able to detect small changes in body composition. DXA devices from different manufacturers might not give identical results due to differences in calibration and bone edge detection algorithms [30]. Therefore, to calculate reliable results one should use reference data obtained with an identical DXA device. For instance, U.S. NHANES data employed Hologic (Hologic, Inc, Bedford, MA) and GE-Lunar Inc. as the two dominant DXA manufacturers. Despite the similar DXA technology used for these manufacturers, the results of BMD and body composition can be different due to proprietary techniques used, algorithms to calculate the BMD, as well as the regions of interest, leading to various DXA values for patients who are scanned by different DXA systems[10]. To avoid the comparability problem, our study used Hologic as the only manufacturer in the current study. We used our collected data on BMD percentiles among Chinese children to compare with those among U.S. children, where BMD values were obtained from U.S. NHANES[22].

For peer review only - http://bmjopen.bmj.com/site/about/guidelines.xhtml

Our study has strengths. First, we developed a number of reference curves generated from objective measures from DXA including sex- age specific and sex- height specific TBLH BMD, providing nationwide references for Chinese children. Second, the large sample size enabled us to construct stable reference curves and extended previous studies though covering a wide-range age from 3 to 18. Third, our study followed the recommendation of ISCD and studied TBLH BMD for Chinese children.

Several limitations were noteworthy. First, even though our data is nationally and geographically distributed across China; it is not nationally representative and hence we could not generalize the results to the entire Chinese pediatric population. For example, although Ningxia Province, representative of Northwest China, has thousands of Hui population, we mainly collected data from schools including Han population. Thus, our sampled participants across China likely only represent Chinese Han children. Moreover, our developed reference curve could not be used alone as clinical criteria for the diagnosis of osteoporosis. This is because 1) low bone mass may reflect only a small part of the true burden of osteoporosis and 2) BMD could not demonstrate other important components of bone strength.

Conclusion

This is the first study to develop sex-specific references among 3-18-year-old Chinese children using the Hologic Discovery DXA scanner. The information can be used to guide clinicians improve interpretation, assessment, and monitoring of BMD results for Chinese children. However, it needs to be combined with other indicators (e.g., blood pressure, serum lipid, and calcium-phosphorus metabolism) to identify the diagnosis cut off points for osteopenia.

For peer review only - http://bmjopen.bmj.com/site/about/guidelines.xhtml

For peer review only

Acknowledgements

Author Contributions: Dr. Mi had full access to all the data in the study and takes responsibility for the integrity of the data and the accuracy of the data analysis. Study concept and design: Mi. Acquisition, analysis, or interpretation of data: Sun, Liu, Yan, Xi, Xiong, Ding, Jie Mi. Huang, Mi. Drafting of the manuscript: Liu, Wang. Critical revision of the manuscript for important intellectual content: Liu, Wang, Heymsfield, Mi. Statistical analysis: Liu, Huang. Obtained funding: Mi. Administrative, technical, or material support: Sun, Liu, Yan, Xi, Xiong, Ding, Mi. Study supervision: Mi.

Conflict of Interest Disclosures: All authors declared no conflicts of interest.

Data sharing statement: Extra data is available by emailing Jiemi@vip.163.com.

Funding/support: The study was supported by grants from the "Twelfth Five-Year Plan" of the China National Science and Technology (2012BAI03B03), the Beijing Health System Leading Talent Grant (2009-1-08), the Beijing Training Project for the Leading Talents in Science and Technology (2011LJ07), and the content is solely the responsibility of the authors and does not necessarily represent the official views of the funders.

Role of the Funder/Sponsor: The funding sources had no role in the design and conduct of the study; collection, management, analysis, and interpretation of the data; preparation, review, or approval of the manuscript; and decision to submit the manuscript for publication.

For peer review only - http://bmjopen.bmj.com/site/about/guidelines.xhtml

References

1. Leslie WD, Morin SN: Osteoporosis epidemiology 2013: Implications for diagnosis, risk assessment and treatment. *Curr Opin Rheumatol*. 2014;26(4):440-446.

2. Liu ZH, Zhao YL, Ding GZ, Zhou Y. Epidemiology of primary osteoporosis in China. Osteoporos Int. 1997;7 Suppl 3:S84–S87.

3. Lin X, Xiong D, Peng YQ, et al. Epidemiology and management of osteoporosis in the People's Republic of China: current perspectives. Clin Interv Aging. 2015;10:1017-1033.

4. Mauck KF, Clarke BL: Diagnosis, Screening, Prevention, and Treatment of Osteoporosis. Mayo Clin Proc. 2006; 81(5):662-672.

5. Editorial Board of Osteoporosis Prevention and Treatment (China White Paper), China Health Promotion Foundation. White paper on osteoporosis. Chin J Health Manage. 2009. 3(3): 148-154.

6. Hansen MA, Overgaard K, Riis BJ, *et al*. Role of peak bone mass and bone loss in postmenopausal osteoporosis: 12 year study. BMJ. 1991;303:961-4.

7. Matkovic V, Jelic T, Wardlaw GM, et al. Timing of peak bone mass in caucasian females and its implication for the prevention of osteoporosis. Inference from a cross-sectional model. J Clin Invest 1994;93:799-808.

8. Ogle GD, Allen JR, Humphries IRJ, et al. Body-composition assessment by dual-energy x-ray absorptiometry in subjects aged 4-26 y. Am J Clin Nutr 1995;61:746-753.

9. Ward KA, Ashby RL, Roberts SA, Adams JE, Zulf Mughal M.UK reference data for the Hologic QDR Discovery dual-energy X ray absorptiometry scanner in healthy children and young adults aged 6-17 years. Arch Dis Child. 2007; 92(1):53-59.

For peer review only - http://bmjopen.bmj.com/site/about/guidelines.xhtml

For peer review only

10. Fan B, Shepherd JA, Levine MA, et al. National Health and Nutrition Examination Survey whole-body dual-energy X-ray absorptiometry reference data for GE Lunar systems. J Clin Densitom. 2014;17(3):344-377.

11. Genant HK, Engelke K, Fuerst T, Glüer CC, Grampp S, Harris ST, et al. Noninvasive assessment of bone mineral and structure: State of the art. J Bone Miner Res 11(6):707–30. 1996.

12. Lohman TG, Chen Z. Dual-energy x-ray absorptiometry. In: Heymsfield SB, Lohman T, Wang ZM, Going SB, eds. Human body composition, 2nd ed. Champaign, IL: Human Kinetics Press. 2005.

13. Njeh CF, Fuerst T, Hans D, Blake GM, Genant HK. Radiation exposure in bone mineral density assessment. Appl Radiat Isot.1999; 50(1):215–236.

14. Thomas SR, Kalkwarf HJ, Buckley DD, Heubi JE. Effective dose of dual-energy x-ray absorptiometry scans in children as a function of age. J Clin Densitom.2005; 8(4):415–422.

15. Wahner HW, Fogelman I. The evaluation of osteoporosis: Dual energy x-ray absorptiometry in clinical practice. London: Martin Dunitz Ltd. 1994.

16. WHO. Assessment of fracture risk and its application to screening for postmenopausal osteoporosis: report of a WHO study group. WHO Technical Report Series 843, WHO, Geneva 1994.

17. Bachrach LK, Hastie T, Wang M-C, et al. Bone mineral acquisition in healthy Asian, Hispanic, black, and Caucasian youth: a longitudinal study. J Clin Endocrinol Metab 1999;84:4702–4712.

For peer review only - http://bmjopen.bmj.com/site/about/guidelines.xhtml

18. Neu C, Manz F, Rauch F, et al. Bone densities and bone size at the distal radius in healthy children and adolescents: a study using peripheral quantitative computed tomography. Bone 2001; 28:227–232.

19. Schoenau E, Neu C, Rauch F, et al. Gender-specific pubertal changes in volumetric cortical bone mineral density at the proximal radius. Bone 2002; 31:110–113.

20. Nelson D, Simpson P, Johnson C, et al. The accumulation of whole body skeletal mass in third- and fourth-grade children: effects of age, gender, ethnicity and body composition. Bone 1997;20:73–78.

21. Wang MC, Aguirre M, Bhudhikanok GS, et al. Bone mass and hip axis length in healthy Asian, black, Hispanic, and white American youths. J Bone Miner Res 1997;12:1922–1935.

22. Kelly TL, Wilson KE, Heymsfield SB. Dual energy X-ray absorptiometry body composition reference values from NHANES. PLoS One. 2009; 4(9):e7038.

23. Lloyd-Jones DM, Hong Y, Labarthe D, et al. Defining and setting national goals for cardiovascular health promotion and disease reduction: the American Heart Association's strategic Impact Goal through 2020 and beyond. Circulation. 2010;121:586–613.

24. Cole TJ, Green PJ. Smoothing reference centile curves: the LMS method and penalized likelihood. Stat Med. 1992;11(10):1305-1309.

25. Pan H, Cole TJ. A comparison of goodness of fit tests for age-related reference ranges. Stat Med. 2004; 23(11):1749-1765.

26. Heaney RP, Abrams S, Dawson-Hughes B, Looker A, Marcus R, Matkovic V, Weaver C. Peak bone mass. Osteoporos Int. 2000;11:985–1009.

For peer review only

27. Rizzoli R, Bianchi ML, Garabedian M, McKay HA, Moreno LA. Maximizing bone mineral mass gain during growth for the prevention of fractures in the adolescents and the elderly. Bone. 2010; 46:294–305.

28. Xie QY. Cultural difference between the East and the West. Canadian Social Science 2007; 3(5): 114-117.

29. Mazess RB, Barden HS, Bisek JP, et al. Dual-energy absorptiometry for total body and regional bone mineral and soft tissue composition. Am J Clin Nutr 1990;51:1106–1112.

30. Bonnick SL. Bone densitometry in clinical practice. Totowa, New Jersey: Humana Press Inc., 1998.

For peer review only

For peer review only - http://bmjopen.bmj.com/site/about/guidelines.xhtml

Table 1. The distribution of coefficient of variation (CV, %) for lumbar spine, hip joint, and femoral neck by study location, the China Child and Adolescent Cardiovascular Health Study (n=10 808)

Province	Lumbar spine	Hip Joint	Femoral neck
Beijing	0.88%	1.42%	1.91%
Shanghai	0.97%	1.34%	2.33%
Tianjin	1.06%	1.02%	1.99%
Chongqing	0.59%	1.62%	2.05%
Jilin	1.58%	1.79%	1.39%
Shandong	1.11%	1.79%	1.97%
Ningxia	1.25%	1.07%	1.89%

For peer review only

For peer review only - http://bmjopen.bmj.com/site/about/guidelines.xhtml

Table 2.Geographical distribution of sample, The China Child and Adolescent Cardiovascular Health (CCACH) (n=10 808)

Geographical area	Province	Latitude	N	%	TBLH BMD
Northeast	Jilin	N 40°52’~46°18’	1661	15.4	0.785±0.160
North China	Beijing	N39°26’~41°03’	1739	16.1	0.734±0.151
	Tianjin	N38°33’~40°15’	1653	15.3	
Northwest	Ningxia	N35°14’~39°23’	1315	12.2	0.743±0.129
East China	Shanghai,	N30°40’~31°53’	1613	14.9	0.756±0.121
	Shandong	N34°25’~38°23’	1437	13.3	0.722±0.139
Southwest	Chongqing	N28°10’~32°13’	1400	12.9	0.778±0.143
F*					191.02
P					<0.0001

Abbreviations: TBLH, total body less head; BMD, bone mineral density.
*Analysis of covariance, adjusted for age and sex.

For peer review only

Table 3. TBLH BMD (g/cm^2) of children aged 3 to 18 years by sex and height, The China Child and Adolescent Cardiovascular Health (n=10 808)

Age,Height	Male							Female						
	Sample size	Mean±SD	TBLH BMD Percentiles					Sample size	Mean±SD	TBLH BMD Percentiles				
			5th	10th	50th	90th	95th			5th	10th	50th	90th	95th
Age(years)														
3	110	0.412±0.030	0.350	0.360	0.395	0.434	0.446	122	0.399±0.025	0.349	0.357	0.385	0.417	0.426
4	269	0.444±0.037	0.373	0.383	0.421	0.463	0.476	267	0.426±0.032	0.366	0.374	0.406	0.441	0.452
5	322	0.479±0.039	0.409	0.420	0.463	0.511	0.526	259	0.470±0.036	0.401	0.411	0.449	0.492	0.505
6	408	0.531±0.046	0.445	0.458	0.505	0.559	0.576	376	0.515±0.047	0.434	0.446	0.491	0.542	0.558
7	401	0.582±0.050	0.489	0.503	0.557	0.618	0.636	359	0.557±0.049	0.468	0.482	0.535	0.594	0.612
8	399	0.610±0.051	0.523	0.538	0.596	0.663	0.684	333	0.595±0.055	0.498	0.514	0.575	0.642	0.662
9	332	0.644±0.052	0.547	0.564	0.627	0.699	0.721	340	0.629±0.061	0.526	0.544	0.612	0.687	0.710
10	345	0.673±0.059	0.571	0.589	0.656	0.733	0.757	303	0.670±0.072	0.556	0.576	0.652	0.734	0.758
11	327	0.709±0.068	0.597	0.615	0.687	0.770	0.795	293	0.722±0.071	0.593	0.615	0.696	0.784	0.810
12	352	0.748±0.071	0.628	0.648	0.725	0.814	0.842	290	0.765±0.069	0.635	0.658	0.743	0.834	0.861
13	319	0.798±0.075	0.665	0.687	0.771	0.867	0.898	274	0.800±0.071	0.673	0.696	0.781	0.873	0.900
14	299	0.845±0.075	0.704	0.728	0.818	0.923	0.956	298	0.818±0.061	0.701	0.724	0.807	0.897	0.924
15	440	0.877±0.085	0.737	0.762	0.858	0.971	1.006	475	0.829±0.062	0.720	0.741	0.823	0.911	0.937
16	540	0.916±0.096	0.763	0.789	0.891	1.011	1.048	571	0.843±0.068	0.734	0.755	0.835	0.922	0.949
17	405	0.953±0.094	0.791	0.819	0.927	1.054	1.094	456	0.853±0.068	0.745	0.766	0.845	0.933	0.960
18	241	0.948±0.093	0.808	0.836	0.949	1.081	1.123	293	0.853±0.068	0.751	0.771	0.850	0.938	0.966
Height(cm)														
100	126	0.401±0.025	0.366	0.372	0.395	0.422	0.431	171	0.396±0.024	0.360	0.366	0.390	0.417	0.425
105	169	0.434±0.024	0.386	0.393	0.420	0.452	0.462	200	0.428±0.025	0.382	0.389	0.416	0.447	0.457
110	227	0.463±0.028	0.410	0.418	0.449	0.486	0.498	175	0.459±0.030	0.405	0.413	0.443	0.480	0.492
115	275	0.504±0.037	0.437	0.447	0.483	0.526	0.540	278	0.492±0.035	0.431	0.439	0.474	0.519	0.534
120	313	0.538±0.044	0.467	0.478	0.519	0.570	0.586	284	0.534±0.042	0.458	0.468	0.509	0.563	0.582
125	358	0.577±0.045	0.497	0.509	0.555	0.613	0.632	336	0.566±0.050	0.487	0.499	0.546	0.611	0.634
130	363	0.604±0.046	0.524	0.537	0.588	0.652	0.674	299	0.600±0.053	0.513	0.526	0.579	0.653	0.679
135	330	0.642±0.053	0.550	0.564	0.620	0.691	0.714	256	0.630±0.057	0.534	0.548	0.607	0.689	0.718
140	312	0.667±0.054	0.573	0.588	0.649	0.726	0.752	254	0.662±0.068	0.560	0.576	0.642	0.732	0.764
145	258	0.692±0.064	0.595	0.611	0.677	0.761	0.789	277	0.722±0.079	0.597	0.615	0.690	0.787	0.821
150	263	0.726±0.068	0.620	0.637	0.708	0.798	0.829	489	0.784±0.078	0.645	0.665	0.747	0.848	0.882
155	254	0.769±0.075	0.651	0.669	0.745	0.845	0.878	830	0.813±0.070	0.688	0.709	0.793	0.892	0.923
160	336	0.828±0.087	0.686	0.706	0.788	0.897	0.934	838	0.829±0.065	0.718	0.739	0.821	0.913	0.942
165	514	0.859±0.090	0.721	0.742	0.831	0.948	0.989	444	0.855±0.066	0.741	0.762	0.843	0.932	0.959

For peer review only - http://bmjopen.bmj.com/site/about/guidelines.xhtml

For peer review only

170	670	0.896±0.086	0.753	0.776	0.871	0.997	1.040	120	0.878±0.076	0.761	0.783	0.864	0.953	0.980
175	432	0.930±0.095	0.784	0.808	0.908	1.041	1.088	…	…	…	…	…	…	…
180	222	0.977±0.103	0.813	0.838	0.943	1.084	1.134	…	…	…	…	…	…	…

Abbreviations: TBLH, total body less head; BMD, bone mineral density; SD, standard deviation.

For peer review only

For peer review only - http://bmjopen.bmj.com/site/about/guidelines.xhtml

Figure 1. The percentiles of TBLH BMD among Chinese and U.S. children by age and height

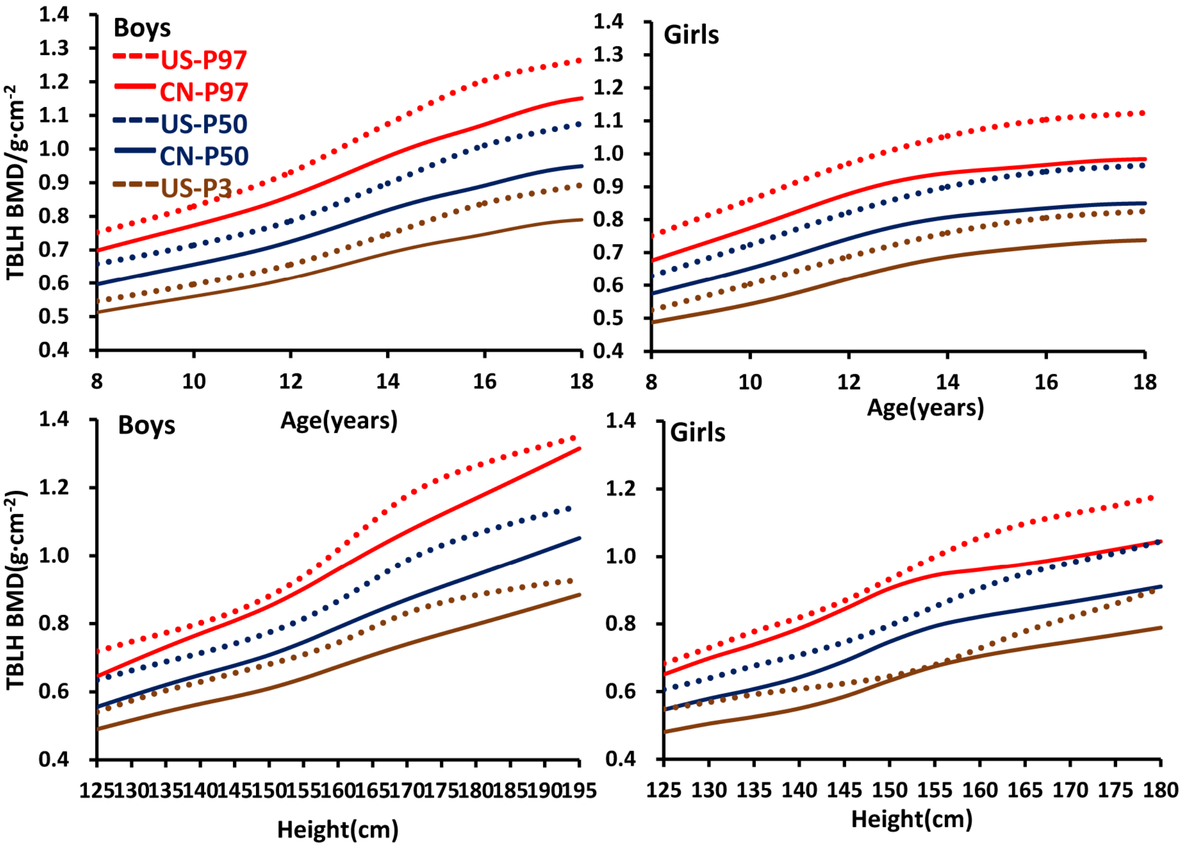

For peer review only - http://bmjopen.bmj.com/site/about/guidelines.xhtml

Supplemental Table 1. LMS parameters for the reference centiles of total body less head BMD /g·cm^{-2} by age in boys and girls

Age/yrs	Boys			Girls		
	L	M	S	L	M	S
3.5	-0.251	0.399	0.073	-0.480	0.385	0.061
4.0	-0.251	0.421	0.074	-0.417	0.406	0.064
4.5	-0.251	0.443	0.075	-0.355	0.427	0.067
5.0	-0.251	0.463	0.076	-0.293	0.449	0.070
5.5	-0.251	0.482	0.077	-0.230	0.470	0.073
6.0	-0.251	0.505	0.078	-0.166	0.491	0.076
6.5	-0.251	0.531	0.079	-0.101	0.513	0.079
7.0	-0.251	0.557	0.080	-0.035	0.535	0.082
7.5	-0.251	0.579	0.081	0.030	0.555	0.084
8.0	-0.251	0.596	0.082	0.092	0.575	0.087
8.5	-0.251	0.612	0.083	0.151	0.594	0.089
9.0	-0.251	0.627	0.084	0.205	0.612	0.091
9.5	-0.251	0.641	0.085	0.253	0.632	0.093
10.0	-0.251	0.656	0.085	0.294	0.652	0.094
10.5	-0.251	0.671	0.086	0.330	0.673	0.095
11.0	-0.251	0.687	0.087	0.358	0.696	0.095
11.5	-0.251	0.705	0.088	0.378	0.720	0.094
12.0	-0.251	0.725	0.089	0.388	0.743	0.092
12.5	-0.251	0.747	0.090	0.388	0.763	0.090
13.0	-0.251	0.771	0.091	0.375	0.781	0.088
13.5	-0.251	0.795	0.092	0.351	0.796	0.086
14.0	-0.251	0.818	0.093	0.314	0.807	0.084
14.5	-0.251	0.840	0.094	0.266	0.816	0.082
15.0	-0.251	0.858	0.095	0.208	0.823	0.080
15.5	-0.251	0.874	0.096	0.143	0.829	0.079
16.0	-0.251	0.891	0.096	0.071	0.835	0.078
16.5	-0.251	0.910	0.097	-0.006	0.841	0.078
17.0	-0.251	0.927	0.098	-0.087	0.845	0.077
17.5	-0.251	0.941	0.099	-0.171	0.848	0.077
18.0	-0.251	0.949	0.100	-0.257	0.850	0.076
18.5	-0.251	0.955	0.101	-0.345	0.851	0.076

For peer review only

Supplemental Table 2. LMS parameters for the reference centiles of total body less head BMD /g·cm^{-2} by height in boys and girls

Height/cm	Boys			Girls		
	L	M	S	L	M	S
95	-1.272	0.374	0.045	-0.926	0.371	0.047
100	-1.272	0.395	0.050	-1.168	0.390	0.050
105	-1.272	0.420	0.054	-1.413	0.416	0.054
110	-1.272	0.449	0.059	-1.665	0.443	0.058
115	-1.272	0.483	0.064	-1.871	0.474	0.064
120	-1.272	0.519	0.068	-1.980	0.509	0.072
125	-1.272	0.555	0.072	-2.008	0.546	0.078
130	-1.272	0.588	0.076	-1.950	0.579	0.083
135	-1.272	0.620	0.079	-1.776	0.607	0.088
140	-1.272	0.649	0.082	-1.499	0.642	0.093
145	-1.272	0.677	0.085	-1.146	0.690	0.096
150	-1.272	0.708	0.087	-0.764	0.747	0.095
155	-1.272	0.745	0.090	-0.417	0.793	0.089
160	-1.272	0.788	0.093	-0.159	0.821	0.083
165	-1.272	0.831	0.095	-0.004	0.843	0.078
170	-1.272	0.871	0.097	0.096	0.864	0.077
175	-1.272	0.908	0.098	0.187	0.887	0.076
180	-1.272	0.943	0.100	0.281	0.910	0.075
185	-1.272	0.979	0.101	-	-	-
190	-1.272	1.015	0.102	-	-	-
195	-1.272	1.052	0.103	-	-	-

For peer review only

Supplemental Figure 1. Geographical distribution of sampled pediatric participants

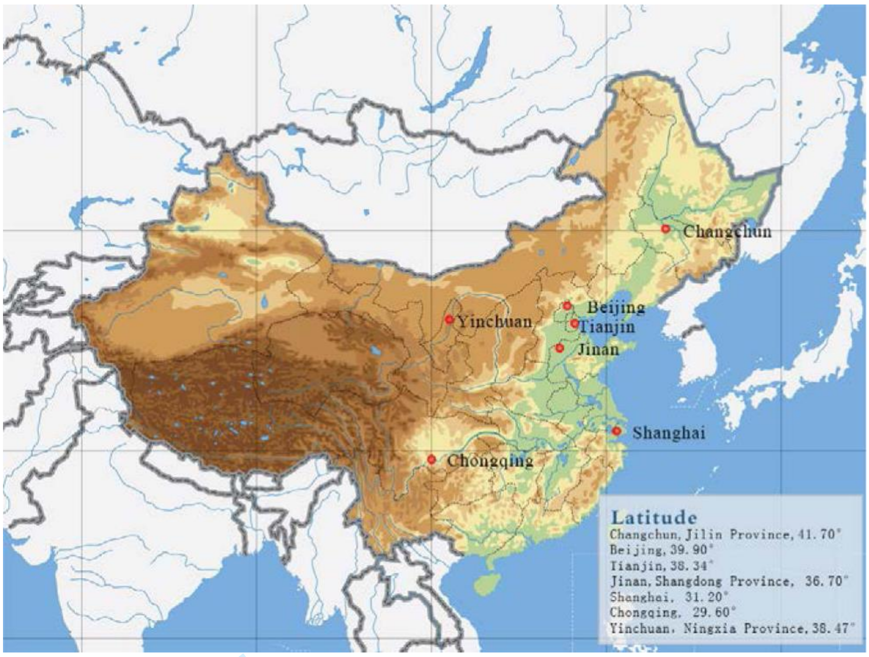

For peer review only

STROBE Statement—checklist of items that should be included in reports of observational studies

	Item No	Recommendation
**Title and abstract**	1	(*a*) Indicate the study's design with a commonly used term in the title or the abstract
Page 2		(*b*) Provide in the abstract an informative and balanced summary of what was done and what was found
**Introduction**		
Background/rationale Page 4	2	Explain the scientific background and rationale for the investigation being reported
Objectives Page 5	3	State specific objectives, including any prespecified hypotheses
**Methods**		
Study design Page 5	4	Present key elements of study design early in the paper
Setting Page 6	5	Describe the setting, locations, and relevant dates, including periods of recruitment, exposure, follow-up, and data collection
Participants Page 6	6	(*a*) *Cohort study*—Give the eligibility criteria, and the sources and methods of selection of participants. Describe methods of follow-up<br>*Case-control study*—Give the eligibility criteria, and the sources and methods of case ascertainment and control selection. Give the rationale for the choice of cases and controls<br>*Cross-sectional study*—Give the eligibility criteria, and the sources and methods of selection of participants
		(*b*) *Cohort study*—For matched studies, give matching criteria and number of exposed and unexposed<br>*Case-control study*—For matched studies, give matching criteria and the number of controls per case
Variables Page 7	7	Clearly define all outcomes, exposures, predictors, potential confounders, and effect modifiers. Give diagnostic criteria, if applicable
Data sources/ measurement Page 6	8*	For each variable of interest, give sources of data and details of methods of assessment (measurement). Describe comparability of assessment methods if there is more than one group
Bias Page 6	9	Describe any efforts to address potential sources of bias
Study size Page 6	10	Explain how the study size was arrived at
Quantitative variables Page 7	11	Explain how quantitative variables were handled in the analyses. If applicable, describe which groupings were chosen and why
Statistical methods Page 7	12	(*a*) Describe all statistical methods, including those used to control for confounding
		(*b*) Describe any methods used to examine subgroups and interactions
		(*c*) Explain how missing data were addressed
		(*d*) *Cohort study*—If applicable, explain how loss to follow-up was addressed<br>*Case-control study*—If applicable, explain how matching of cases and controls was addressed<br>*Cross-sectional study*—If applicable, describe analytical methods taking account of sampling strategy
		(*e*) Describe any sensitivity analyses

For peer review only - http://bmjopen.bmj.com/site/about/guidelines.xhtml

**Results**		
Participants<br>Page 8	13*	(a) Report numbers of individuals at each stage of study—eg numbers potentially eligible, examined for eligibility, confirmed eligible, included in the study, completing follow-up, and analysed
		(b) Give reasons for non-participation at each stage
		(c) Consider use of a flow diagram
Descriptive data<br>Page 8	14*	(a) Give characteristics of study participants (eg demographic, clinical, social) and information on exposures and potential confounders
		(b) Indicate number of participants with missing data for each variable of interest
		(c) *Cohort study*—Summarise follow-up time (eg, average and total amount)
Outcome data<br>Page 8	15*	*Cohort study*—Report numbers of outcome events or summary measures over time
		Case-control study—Report numbers in each exposure category, or summary measures of exposure
		Cross-sectional study—Report numbers of outcome events or summary measures
Main results<br>Page 9	16	(*a*) Give unadjusted estimates and, if applicable, confounder-adjusted estimates and their precision (eg, 95% confidence interval). Make clear which confounders were adjusted for and why they were included
		(*b*) Report category boundaries when continuous variables were categorized
		(*c*) If relevant, consider translating estimates of relative risk into absolute risk for a meaningful time period
Other analyses<br>Page 10	17	Report other analyses done—eg analyses of subgroups and interactions, and sensitivity analyses
**Discussion**		
Key results<br>Page 10	18	Summarise key results with reference to study objectives
Limitations<br>Page 12	19	Discuss limitations of the study, taking into account sources of potential bias or imprecision. Discuss both direction and magnitude of any potential bias
Interpretation<br>Page 12	20	Give a cautious overall interpretation of results considering objectives, limitations, multiplicity of analyses, results from similar studies, and other relevant evidence
Generalisability<br>Page 12	21	Discuss the generalisability (external validity) of the study results
**Other information**		
Funding<br>Page 13	22	Give the source of funding and the role of the funders for the present study and, if applicable, for the original study on which the present article is based

*Give information separately for cases and controls in case-control studies and, if applicable, for exposed and unexposed groups in cohort and cross-sectional studies.

Note: An Explanation and Elaboration article discusses each checklist item and gives methodological background and published examples of transparent reporting. The STROBE checklist is best used in conjunction with this article (freely available on the Web sites of PLoS Medicine at http://www.plosmedicine.org/, Annals of Internal Medicine at http://www.annals.org/, and Epidemiology at http://www.epidem.com/). Information on the STROBE Initiative is available at www.strobe-statement.org.

For peer review only

Bone mineral density reference standards for Chinese children aged 3-18: Cross-sectional results of the 2013-2015 China Child and Adolescent Cardiovascular Health (CCACH)

Journal:	*BMJ Open*
Manuscript ID	bmjopen-2016-014542.R1
Article Type:	Research
Date Submitted by the Author:	08-Mar-2017
Complete List of Authors:	Liu, Junting; Capital Institute of Pediatrics, Department of Epidemiology<br>Wang, Liang; East Tennessee State University, ; East Tennessee State University<br>Sun, Jinghui ; The First Hospital of Jilin University<br>Liu, Gongshu; Tianjin Women's and Children's Health Center,<br>Yan, Weili; Fudan Univ,<br>Xi, Bo; Shandong University<br>Xiong, Feng; Children's Hospital of Chongqing Medical University, Department of Endocrinology<br>Ding, Wenqing; Ningxia Medical University, Department of Public Health<br>Huang, Guimin; Capital Institute of Pediatrics, Department of Epidemiology<br>Heymsfield, Steven; Pennington Biomedical Research Center, Metabolism - Body Composition<br>Mi, Jie; Capital Institute of Pediatrics, Department of Epidemiology
**Primary Subject Heading**:	Epidemiology
Secondary Subject Heading:	Paediatrics, Public health
Keywords:	BMD, DXA, Children, China

SCHOLARONE™ Manuscripts

For peer review only - http://bmjopen.bmj.com/site/about/guidelines.xhtml

Bone mineral density reference standards for Chinese children aged 3-18: Cross-sectional results of the 2013-2015 China Child and Adolescent Cardiovascular Health (CCACH)

Junting Liu[1#], MS, Liang Wang[2#],MD, DrPH, MPH, Jinghui Sun, MD[3†],Gongshu Liu, MD [4†] ,Weili Yan, PhD[5†],Bo Xi[6†], PhD, Feng Xiong[7†], MS, Wenqing Ding[8†],PhD, Guimin Huang[1], MS, Steven B. Heymsfield, PhD,[9] Jie Mi[1*],MD,PhD[1]

[# †]These authors contributed equally to the work.

[1]Department of Epidemiology, Capital Institute of Pediatrics, Beijing 100020, China
[2]Department of Biostatistics and Epidemiology, College of Public Health, East Tennessee State University, TN 37614, USA
[3]Department of Cardiovascular Medicine, The First Hospital of Jilin University, Changchun 130021, China
[4]Project Office, Tianjin Women's and Children's Health Center, Tianjin 300070, China
[5]Department of Clinical Epidemiology, Children's Hospital of Fudan University, Shanghai 200433, China
[6]Department of Public Health, Shandong University, Jinan 250012, China
[7]Department of Endocrinology, Children's Hospital of Chongqing Medical University, Chongqing 400014, China
[8]Department of Public Health, Ningxia Medical University, Yinchuan 750003, China
[9]Department of Metabolism-Body Composition, Pennington Biomedical Research Center, Baton Rouge, LA 70808, USA

Email:
Junting Liu, Junting_liu@163.com; Liang Wang, wangl2@etsu.edu; Jinghui Sun, 13756661617@126.com; Gongshu Liu, Liugongshu727@163.com; Weili Yan, yanwl@fudan.edu.cn; Bo Xi, swei006@163.com; Feng Xiong, xiongfw2013@163.com; Wenqing Ding,13519598269@163.com; Guimin Huang, guiminhuang@163.com; Steven B. Heymsfield, steven.heymsfield@pbrc.edu; Jie Mi, Jiemi@vip.163.com

Corresponding author
Jie Mi, Department of Epidemiology, Capital Institute of Pediatrics, 2 Yabao Road, Chaoyang District ,Beijing 100020, China. Tel: +86-10-85695591; Fax: +86-10-85632799; jiemi@vip.163.com.

Word count for main text: 3132

Number of table: 3

Number of figure: 2

Number of supplemental table: 10

Number of supplemental figure: 1

For peer review only

Abstract

Objectives: No nationwide pediatric reference standards for bone mineral density (BMD) are available in China. We aimed to provide sex-specific BMD reference values for Chinese children and adolescents (3-18 years).

Methods: Data (10,818 participants aged 3-18 years) were obtained from cross-sectional surveys of the China Child and Adolescent Cardiovascular Health (CCACH) in 2015, including four municipality cities and three provinces. BMD was measured using Hologic Discovery Dual Energy X-ray Absorptiometry (DXA) scanner. The DXA measures were modeled against age, with height as an independent variable. The LMS statistical method using a curve fitting procedure was used to construct reference smooth cross sectional centile curves for dependent versus independent variables.

Results: Children residing in Northeast China had the highest total body less head (TBLH) BMD while children residing in Shandong Province had the lowest values. Among children, TBLH BMD was higher for boys as compared to girls; but it increased with age and height in both sexes. Furthermore, TBLH BMD was higher among U.S. children as compared to Chinese children. There was a large difference in BMD for height among children from the two countries. U.S. children had a much higher BMD at each percentile (P) than Chinese children, with the difference being the largest at P50 and P3 and being the smallest at P97.

Conclusions: This is the first study to present a sex-specific reference dataset for 3-18 year old Chinese children. The data can help clinicians improve interpretation, assessment, and monitoring of densitometry results.

Key words: BMD, DXA, China, Children

For peer review only

For peer review only - http://bmjopen.bmj.com/site/about/guidelines.xhtml

Strengths and limitations of this study

- It is the first study to present a sex-specific reference dataset for 3-18 year old Chinese children.
- A number of reference curves were developed using objective measures from DXA.
- The large sample size enabled us to construct stable reference curves and extended previous studies though covering a wide-range age from 3 to 18.
- The data we collected were not nationally representative and hence we could not generalize the results to the entire Chinese pediatric population.
- Our developed reference curve could not be used alone as clinical criteria for the diagnosis of pediatric osteoporosis.

For peer review only

For peer review only - http://bmjopen.bmj.com/site/about/guidelines.xhtml

Introduction

Osteoporosis, as a widespread metabolic bone disease, has become a global public health problem. More than 200 million people worldwide and 10 million people in the United States (U.S.) were afflicted with osteoporosis in 2013.[1] In China, the population with osteoporosis in 1997 was 83.9 million, which is projected to increase to 212 million by 2050.[2] The burdens associated with osteoporosis are heavy, with the most common being the fractures. It has been estimated that the treatment cost for hip fracture, in China, would be more than 85 billion Chinese Yuan (i.e., about 13 billion U.S. dollars) in 2020, and 1800 billion Chinese Yuan (i.e., about 271 U.S. dollars) in 2050.[3]

Peak bone mass is an important determinant for osteoporotic fracture risk. A higher peak bone mass is associated with lower risk of osteoporotic fractures later in life.[4] In general, individuals have peak bone mass in their early 20s; however, it may occur earlier in women.[5,6] Thus, investigating the pattern of bone mineralization in children is of great interest. Dual-energy X-ray absorptiometry (DXA) has been recommended by the International Society for Clinical Densitometry (ISCD)[7] as a current criterion for measuring body mineral content (BMC) and bone mineral density (BMD). This measurement method has high precision, accuracy, speed, low radiation exposure, ease of use, and strong correlation with established methods.[8-14]

Pediatric osteoporosis is defined as having a history of pathologic fractures and low bone mineral content or density.[15] To diagnose pediatric osteoporosis, DXA is the most widely used bone densitometry technique in children.[7] Although 60% of peak bone mass is genetically determined, the increase of bone mass can be influenced by other factors, including intake of dietary products (e.g., calcium and vitamin D) and weight-bearing physical activity (e.g., jumping).[16]

For peer review only - http://bmjopen.bmj.com/site/about/guidelines.xhtml

While diagnosing children can be problematic due to the lack of a widely used reference, the Bone Mineral Density in Childhood Study (BMDCS) has developed an online BMD z-score calculator for health care providers (https://bmdcs.nichd.nih.gov/zscore.htm). Widespread reference for multiple diagnostic measurements is important to ensure correct diagnosis among children so that treatment and management for osteoporosis can begin. Uniformity in diagnosis is also imperative for public health so that epidemiologic studies can be done across geographic regions, ethnicities, and social economic status to properly inform prevention practices. Moon et al. (2016) studied the accuracy of parental recall of children's fractures. They determined that, of 660 parents, 207 children had previous fractures. Of these, 21% were reported incorrectly, indicating a need to have a reporting system for fractures so as to better identify osteoporosis in children.[17] A study published in 2014, created reference data for GE Lunar systems from the U.S. National Health and Nutrition Examination Survey (NHANES). To do this they took data from 8056 participants under the age of 20 and created reference curves by age, sex, and ethnicity.[10] Kinning et al. (2016) discovered that the rearrangement of chromosomes 4:20 is associated with childhood osteoporosis, highlighting the need to look further into genetic predispositions for diseases.[18]

For children, DXA can be used to measure whole body bone area (cm^2) and whole body BMC (g), leading to BMD, i.e., BMC divided by bone area (g/cm^2) which indicates the pediatric bone status. The use and correct identification of indicators for BMD is particularly important; which requires appropriate reference data that are matched for multiple factors including sex,[19-21] chronological age, height, weight, pubertal development and ethnicity.[19,22,23, 24] The preferred skeletal sites are lumbar spine (L1–4) and total body, excluding the head.[25] The cranium should not be included for the total body scan analysis, because the head constitutes a large portion of

For peer review only - http://bmjopen.bmj.com/site/about/guidelines.xhtml

the total body bone mass but changes little with growth, activity, or disease. Thus, including the skull is likely to mask gains or losses at other skeletal sites.[26]

Childhood and adolescence are critical periods for bone growth, while half of peak bone mass is developed in childhood. If BMD could be evaluated in childhood and adolescence, and children with low BMD could be intervened, then peak bone mass can be improved obviously in adulthood, osteoporosis occurrence can be delayed, and severity of osteoporosis can be reduced. A number of studies have shown that optimal BMC and BMD in childhood and adolescence would reduce risk of osteoporotic fracture several decades later.[27] To date, no pediatric reference standards for BMD are available in China. Furthermore, utilization of U.S. measures based on reference values derived from the U.S. NHANES data might not be applicable for Chinese pediatric population. Therefore, it is critically important and urgent to develop reference values of BMD for Chinese children based upon data gathered from Chinese children. The reference standards can help clinicians have a better estimation of the percentile on which Chinese children's DXA-measured bone parameter falls in reference to their age or height. The aim of this study was twofold: 1) to develop sex-specific reference percentile curves for Chinese children based on a large, nationwide sample; and 2) to compare our findings with those of the study conducted by Kelly and colleagues[28] using U.S. NHANES data.

Methods

Participants

The American Heart Association defined a new concept of "ideal cardiovascular health (CVH)" for American adults and children in 2010,[29] which included four health behaviors (nonsmoking, physically active, normal body mass index (BMI), healthy diet) and three health

For peer review only - http://bmjopen.bmj.com/site/about/guidelines.xhtml

factors (normal blood pressure, total cholesterol, fasting glucose). Based on these seven health behaviors and factors, the construct of CVH comprehensively combined the traditional risk factors proposed by the Framingham Heart Study, as well as lifestyle behaviors, emphasizing the importance of overall strategies in achieving optimal CV health. Our data were obtained from the China Child and Adolescent Cardiovascular Health Study (CCACHs). It is a large and nationwide ongoing study designed to examine CVH, BMD, body composition, and daily sodium intake among Chinese children ages 3-18 (Figure 1). Stratified cluster random sampling method was used to extract 100 children per age group in each cluster (50 boys and 50 girls). Because the majority of children are Han, accounting for 90% of the total Chinese pediatric population, CCACHs mainly collected CVH data among Han children. Children were recruited from seven provinces (centers) covering geographical areas of North, East, Northeast, Northwest, and Southwest of China, across latitude 29.6° north to latitude 41.7° north (Supplement Figure 1). The exclusion criteria were: (1) inability to give informed consent, (2) any condition, or use of any drug known to affect bone health, (3) non-removable objects (e.g., prostheses, implants, or casts), (4) body weight greater than 204 kg, height greater than 197.5 cm, or weight or height beyond the range of measure of machine, (5) participation in research involving ionizing radiation in the past year, (6) no history of fracture, (7) some chronic diseases, and (8) pregnancy. Data were collected on 11,057 children, out of which 109 were excluded due to kidney or thyroid disease and 130 were excluded because of implausible values of weight (light or heavy, BMI $\geq$ 3 SDs). Consequently, 10,818 children (5,509 males and 5,309 females) were used for the final analysis.

For peer review only - http://bmjopen.bmj.com/site/about/guidelines.xhtml

The study was approved by the Research Ethics Committee of Capital Institute of Pediatrics of China. The written informed consent documents for children were obtained from their parents or guardians.

--Figure 1 about here--

DXA acquisition and quality control

The whole-body scans were performed using Hologic Discovery (A, W, and Wi) fan-beam densitometers (Hologic, Inc., Bedford, Massachusetts, USA). The coefficient of variation (CV, %) of A, W and Wi were 0.471%, 0.302% and 0.358% respectively. Following recommendations of the ISCD, a high level of quality control was maintained throughout the DXA data collection and scan analysis. The specific procedures were as follows: (1) Standard phantom was checked before scanning the participants and calibrating DXA machines every morning; (2) an operator of each study location was trained and administered by the same technician certified by ISCD. The training materials included the ISCD official technician hands-on training materials and manufacturer's handbook including testing procedures and operation methods; (3) at each center, lumbar spine and hip joints of 15 participants were scanned for 3 times for the calculation. After each scan, they had to leave the scanner to repose for the next scan. All our operator staff passed the precision quality control required by ISCD, which controlled the CV% to less than 1.9% for lumbar spine scans, 1.8% for hip joint scans, and 2.5% for femoral neck scans (see Table 1); and (4) all participants were requested to remove outer garments and objects that would potentially interfere with testing. All DXA values were analyzed using Hologic Apex software version 4.0 following the manufacturer's guidelines.

--Table 1 about here--

Statistical analysis

The DXA measures for our pediatric participants were modeled against age or height as the independent variable. The LMS statistical method (LMS ChartMaker Pro Version 2.54)[30,31] using a curve fitting procedure was employed to construct reference smooth cross sectional

For peer review only - http://bmjopen.bmj.com/site/about/guidelines.xhtml

centile curves for BMD/body composition versus age or height. The LMS technique estimates three parameters: median (M), coefficient of variation (S), and power in the Box-Cox transformation (L). This summarizes the distribution of the dependent variable given the covariate by L depending on the skewness of the distribution, M and S giving smooth curves for the dependence of L, M, and S on the covariate. These three parameters vary as a function of independent variables fully describing the reference data. Once these parameters are estimated, then centile curves can be constructed using the formula that has been described elsewhere.[10,22] To avoid overfitting of the curves, parsimonious models were used instead of complex models when they had similar goodness of fit. After fitting the curves, the distribution of z-scores was observed and outlier analysis was performed to verify that all values were within physiological range (e.g., observations ≥ 3 SDs were removed). All the analyses were performed using SAS Version 9.4 (SAS Institute, Cary, NC).The z-scores were be calculated by the following equation:

$$Z=\frac{\left(\frac{X}{M}\right)^{L}-1}{LS}$$

If L=0,

$$Z=\ln(X/M)/S$$

where X is the TBLH BMD, L is the power transformation, M is the median value, and S is the population SD.[25] Percentiles were obtained from z-scores, e.g., z-scores of -1.645, -1.282, 0, 1.282, and 1.645 correspond to the 5th, 10th, 50th, 90th and 95th percentiles, respectively.

Results

Among 10,818 children residing in seven geographically distributed centers across China, children from Beijing accounted for the largest proportion of 16.2%, while children from Ningxia accounts for the smallest proportion of 12.2% (Table 2). In addition, the age- and sex specific TBLH BMD was significantly different among seven centers. Children residing in

For peer review only - http://bmjopen.bmj.com/site/about/guidelines.xhtml

Northeast China had the highest TBLH BMD while children residing in Shandong Province had the lowest value (Table 2).

--Table 2 about here--

TBLH BMD of children by sex and height is shown in Table 3. TBLH BMD increased with age for both sexes, e.g., from 0.412 g/cm^2 at age 3 to 0.948 g/cm^2 at age 18 for boys, and from 0.399 g/cm^2 to 0.853 g/cm^2 for girls. Similarly, TBLH BMD also increased with height for boys and girls. Overall, TBLH BMD was higher in boys than in girls.

--Table 3 about here--

The percentiles of TBLH BMD among Chinese and U.S. children were compared and are demonstrated in Figure 2. TBLH BMD increased with age among boys and girls in both countries; however, TBLH BMD was comparatively higher in children living in U.S. than those in China. Among boys, the differences at each percentile (i.e., P3, P50, and P97) between China and the U.S. were similar in general, which was small in young ages and peaked at age 18. BMD at P3, P50, and P97 was higher in American boys than in Chinese boys. There was a gradual increase in BMD among girls and boys after age 14 and age 16, respectively. Furthermore, BMD for age was higher in boys than in girls in both countries.

--Figure 2 about here--

In both China and the U.S., BMD increased with height in both sexes; U.S. children had a much higher BMD than Chinese children at each percentile, with the largest difference at P50 and P3 and the least difference at P97. The increasing rate of BMD for height was stable, with

For peer review only - http://bmjopen.bmj.com/site/about/guidelines.xhtml

an obvious increase from height 155 cm to 175 cm among U.S. children. Among girls with low heights (e.g., <135 cm), BMD value at P3 in U.S. children was similar to BMD value at P50 in Chinese children. A clear increase in BMD for height was observed from 150 cm to165 cm among U.S. girls as compared to increase in BMD from 145 to 155 cm among Chinese girls. At height greater than 155 cm, the difference in BMD became even larger. BMD peaked at 180 cm where BMD at P3 and P50 in the U.S. children were close to that at P50 and P97 of Chinese children. BMD for height was also higher in boys than in girls.

Discussion

In this study, we developed sex-specific reference standards using Hologic Discovery DXA scanner in a large nationwide sample of Chinese children aged 3-18 years. In addition, we compared the results from this study and references used in NHANES data, and found that BMD was lower in Chinese children than in U.S. children. To the best of our knowledge, this study is the first attempt to develop reference BMD values for Chinese children using a large nationwide pediatric population in China, and compare it with alternative available references.

China has a vast territory with large population. Disparities in their lifestyle behaviors, socioeconomic status, obesity and metabolic disorders may contribute to differences in BMD among children and adolescents. There is an urgent need to establish a unified national wide of BMD reference standards for children and adolescent, to help better evaluate their growth status. In addition, adequate peak bone mass at an early age was positively associated with lifelong health (e.g., osteoporosis at an older age);[32,33] therefore, an accurate assessment of BMD is essential in assisting development of future preventive methods to reduce the physical and economic burden of osteoporosis.

For peer review only - http://bmjopen.bmj.com/site/about/guidelines.xhtml

The difference in BMD among children may be due to their different dietary patterns. Compared to children consuming traditional Chinese diet (predominately consists of vegetables and grains, and features plant-based protein, high in fiber, and low in cholesterol and fat), children in western countries are more likely to consume foods that consists of more meats and milk, and thus is high in energy density, fat and protein.[34] Different genetic background should also be an explanation for the difference in BMD among children in China and U.S. There are no reference standards of BMD available for the Chinese pediatric population based on a large dataset. The present study utilized all body and regional DXA data for establishing age-, sex-, and height specific reference values for Chinese children aged 3-18 years, and provided centile curves of BMD, which can be used in both research and clinical settings.

DXA, a widely available technology, is capable of providing regional measures of fat and lean mass. The distribution of fat and lean mass is likely to predict health outcomes,[28] as shown previously in an adult sample.[31] DXA measurements provide precise body composition analysis with a low radiation dose,[35] and are able to detect small changes in body composition. DXA devices from different manufacturers might not give identical results due to differences in calibration and bone edge detection algorithms.[36] Therefore, to calculate reliable results one should use reference data obtained with an identical DXA device. For instance, U.S. NHANES data employed Hologic (Hologic, Inc, Bedford, MA) and GE-Lunar Inc. as the two dominant DXA manufacturers. Despite the similar DXA technology used for these manufacturers, the results of BMD and body composition can be different due to proprietary techniques used, algorithms to calculate the BMD, as well as the regions of interest, leading to various DXA values for patients who are scanned by different DXA systems.[8] To avoid the comparability problem, our study used Hologic as the only manufacturer. We used our collected data on BMD

For peer review only - http://bmjopen.bmj.com/site/about/guidelines.xhtml

percentiles among Chinese children to compare with those among U.S. children, where BMD values were obtained from U.S. NHANES data.[28]

Compared to our study, another study[37] conducted among southern Chinese pediatric population is different in selected geographic characteristics, sample size, and age range. For example, although 900 children (5-19 years) came from Guangzhou (Southern China) and about 600 (14-19 years) came from Zhejiang (East China), the study did not consider variation in geographic characteristics, and age range was smaller than ours (3-18 years). Equipment used in that study was GE Lunar, the results of which cannot be compared with ours using Hologic DXA.

Our study has strengths. First, we developed a number of reference curves generated from objective measures from DXA including sex- age specific and sex- height specific TBLH BMD, providing nationwide references for Chinese children. Second, the large sample size enabled us to construct stable reference curves and extended previous studies though covering a wide-range age from 3 to 18. Third, our study followed the recommendation of ISCD and studied TBLH BMD for Chinese children.

Several limitations were noteworthy. First, though our data is nationally and geographically distributed across China, it is not nationally representative and hence the results may not be generalizable to the entire Chinese pediatric population. For example, although Ningxia Province, representative of Northwest China, has thousands of Hui population, we mainly collected data from schools including Han population. Thus, our sampled participants across China likely only represent Chinese Han children. Moreover, our developed reference curve could not be used alone as clinical criteria for the diagnosis of osteoporosis. This is because 1) low bone mass may reflect only a small part of the true burden of osteoporosis, and 2) BMD could not demonstrate other important components of bone strength. Despite limitations,

For peer review only - http://bmjopen.bmj.com/site/about/guidelines.xhtml

there are some important public health implications. First, the BMD percentile can be used to precisely evaluate child and adolescent growth, and help high-risk population for better intervention. Second, since childhood obesity has been epidemic in the nation, assessing BMD can facilitate association studies between body composition (e.g., fat, muscle, etc.) and bone health, to better determine health status for obese individuals. Furthermore, it can be used for evaluating effectiveness of sports intervention among children. Clinical implications are also notable. First, child's kidney disease, rheumatism and immune disease, bone and joint disease can directly and indirectly influence bone growth; while BMD test can evaluate such diseases' adverse outcome on individual's health status and treatment effect. In addition, antiepileptic drugs and other hormonal drugs can affect osteogenesis, and BMD reference can be used as a standard for evaluation of treatment effect and side effects for children.

Conclusion

This is the first study to develop sex-specific references among 3-18-year-old Chinese children using the Hologic Discovery DXA scanner. The information can be used to guide clinicians improve interpretation, assessment, and monitoring of BMD results for Chinese children. However, it needs to be combined with other indicators (e.g., blood pressure, serum lipid, and calcium-phosphorus metabolism) to identify the diagnosis cut off points for osteopenia.

Acknowledgements

Author Contributions: Dr. Mi had full access to all the data in the study and takes responsibility for the integrity of the data and the accuracy of the data analysis. Study concept and design: Mi. Acquisition, analysis, or interpretation of data: Sun, Liu, Yan, Xi, Xiong, Ding, Jie Mi. Huang,

For peer review only - http://bmjopen.bmj.com/site/about/guidelines.xhtml

Mi. Drafting of the manuscript: Liu, Wang. Critical revision of the manuscript for important intellectual content: Liu, Wang, Heymsfield, Mi. Statistical analysis: Liu, Huang. Obtained funding: Mi. Administrative, technical, or material support: Sun, Liu, Yan, Xi, Xiong, Ding, Mi. Study supervision: Mi.

Conflict of Interest Disclosures: All authors declared no conflicts of interest.

Data sharing statement: Extra data is available by emailing Jiemi@vip.163.com.

Funding/support: The study was supported by grants from the "Twelfth Five-Year Plan" of the China National Science and Technology (2012BAI03B03), the Beijing Health System Leading Talent Grant (2009-1-08), the Beijing Training Project for the Leading Talents in Science and Technology (2011LJ07), and the content is solely the responsibility of the authors and does not necessarily represent the official views of the funders.

Role of the Funder/Sponsor: The funding sources had no role in the design and conduct of the study; collection, management, analysis, and interpretation of the data; preparation, review, or approval of the manuscript; and decision to submit the manuscript for publication.

For peer review only

References

1. Leslie WD, Morin SN: Osteoporosis epidemiology 2013: Implications for diagnosis, risk assessment and treatment. *Curr Opin Rheumatol*. 2014;26(4):440-446.

2. Liu ZH, Zhao YL, Ding GZ, Zhou Y. Epidemiology of primary osteoporosis in China. Osteoporos Int. 1997;7 Suppl 3:S84–S87.

3. Editorial Board of Osteoporosis Prevention and Treatment (China White Paper), China Health Promotion Foundation. White paper on osteoporosis. Chin J Health Manage. 2009. 3(3): 148-154.

4. Hansen MA, Overgaard K, Riis BJ, *et al*. Role of peak bone mass and bone loss in postmenopausal osteoporosis: 12 year study. BMJ. 1991;303:961-4.

5. Matkovic V, Jelic T, Wardlaw GM, et al. Timing of peak bone mass in caucasian females and its implication for the prevention of osteoporosis. Inference from a cross-sectional model. J Clin Invest 1994;93:799-808.

6. Ogle GD, Allen JR, Humphries IRJ, et al. Body-composition assessment by dual-energy x-ray absorptiometry in subjects aged 4-26 y. Am J Clin Nutr 1995;61:746-753.

7. Ward KA, Ashby RL, Roberts SA, Adams JE, Zulf Mughal M.UK reference data for the Hologic QDR Discovery dual-energy X ray absorptiometry scanner in healthy children and young adults aged 6-17 years. Arch Dis Child. 2007; 92(1):53-59.

8. Fan B, Shepherd JA, Levine MA, et al. National Health and Nutrition Examination Survey whole-body dual-energy X-ray absorptiometry reference data for GE Lunar systems. J Clin Densitom. 2014;17(3):344-377.

For peer review only - http://bmjopen.bmj.com/site/about/guidelines.xhtml

9. Genant HK, Engelke K, Fuerst T, Glüer CC, Grampp S, Harris ST, et al. Noninvasive assessment of bone mineral and structure: State of the art. J Bone Miner Res 11(6):707–30. 1996.

10. Lohman TG, Chen Z. Dual-energy x-ray absorptiometry. In: Heymsfield SB, Lohman T, Wang ZM, Going SB, eds. Human body composition, 2nd ed. Champaign, IL: Human Kinetics Press. 2005.

11. Njeh CF, Fuerst T, Hans D, Blake GM, Genant HK. Radiation exposure in bone mineral density assessment. Appl Radiat Isot.1999; 50(1):215–236.

12. Thomas SR, Kalkwarf HJ, Buckley DD, Heubi JE. Effective dose of dual-energy x-ray absorptiometry scans in children as a function of age. J Clin Densitom.2005; 8(4):415–422.

13. Wahner HW, Fogelman I. The evaluation of osteoporosis: Dual energy x-ray absorptiometry in clinical practice. London: Martin Dunitz Ltd. 1994.

14. WHO. Assessment of fracture risk and its application to screening for postmenopausal osteoporosis: report of a WHO study group. WHO Technical Report Series 843, WHO, Geneva 1994.

15. Uziel Y, Zifman E, & Hashkes PJ. (2009). Osteoporosis in children: pediatric and pediatric rheumatology perspective: a review. Pediatr Rheumatol Online J. 2009; 7:16.

16. Rizzoli R, Bianchi ML, Garabédian M, McKay HA, Moreno LA. Maximizing bone mineral mass gain during growth for the prevention of fractures in the adolescents and the elderly. Bone. 2010; 42(2): 294-305.

17. Moon RJ, Lim A, Farmer M, et al. Validity of parental recall of children's fracture: implications for investigation of childhood osteoporosis. Osteoporos Int. 2016; 27(2): 809–813.

For peer review only - http://bmjopen.bmj.com/site/about/guidelines.xhtml

18. Kinning E, McMillan M, Shepherd S, et al. An unbalanced rearrangement of chromosomes 4:20 is associated with childhood osteoporosis and reduced caspase-3 levels. J Pediatr Genet. 2016; 5(3): 167-173.

19. Bachrach LK, Hastie T, Wang M-C, et al. Bone mineral acquisition in healthy Asian, Hispanic, black, and Caucasian youth: a longitudinal study. J Clin Endocrinol Metab 1999;84:4702–4712.

20. Neu C, Manz F, Rauch F, et al. Bone densities and bone size at the distal radius in healthy children and adolescents: a study using peripheral quantitative computed tomography. Bone 2001; 28:227–232.

21. Schoenau E, Neu C, Rauch F, et al. Gender-specific pubertal changes in volumetric cortical bone mineral density at the proximal radius. Bone 2002; 31:110–113.

22. Nelson D, Simpson P, Johnson C, et al. The accumulation of whole body skeletal mass in third- and fourth-grade children: effects of age, gender, ethnicity and body composition. Bone 1997;20:73–78.

23. Wang MC, Aguirre M, Bhudhikanok GS, et al. Bone mass and hip axis length in healthy Asian, black, Hispanic, and white American youths. J Bone Miner Res 1997;12:1922–1935.

24. Bachrach LK, Gordon CM, SECTION ON ENDOCRINOLOGY. Bone densitometry in children and adolescents. Pediatrics. 2016; 138(4).

25. Crabtree NJ, Arabi A, Bachrach LK, et al; International Society for Clinical Densitometry. Dual-energy X-ray absorptiometry interpretation and reporting in children and adolescents: the revised 2013 ISCD Pediatric Official Positions. J Clin Densitom. 2014;17(2):225–242

26. Taylor A, Konrad PT, Norman ME, Harcke HT. Total body bone mineral density in young children: infl uence of head bone mineral density. J Bone Miner Res. 1997;12(4):652–655

For peer review only

27. NIH Consensus Development Panel on Osteoporosis Prevention, Diagnosis, and Therapy. Osteoporosis prevention, diagnosis, and therapy. JAMA. 2001; 285:785–795.

28. Kelly TL, Wilson KE, Heymsfield SB. Dual energy X-ray absorptiometry body composition reference values from NHANES. PLoS One. 2009; 4(9):e7038.

29. Lloyd-Jones DM, Hong Y, Labarthe D, et al. Defining and setting national goals for cardiovascular health promotion and disease reduction: the American Heart Association's strategic Impact Goal through 2020 and beyond. Circulation. 2010;121:586–613.

30. Cole TJ, Green PJ. Smoothing reference centile curves: the LMS method and penalized likelihood. Stat Med. 1992;11(10):1305-1309.

31. Pan H, Cole TJ. A comparison of goodness of fit tests for age-related reference ranges. Stat Med. 2004; 23(11):1749-1765.

32. Heaney RP, Abrams S, Dawson-Hughes B, Looker A, Marcus R, Matkovic V, Weaver C. Peak bone mass. Osteoporos Int. 2000;11:985–1009.

33. Rizzoli R, Bianchi ML, Garabedian M, McKay HA, Moreno LA. Maximizing bone mineral mass gain during growth for the prevention of fractures in the adolescents and the elderly. Bone. 2010; 46:294–305.

34. Xie QY. Cultural difference between the East and the West. Canadian Social Science 2007; 3(5): 114-117.

35. Mazess RB, Barden HS, Bisek JP, et al. Dual-energy absorptiometry for total body and regional bone mineral and soft tissue composition. Am J Clin Nutr 1990;51:1106–1112.

36. Bonnick SL. Bone densitometry in clinical practice. Totowa, New Jersey: Humana Press Inc., 1998.

For peer review only

37. Guo B, Xu Y, Gong J, Tang Y, Xu H. Age trends of bone mineral density and percentile curves in healthy Chinese children and adolescents. J Bone Miner Metab. 2013;31(3):304-314.

For peer review only

Figure Legend

Figure 1. Flow chart of the participants' recruitment, China Child and Adolescent Cardiovascular Health

Figure 2. The percentiles of TBLH BMD among Chinese and U.S. children by age and height.

TBLH BMD, Total body less head bone mineral density. US, Data from NHANES

CN, Data from China

For peer review only

For peer review only - http://bmjopen.bmj.com/site/about/guidelines.xhtml

Table 1. The distribution of coefficient of variation (CV, %) for lumbar spine, hip joint, and femoral neck by study location, the China Child and Adolescent Cardiovascular Health Study (n=10 818)

Province	Lumbar spine	Hip Joint	Femoral neck
Beijing	0.88	1.42	1.91
Shanghai	0.97	1.34	2.33
Tianjin	1.06	1.02	1.99
Chongqing	0.59	1.62	2.05
Jilin	1.58	1.79	1.39
Shandong	1.11	1.79	1.97
Ningxia	1.25	1.07	1.89

For peer review only

For peer review only - http://bmjopen.bmj.com/site/about/guidelines.xhtml

Table 2. Geographical distribution of sample, The China Child and Adolescent Cardiovascular Health (CCACH) (n=10 818)

Geographical area	Province	Latitude	N	%	TBLH BMD
Northeast	Jilin	N 40°52'~46°18'	1661	15.4	0.785±0.160
North China	Beijing	N39°26'~41°03'	1739	16.1	0.734±0.151
	Tianjin	N38°33'~40°15'	1653	15.3	
Northwest	Ningxia	N35°14'~39°23'	1315	12.2	0.743±0.129
East China	Shanghai,	N30°40'~31°53'	1613	14.9	0.740±0.131
	Shandong	N34°25'~38°23'	1437	13.3	
Southwest	Chongqing	N28°10'~32°13'	1400	12.9	0.778±0.143
F*					163.11
P					<0.0001

Abbreviations: TBLH, total body less head; BMD, bone mineral density.
*Analysis of covariance, adjusted for age and sex.

For peer review only - http://bmjopen.bmj.com/site/about/guidelines.xhtml

Table 3. TBLH BMD (g/cm^2) of children aged 3 to 18 years by sex and height, The China Child and Adolescent Cardiovascular Health (n=10 818)

Age,Height	Male							Female						
	Sample size	Mean±SD	TBLH BMD Percentiles					Sample size	Mean±SD	TBLH BMD Percentiles				
			5th	10th	50th	90th	95th			5th	10th	50th	90th	95th
Age(years)														
3	110	0.412±0.030	0.350	0.360	0.395	0.434	0.446	122	0.399±0.025	0.349	0.357	0.385	0.417	0.426
4	269	0.444±0.037	0.373	0.383	0.421	0.463	0.476	267	0.426±0.032	0.366	0.374	0.406	0.441	0.452
5	322	0.479±0.039	0.409	0.420	0.463	0.511	0.526	259	0.470±0.036	0.401	0.411	0.449	0.492	0.505
6	408	0.531±0.046	0.445	0.458	0.505	0.559	0.576	376	0.515±0.047	0.434	0.446	0.491	0.542	0.558
7	401	0.582±0.050	0.489	0.503	0.557	0.618	0.636	359	0.557±0.049	0.468	0.482	0.535	0.594	0.612
8	399	0.610±0.051	0.523	0.538	0.596	0.663	0.684	333	0.595±0.055	0.498	0.514	0.575	0.642	0.662
9	332	0.644±0.052	0.547	0.564	0.627	0.699	0.721	340	0.629±0.061	0.526	0.544	0.612	0.687	0.710
10	345	0.673±0.059	0.571	0.589	0.656	0.733	0.757	303	0.670±0.072	0.556	0.576	0.652	0.734	0.758
11	327	0.709±0.068	0.597	0.615	0.687	0.770	0.795	293	0.722±0.071	0.593	0.615	0.696	0.784	0.810
12	352	0.748±0.071	0.628	0.648	0.725	0.814	0.842	290	0.765±0.069	0.635	0.658	0.743	0.834	0.861
13	319	0.798±0.075	0.665	0.687	0.771	0.867	0.898	274	0.800±0.071	0.673	0.696	0.781	0.873	0.900
14	299	0.845±0.075	0.704	0.728	0.818	0.923	0.956	298	0.818±0.061	0.701	0.724	0.807	0.897	0.924
15	440	0.877±0.085	0.737	0.762	0.858	0.971	1.006	475	0.829±0.062	0.720	0.741	0.823	0.911	0.937
16	540	0.916±0.096	0.763	0.789	0.891	1.011	1.048	571	0.843±0.068	0.734	0.755	0.835	0.922	0.949
17	405	0.953±0.094	0.791	0.819	0.927	1.054	1.094	456	0.853±0.068	0.745	0.766	0.845	0.933	0.960
18	241	0.948±0.093	0.808	0.836	0.949	1.081	1.123	293	0.853±0.068	0.751	0.771	0.850	0.938	0.966
Height(cm)														
100	126	0.401±0.025	0.366	0.372	0.395	0.422	0.431	171	0.396±0.024	0.360	0.366	0.390	0.417	0.425
105	169	0.434±0.024	0.386	0.393	0.420	0.452	0.462	200	0.428±0.025	0.382	0.389	0.416	0.447	0.457
110	227	0.463±0.028	0.410	0.418	0.449	0.486	0.498	175	0.459±0.030	0.405	0.413	0.443	0.480	0.492
115	275	0.504±0.037	0.437	0.447	0.483	0.526	0.540	278	0.492±0.035	0.431	0.439	0.474	0.519	0.534
120	313	0.538±0.044	0.467	0.478	0.519	0.570	0.586	284	0.534±0.042	0.458	0.468	0.509	0.563	0.582
125	358	0.577±0.045	0.497	0.509	0.555	0.613	0.632	336	0.566±0.050	0.487	0.499	0.546	0.611	0.634
130	363	0.604±0.046	0.524	0.537	0.588	0.652	0.674	299	0.600±0.053	0.513	0.526	0.579	0.653	0.679
135	330	0.642±0.053	0.550	0.564	0.620	0.691	0.714	256	0.630±0.057	0.534	0.548	0.607	0.689	0.718
140	312	0.667±0.054	0.573	0.588	0.649	0.726	0.752	254	0.662±0.068	0.560	0.576	0.642	0.732	0.764
145	258	0.692±0.064	0.595	0.611	0.677	0.761	0.789	277	0.722±0.079	0.597	0.615	0.690	0.787	0.821
150	263	0.726±0.068	0.620	0.637	0.708	0.798	0.829	489	0.784±0.078	0.645	0.665	0.747	0.848	0.882
155	254	0.769±0.075	0.651	0.669	0.745	0.845	0.878	830	0.813±0.070	0.688	0.709	0.793	0.892	0.923
160	336	0.828±0.087	0.686	0.706	0.788	0.897	0.934	838	0.829±0.065	0.718	0.739	0.821	0.913	0.942
165	514	0.859±0.090	0.721	0.742	0.831	0.948	0.989	444	0.855±0.066	0.741	0.762	0.843	0.932	0.959

For peer review only - http://bmjopen.bmj.com/site/about/guidelines.xhtml

170	670	0.896±0.086	0.753	0.776	0.871	0.997	1.040	120	0.878±0.076	0.761	0.783	0.864	0.953	0.980
175	432	0.930±0.095	0.784	0.808	0.908	1.041	1.088	…	…	…	…	…	…	…
180	222	0.977±0.103	0.813	0.838	0.943	1.084	1.134	…	…	…	…	…	…	…

Abbreviations: TBLH, total body less head; BMD, bone mineral density; SD, standard deviation.

For peer review only

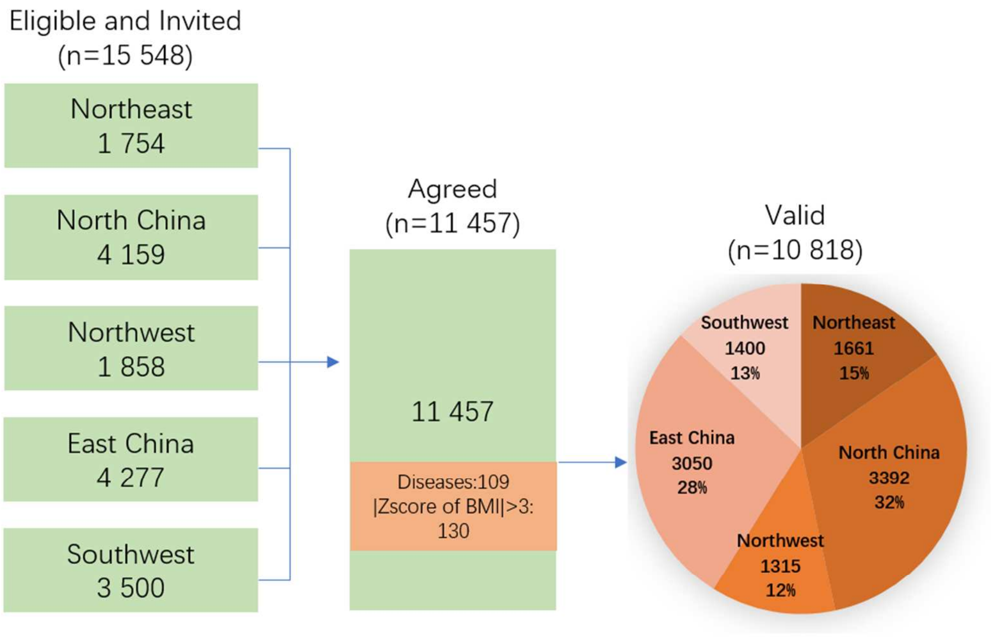

Figure1. Flow chart of the participants' recruitment, China Child and Adolescent Cardiovascular Health

86x58mm (300 x 300 DPI)

view only

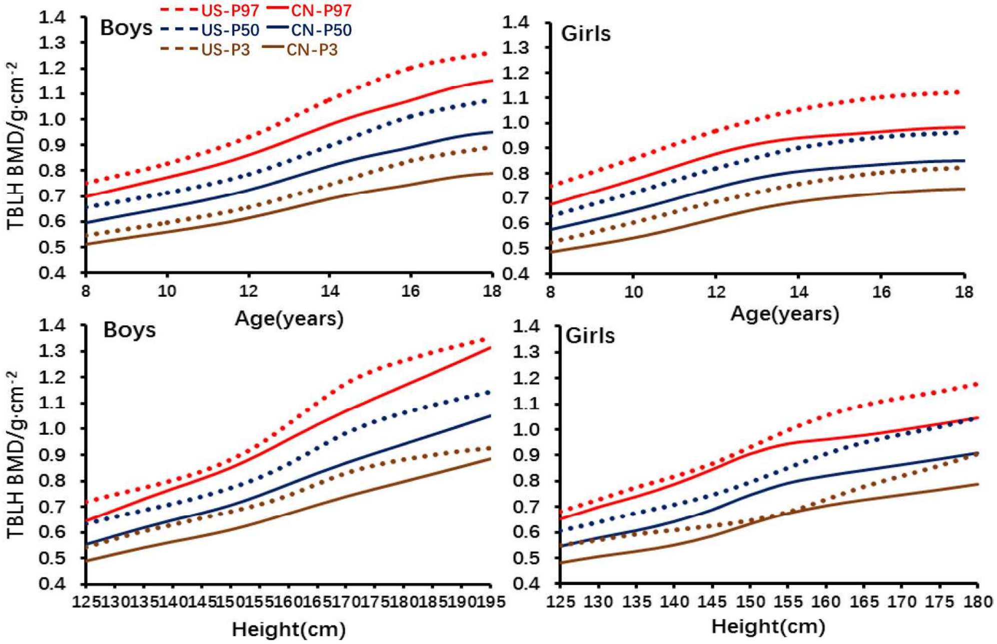

Fig2. The percentiles of TBLH BMD among Chinese and U.S. children by age and height.
TBLH BMD, Total body less head bone mineral density.US, Data from NHANES
CN, Data from China

217x163mm (300 x 300 DPI)

STROBE Statement—checklist of items that should be included in reports of observational studies

	Item No	Recommendation
**Title and abstract**	1	(*a*) Indicate the study's design with a commonly used term in the title or the abstract
Page 2		(*b*) Provide in the abstract an informative and balanced summary of what was done and what was found
**Introduction**		
Background/rationale Page 4	2	Explain the scientific background and rationale for the investigation being reported
Objectives Page 6	3	State specific objectives, including any prespecified hypotheses
**Methods**		
Study design Page 6	4	Present key elements of study design early in the paper
Setting Page 6	5	Describe the setting, locations, and relevant dates, including periods of recruitment, exposure, follow-up, and data collection
Participants Page 7	6	(*a*) *Cohort study*—Give the eligibility criteria, and the sources and methods of selection of participants. Describe methods of follow-up<br>*Case-control study*—Give the eligibility criteria, and the sources and methods of case ascertainment and control selection. Give the rationale for the choice of cases and controls<br>*Cross-sectional study*—Give the eligibility criteria, and the sources and methods of selection of participants
		(*b*) *Cohort study*—For matched studies, give matching criteria and number of exposed and unexposed<br>*Case-control study*—For matched studies, give matching criteria and the number of controls per case
Variables Page 7	7	Clearly define all outcomes, exposures, predictors, potential confounders, and effect modifiers. Give diagnostic criteria, if applicable
Data sources/ measurement Page 8	8*	For each variable of interest, give sources of data and details of methods of assessment (measurement). Describe comparability of assessment methods if there is more than one group
Bias Page 8	9	Describe any efforts to address potential sources of bias
Study size Page 9	10	Explain how the study size was arrived at
Quantitative variables Page 9	11	Explain how quantitative variables were handled in the analyses. If applicable, describe which groupings were chosen and why
Statistical methods Page 9	12	(*a*) Describe all statistical methods, including those used to control for confounding
		(*b*) Describe any methods used to examine subgroups and interactions
		(*c*) Explain how missing data were addressed
		(*d*) *Cohort study*—If applicable, explain how loss to follow-up was addressed<br>*Case-control study*—If applicable, explain how matching of cases and controls was addressed<br>*Cross-sectional study*—If applicable, describe analytical methods taking account of sampling strategy

For peer review only

For peer review only - http://bmjopen.bmj.com/site/about/guidelines.xhtml

		(*e*) Describe any sensitivity analyses
**Results**		
Participants<br>Page 9	13*	(a) Report numbers of individuals at each stage of study—eg numbers potentially eligible, examined for eligibility, confirmed eligible, included in the study, completing follow-up, and analysed
		(b) Give reasons for non-participation at each stage
		(c) Consider use of a flow diagram
Descriptive data<br>Page 10	14*	(a) Give characteristics of study participants (eg demographic, clinical, social) and information on exposures and potential confounders
		(b) Indicate number of participants with missing data for each variable of interest
		(c) *Cohort study*—Summarise follow-up time (eg, average and total amount)
Outcome data<br>Page 10	15*	*Cohort study*—Report numbers of outcome events or summary measures over time
		Case-control study—Report numbers in each exposure category, or summary measures of exposure
		Cross-sectional study—Report numbers of outcome events or summary measures
Main results<br>Page 10	16	(*a*) Give unadjusted estimates and, if applicable, confounder-adjusted estimates and their precision (eg, 95% confidence interval). Make clear which confounders were adjusted for and why they were included
		(*b*) Report category boundaries when continuous variables were categorized
		(*c*) If relevant, consider translating estimates of relative risk into absolute risk for a meaningful time period
Other analyses<br>Page 10	17	Report other analyses done—eg analyses of subgroups and interactions, and sensitivity analyses
**Discussion**		
Key results<br>Page 11	18	Summarise key results with reference to study objectives
Limitations<br>Page 13	19	Discuss limitations of the study, taking into account sources of potential bias or imprecision. Discuss both direction and magnitude of any potential bias
Interpretation<br>Page 13	20	Give a cautious overall interpretation of results considering objectives, limitations, multiplicity of analyses, results from similar studies, and other relevant evidence
Generalisability<br>Page 13	21	Discuss the generalisability (external validity) of the study results
**Other information**		
Funding<br>Page 15	22	Give the source of funding and the role of the funders for the present study and, if applicable, for the original study on which the present article is based

*Give information separately for cases and controls in case-control studies and, if applicable, for exposed and unexposed groups in cohort and cross-sectional studies.

Note: An Explanation and Elaboration article discusses each checklist item and gives methodological background and published examples of transparent reporting. The STROBE checklist is best used in conjunction with this article (freely available on the Web sites of PLoS Medicine at http://www.plosmedicine.org/, Annals of Internal Medicine at http://www.annals.org/, and Epidemiology at http://www.epidem.com/). Information on the STROBE Initiative is available at www.strobe-statement.org.

For peer review only

Supplemental Figure 1. Geographical distribution of sampled pediatric participants

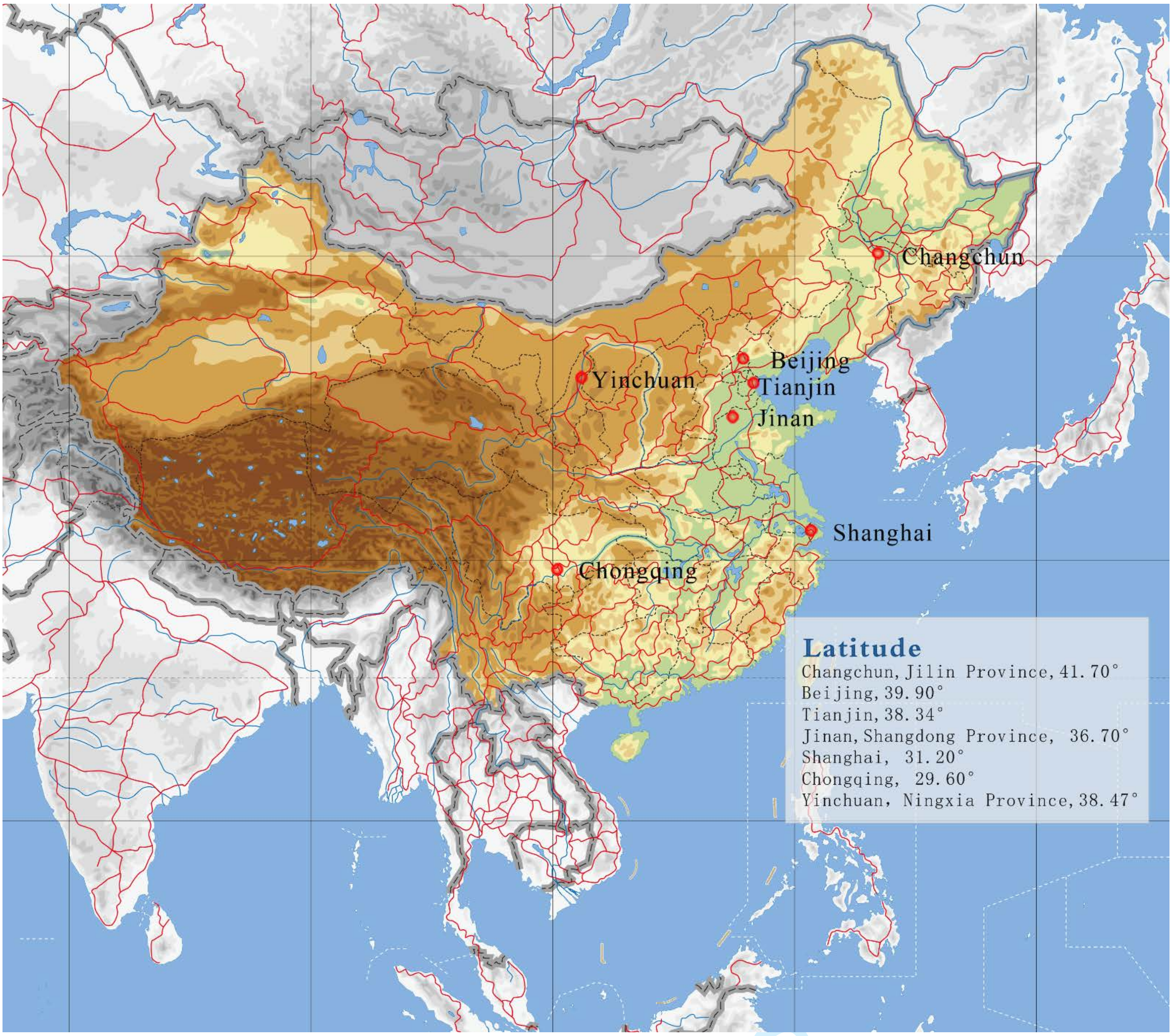

Supplemental Table 1. LMS parameters and reference centiles of TBLH BMD /g·cm^{-2} by age in boys

Age/yrs	L	S	Mean-2SD[a]	Mean-1SD[b]	Mean[c]	Mean+1SD[d]	Mean+2SD[e]	Percentiles					
								P3	P10	P25	P75	P90	P97
**3**	-0.251	0.073	0.342	0.367	0.395	0.425	0.458	0.345	0.360	0.376	0.415	0.434	0.454
**4**	-0.251	0.074	0.364	0.391	0.421	0.454	0.490	0.367	0.383	0.400	0.443	0.463	0.485
**5**	-0.251	0.076	0.399	0.429	0.463	0.500	0.541	0.402	0.420	0.440	0.487	0.511	0.536
**6**	-0.251	0.078	0.433	0.468	0.505	0.547	0.592	0.437	0.458	0.479	0.533	0.559	0.587
**7**	-0.251	0.080	0.476	0.515	0.557	0.604	0.655	0.480	0.503	0.528	0.588	0.618	0.649
**8**	-0.251	0.082	0.508	0.550	0.596	0.648	0.705	0.513	0.538	0.565	0.630	0.663	0.698
**9**	-0.251	0.084	0.532	0.577	0.627	0.682	0.743	0.537	0.564	0.593	0.663	0.699	0.736
**10**	-0.251	0.085	0.555	0.603	0.656	0.715	0.781	0.560	0.589	0.620	0.695	0.733	0.773
**11**	-0.251	0.087	0.579	0.630	0.687	0.751	0.821	0.585	0.615	0.648	0.729	0.770	0.813
**12**	-0.251	0.089	0.609	0.664	0.725	0.793	0.870	0.615	0.648	0.683	0.770	0.814	0.860
**13**	-0.251	0.091	0.645	0.704	0.771	0.845	0.928	0.652	0.687	0.725	0.820	0.867	0.918
**14**	-0.251	0.093	0.683	0.747	0.818	0.899	0.989	0.690	0.728	0.769	0.872	0.923	0.978
**15**	-0.251	0.095	0.713	0.782	0.858	0.944	1.042	0.721	0.762	0.806	0.915	0.971	1.030
**16**	-0.251	0.096	0.738	0.810	0.891	0.983	1.086	0.747	0.789	0.836	0.952	1.011	1.073
**17**	-0.251	0.098	0.765	0.841	0.927	1.024	1.134	0.774	0.819	0.868	0.991	1.054	1.120
**18**	-0.251	0.100	0.781	0.860	0.949	1.050	1.165	0.790	0.836	0.888	1.016	1.081	1.151

Abbreviations: TBLH BMD, total body less head bone mineral density; SD, standard deviation.

[a] Approximately 2.28th percent

[b] Approximately 15.87th percent

[c] Approximately 50th percent

[d] Approximately 84.13th percent

[e] Approximately 97.72th percent

For peer review only

Supplemental Table 2. LMS parameters and reference centiles of TBLH BMD /g·cm^{-2} by age in girls

Age/yrs	L	S	Mean-2SD[a]	Mean-1SD[b]	Mean[c]	Mean+1SD[d]	Mean+2SD[e]	Percentiles					
								P3	P10	P25	P75	P90	P97
**3**	-0.480	0.061	0.342	0.363	0.385	0.409	0.436	0.345	0.357	0.370	0.401	0.417	0.433
**4**	-0.417	0.064	0.358	0.381	0.406	0.433	0.463	0.361	0.374	0.389	0.424	0.441	0.459
**5**	-0.293	0.070	0.391	0.419	0.449	0.482	0.518	0.395	0.411	0.428	0.471	0.492	0.513
**6**	-0.166	0.076	0.423	0.456	0.491	0.531	0.573	0.427	0.446	0.467	0.517	0.542	0.568
**7**	-0.035	0.082	0.455	0.493	0.535	0.581	0.630	0.459	0.482	0.506	0.565	0.594	0.624
**8**	0.092	0.087	0.482	0.527	0.575	0.626	0.682	0.487	0.514	0.542	0.609	0.642	0.676
**9**	0.205	0.091	0.508	0.558	0.612	0.670	0.733	0.514	0.544	0.576	0.651	0.687	0.725
**10**	0.294	0.094	0.537	0.592	0.652	0.715	0.783	0.543	0.576	0.611	0.694	0.734	0.775
**11**	0.358	0.095	0.573	0.633	0.696	0.764	0.836	0.580	0.615	0.653	0.742	0.784	0.827
**12**	0.388	0.092	0.613	0.676	0.743	0.813	0.887	0.620	0.658	0.697	0.790	0.834	0.878
**13**	0.375	0.088	0.651	0.714	0.781	0.852	0.927	0.658	0.696	0.736	0.829	0.873	0.918
**14**	0.314	0.084	0.680	0.742	0.807	0.877	0.950	0.687	0.724	0.763	0.854	0.897	0.941
**15**	0.208	0.080	0.699	0.759	0.823	0.891	0.963	0.706	0.741	0.779	0.868	0.911	0.954
**16**	0.071	0.078	0.714	0.772	0.835	0.902	0.975	0.720	0.755	0.792	0.880	0.922	0.966
**17**	-0.087	0.077	0.725	0.783	0.845	0.913	0.987	0.732	0.766	0.802	0.890	0.933	0.978
**18**	-0.257	0.076	0.732	0.788	0.850	0.918	0.993	0.738	0.771	0.807	0.895	0.938	0.984

Abbreviations: TBLH BMD, total body less head bone mineral density; SD, standard deviation.

[a] Approximately 2.28th percent

[b] Approximately 15.87th percent

[c] Approximately 50th percent

[d] Approximately 84.13th percent

[e] Approximately 97.72th percent

For peer review only

Supplemental Table 3. LMS parameters and reference centiles of TBLH BMD /g·cm^{-2} by height/cm in boys

Height/cm	L	S	Mean-2SD[a]	Mean-1SD[b]	Mean[c]	Mean+1SD[d]	Mean+2SD[e]	Percentiles					
								P3	P10	P25	P75	P90	P97
95	-1.272	0.045	0.343	0.358	0.374	0.392	0.412	0.345	0.354	0.363	0.386	0.397	0.409
100	-1.272	0.050	0.360	0.377	0.395	0.416	0.439	0.362	0.372	0.382	0.409	0.422	0.436
105	-1.272	0.054	0.380	0.399	0.420	0.444	0.472	0.382	0.393	0.405	0.436	0.452	0.468
110	-1.272	0.059	0.403	0.424	0.449	0.477	0.510	0.405	0.418	0.432	0.468	0.486	0.506
115	-1.272	0.064	0.429	0.454	0.483	0.516	0.554	0.432	0.447	0.463	0.504	0.526	0.549
120	-1.272	0.068	0.458	0.486	0.519	0.558	0.603	0.461	0.478	0.496	0.544	0.570	0.597
125	-1.272	0.072	0.486	0.518	0.555	0.599	0.651	0.490	0.509	0.530	0.584	0.613	0.645
130	-1.272	0.076	0.512	0.547	0.588	0.637	0.696	0.516	0.537	0.560	0.620	0.652	0.688
135	-1.272	0.079	0.537	0.575	0.620	0.674	0.739	0.541	0.564	0.589	0.655	0.691	0.731
140	-1.272	0.082	0.560	0.601	0.649	0.708	0.780	0.564	0.588	0.615	0.687	0.726	0.770
145	-1.272	0.085	0.581	0.624	0.677	0.740	0.819	0.585	0.611	0.640	0.718	0.761	0.809
150	-1.272	0.087	0.604	0.651	0.708	0.776	0.862	0.609	0.637	0.668	0.752	0.798	0.851
155	-1.272	0.090	0.634	0.684	0.745	0.820	0.915	0.639	0.669	0.703	0.794	0.845	0.902
160	-1.272	0.093	0.667	0.722	0.788	0.870	0.974	0.673	0.706	0.742	0.841	0.897	0.960
165	-1.272	0.095	0.701	0.760	0.831	0.919	1.032	0.707	0.742	0.781	0.888	0.948	1.017
170	-1.272	0.097	0.733	0.795	0.871	0.965	1.087	0.739	0.776	0.818	0.932	0.997	1.071
175	-1.272	0.098	0.762	0.827	0.908	1.008	1.138	0.769	0.808	0.852	0.973	1.041	1.121
180	-1.272	0.100	0.790	0.859	0.943	1.049	1.187	0.797	0.838	0.884	1.012	1.084	1.169
185	-1.272	0.101	0.818	0.890	0.979	1.091	1.236	0.826	0.868	0.917	1.051	1.128	1.217
190	-1.272	0.102	0.847	0.922	1.015	1.133	1.286	0.855	0.899	0.950	1.091	1.171	1.265
195	-1.272	0.103	0.876	0.955	1.052	1.175	1.337	0.884	0.931	0.984	1.132	1.216	1.315

Abbreviations: TBLH BMD, total body less head bone mineral density; SD, standard deviation.

[a] Approximately 2.28th percent

[b] Approximately 15.87th percent

[c] Approximately 50th percent

[d] Approximately 84.13th percent

[e] Approximately 97.72th percent

For peer review only

Supplemental Table 4. LMS parameters and reference centiles of-TBLH BMD /g·cm^{-2} by height/cm in girls

Height/cm	L	S	Mean-2SD[a]	Mean-1SD[b]	Mean[c]	Mean+1SD[d]	Mean+2SD[e]	Percentiles					
								P3	P10	P25	P75	P90	P97
95	-0.926	0.047	0.339	0.354	0.371	0.389	0.409	0.340	0.350	0.359	0.383	0.395	0.407
100	-1.168	0.050	0.355	0.371	0.390	0.411	0.434	0.356	0.366	0.377	0.404	0.417	0.431
105	-1.413	0.054	0.376	0.395	0.416	0.439	0.467	0.378	0.389	0.401	0.431	0.447	0.463
110	-1.665	0.058	0.398	0.419	0.443	0.471	0.504	0.401	0.413	0.427	0.461	0.480	0.500
115	-1.871	0.064	0.423	0.446	0.474	0.508	0.550	0.425	0.439	0.455	0.496	0.519	0.544
120	-1.980	0.072	0.449	0.476	0.509	0.550	0.603	0.452	0.468	0.486	0.536	0.563	0.596
125	-2.008	0.078	0.477	0.508	0.546	0.595	0.659	0.480	0.499	0.520	0.578	0.611	0.650
130	-1.950	0.083	0.501	0.536	0.579	0.634	0.708	0.505	0.526	0.549	0.614	0.653	0.698
135	-1.776	0.088	0.521	0.560	0.607	0.668	0.750	0.525	0.548	0.574	0.647	0.689	0.739
140	-1.499	0.093	0.545	0.589	0.642	0.710	0.798	0.550	0.576	0.605	0.686	0.732	0.786
145	-1.146	0.096	0.580	0.630	0.690	0.763	0.856	0.585	0.615	0.648	0.737	0.787	0.844
150	-0.764	0.095	0.626	0.682	0.747	0.824	0.916	0.632	0.665	0.702	0.798	0.848	0.905
155	-0.417	0.089	0.668	0.727	0.793	0.869	0.954	0.674	0.709	0.747	0.843	0.892	0.944
160	-0.159	0.083	0.698	0.756	0.821	0.892	0.970	0.704	0.739	0.777	0.868	0.913	0.961
165	-0.004	0.078	0.721	0.779	0.843	0.912	0.986	0.727	0.762	0.800	0.889	0.932	0.977
170	0.096	0.077	0.741	0.800	0.864	0.933	1.007	0.747	0.783	0.821	0.910	0.953	0.998
175	0.187	0.076	0.760	0.822	0.887	0.956	1.030	0.767	0.804	0.842	0.933	0.976	1.021
180	0.281	0.075	0.781	0.844	0.910	0.980	1.054	0.788	0.826	0.865	0.957	1.000	1.045

Abbreviations: TBLH BMD, total body less head bone mineral density; SD, standard deviation.

[a] Approximately 2.28th percent

[b] Approximately 15.87th percent

[c] Approximately 50th percent

[d] Approximately 84.13th percent

[e] Approximately 97.72th percent

For peer review only

Supplemental Table 5. LMS parameters and reference centiles of FMP by age in boys

Age/yrs	L	S	Mean-2SD[a]	Mean-1SD[b]	Mean[c]	Mean+1SD[d]	Mean+2SD[e]	Percentiles					
								P3	P10	P25	P75	P90	P97
**3**	-0.651	0.101	26.1	28.6	31.5	35.0	39.1	26.3	27.8	29.5	33.8	36.1	38.6
**4**	-0.585	0.120	24.4	27.2	30.6	34.7	39.7	24.7	26.4	28.3	33.3	36.0	39.0
**5**	-0.483	0.151	22.2	25.4	29.4	34.3	40.7	22.5	24.4	26.6	32.6	36.0	39.8
**6**	-0.388	0.179	20.5	24.1	28.7	34.5	42.1	20.9	23.0	25.5	32.5	36.5	41.1
**7**	-0.301	0.204	19.4	23.3	28.4	35.1	43.9	19.8	22.1	24.9	32.7	37.3	42.7
**8**	-0.230	0.224	18.6	22.9	28.5	35.9	45.7	19.1	21.6	24.6	33.2	38.4	44.4
**9**	-0.184	0.240	18.1	22.7	28.7	36.6	47.4	18.6	21.2	24.4	33.8	39.4	45.9
**10**	-0.163	0.252	17.6	22.3	28.6	37.0	48.4	18.1	20.9	24.2	34.0	39.9	46.9
**11**	-0.168	0.261	17.0	21.7	28.0	36.6	48.4	17.5	20.3	23.6	33.5	39.6	46.8
**12**	-0.198	0.267	16.3	20.8	27.0	35.5	47.4	16.7	19.4	22.6	32.4	38.4	45.8
**13**	-0.250	0.269	15.5	19.8	25.6	33.9	45.7	15.9	18.4	21.5	30.9	36.8	44.1
**14**	-0.318	0.270	14.8	18.8	24.3	32.2	43.9	15.2	17.5	20.4	29.3	35.1	42.3
**15**	-0.397	0.269	14.3	18.0	23.2	30.9	42.5	14.7	16.8	19.5	28.1	33.7	40.9
**16**	-0.481	0.266	14.0	17.5	22.5	29.9	41.6	14.3	16.4	18.9	27.1	32.6	39.9
**17**	-0.564	0.264	13.8	17.1	21.9	29.1	40.9	14.1	16.1	18.5	26.4	31.8	39.2
**18**	-0.645	0.261	13.6	16.7	21.3	28.3	40.3	13.9	15.7	18.0	25.7	31.1	38.5

Abbreviations: FMP, fat mass percentage (%); SD, standard deviation.

[a] Approximately 2.28th percent

[b] Approximately 15.87th percent

[c] Approximately 50th percent

[d] Approximately 84.13th percent

[e] Approximately 97.72th percent

For peer review only

Supplemental Table 6. LMS parameters and reference centiles of FMP by age in girls

Age/yrs	L	S	Mean-2SD[a]	Mean-1SD[b]	Mean[c]	Mean+1SD[d]	Mean+2SD[e]	Percentiles					
								P3	P10	P25	P75	P90	P97
**3**	0.285	0.106	27.8	31.1	34.7	38.5	42.6	28.2	30.2	32.2	37.2	39.6	42.1
**4**	0.285	0.118	26.6	30.1	34.0	38.1	42.7	27.0	29.1	31.3	36.7	39.4	42.1
**5**	0.285	0.140	24.5	28.4	32.8	37.6	42.9	24.9	27.3	29.8	36.0	39.0	42.2
**6**	0.285	0.158	22.8	27.1	31.8	37.2	43.1	23.3	25.8	28.6	35.4	38.8	42.4
**7**	0.285	0.173	21.6	26.1	31.1	36.9	43.3	22.1	24.8	27.7	34.9	38.6	42.5
**8**	0.285	0.183	20.9	25.4	30.7	36.7	43.4	21.4	24.1	27.1	34.6	38.5	42.6
**9**	0.285	0.187	20.5	25.1	30.5	36.6	43.5	21.0	23.8	26.8	34.5	38.4	42.6
**10**	0.285	0.188	20.4	25.0	30.4	36.5	43.4	20.9	23.7	26.7	34.4	38.3	42.5
**11**	0.285	0.185	20.6	25.1	30.4	36.4	43.2	21.1	23.8	26.8	34.4	38.2	42.3
**12**	0.285	0.179	21.0	25.5	30.6	36.5	43.1	21.5	24.1	27.1	34.5	38.3	42.3
**13**	0.285	0.173	21.6	26.0	31.1	36.8	43.2	22.1	24.7	27.6	34.8	38.5	42.4
**14**	0.285	0.165	22.4	26.8	31.7	37.3	43.5	22.9	25.5	28.3	35.4	38.9	42.7
**15**	0.285	0.157	23.4	27.6	32.5	37.9	43.9	23.8	26.4	29.1	36.0	39.5	43.1
**16**	0.285	0.150	24.2	28.4	33.1	38.4	44.2	24.7	27.2	29.9	36.6	39.9	43.5
**17**	0.285	0.143	24.9	29.0	33.5	38.6	44.2	25.3	27.8	30.4	36.9	40.1	43.5
**18**	0.285	0.137	25.3	29.3	33.7	38.5	43.9	25.8	28.1	30.7	36.9	40.0	43.2

Abbreviations: FMP, fat mass percentage (%); SD, standard deviation.

[a] Approximately 2.28th percent

[b] Approximately 15.87th percent

[c] Approximately 50th percent

[d] Approximately 84.13th percent

[e] Approximately 97.72th percent

For peer review only

Supplemental Table 7. LMS parameters and reference centiles of FMI by age in boys

Age/yrs	L	S	Mean-2SD[a]	Mean-1SD[b]	Mean[c]	Mean+1SD[d]	Mean+2SD[e]	Percentiles					
								P3	P10	P25	P75	P90	P97
**3**	-0.682	0.133	3.93	4.41	5.01	5.76	6.72	3.98	4.27	4.59	5.50	6.00	6.59
**4**	-0.667	0.167	3.57	4.12	4.83	5.77	7.05	3.63	3.95	4.33	5.43	6.09	6.88
**5**	-0.645	0.220	3.13	3.75	4.61	5.84	7.72	3.19	3.56	4.00	5.38	6.29	7.45
**6**	-0.625	0.268	2.85	3.54	4.53	6.08	8.71	2.92	3.32	3.82	5.49	6.68	8.31
**7**	-0.608	0.311	2.70	3.44	4.57	6.45	9.99	2.77	3.20	3.75	5.72	7.22	9.43
**8**	-0.593	0.347	2.63	3.43	4.70	6.92	11.46	2.71	3.17	3.77	6.04	7.86	10.70
**9**	-0.578	0.373	2.62	3.47	4.86	7.40	12.91	2.70	3.19	3.84	6.38	8.51	11.97
**10**	-0.565	0.392	2.61	3.51	5.00	7.79	14.07	2.70	3.21	3.91	6.66	9.03	12.97
**11**	-0.553	0.404	2.59	3.51	5.05	7.97	14.70	2.68	3.20	3.92	6.78	9.29	13.52
**12**	-0.541	0.410	2.55	3.46	5.02	7.97	14.80	2.63	3.16	3.88	6.77	9.30	13.60
**13**	-0.530	0.412	2.49	3.40	4.93	7.84	14.52	2.58	3.09	3.80	6.65	9.14	13.34
**14**	-0.520	0.410	2.44	3.32	4.82	7.65	14.04	2.52	3.03	3.72	6.50	8.91	12.93
**15**	-0.510	0.407	2.40	3.27	4.73	7.47	13.53	2.48	2.98	3.66	6.36	8.68	12.48
**16**	-0.501	0.402	2.37	3.23	4.66	7.31	13.05	2.45	2.95	3.61	6.24	8.47	12.07
**17**	-0.492	0.398	2.35	3.20	4.60	7.16	12.61	2.43	2.92	3.58	6.13	8.27	11.69
**18**	-0.483	0.393	2.33	3.16	4.54	7.01	12.18	2.41	2.89	3.53	6.02	8.08	11.32

Abbreviations: FMI, fat mass index (Fat mass (kg)/height(m)^2) ; SD, standard deviation.

[a] Approximately 2.28th percent

[b] Approximately 15.87th percent

[c] Approximately 50th percent

[d] Approximately 84.13th percent

[e] Approximately 97.72th percent

For peer review only

Supplemental Table 8. LMS parameters for the reference centiles of FMI by age in girls

Age/yrs	L	S	Mean-2SD[a]	Mean-1SD[b]	Mean[c]	Mean+1SD[d]	Mean+2SD[e]	Percentiles					
								P3	P10	P25	P75	P90	P97
**3**	-0.731	0.146	4.11	4.66	5.36	6.25	7.45	4.17	4.49	4.87	5.94	6.55	7.28
**4**	-0.697	0.167	3.87	4.46	5.23	6.25	7.65	3.93	4.28	4.69	5.88	6.60	7.46
**5**	-0.635	0.206	3.47	4.13	5.01	6.25	8.08	3.54	3.92	4.38	5.79	6.69	7.82
**6**	-0.577	0.239	3.19	3.89	4.86	6.29	8.51	3.26	3.67	4.17	5.76	6.81	8.19
**7**	-0.523	0.266	3.00	3.74	4.79	6.38	8.94	3.07	3.50	4.04	5.79	6.98	8.57
**8**	-0.472	0.287	2.90	3.69	4.82	6.56	9.41	2.98	3.44	4.01	5.91	7.22	8.98
**9**	-0.423	0.300	2.90	3.73	4.95	6.82	9.89	2.98	3.47	4.08	6.12	7.53	9.44
**10**	-0.376	0.308	2.96	3.84	5.14	7.12	10.34	3.04	3.56	4.21	6.38	7.87	9.87
**11**	-0.331	0.309	3.06	4.00	5.38	7.45	10.74	3.16	3.70	4.39	6.67	8.22	10.26
**12**	-0.288	0.306	3.21	4.21	5.65	7.79	11.09	3.32	3.90	4.62	6.99	8.57	10.61
**13**	-0.246	0.299	3.40	4.46	5.95	8.11	11.35	3.51	4.12	4.88	7.31	8.89	10.89
**14**	-0.205	0.288	3.62	4.72	6.25	8.41	11.54	3.73	4.37	5.16	7.62	9.18	11.10
**15**	-0.165	0.276	3.85	4.99	6.53	8.66	11.64	3.97	4.63	5.44	7.89	9.40	11.23
**16**	-0.126	0.263	4.06	5.22	6.76	8.83	11.64	4.18	4.86	5.67	8.08	9.53	11.26
**17**	-0.088	0.250	4.24	5.40	6.91	8.90	11.52	4.36	5.04	5.84	8.19	9.56	11.17
**18**	-0.051	0.237	4.38	5.53	7.01	8.90	11.33	4.50	5.18	5.97	8.23	9.52	11.01

Abbreviations: FMI, fat mass index (Fat mass (kg)/height(m)^2) ; SD, standard deviation.

[a] Approximately 2.28th percent

[b] Approximately 15.87th percent

[c] Approximately 50th percent

[d] Approximately 84.13th percent

[e] Approximately 97.72th percent

For peer review only

Supplemental Table 9. LMS parameters and reference centiles of FFMI by age in boys

Age/yrs	L	S	Mean-2SD[a]	Mean-1SD[b]	Mean[c]	Mean+1SD[d]	Mean+2SD[e]	Percentiles					
								P3	P10	P25	P75	P90	P97
**3**	-0.656	0.058	9.58	10.12	10.71	11.36	12.07	9.64	9.96	10.30	11.14	11.55	11.99
**4**	-0.656	0.062	9.66	10.25	10.90	11.61	12.41	9.73	10.08	10.45	11.37	11.83	12.31
**5**	-0.656	0.070	9.80	10.46	11.20	12.04	12.98	9.87	10.27	10.69	11.76	12.29	12.87
**6**	-0.656	0.078	9.86	10.60	11.43	12.38	13.47	9.94	10.38	10.86	12.06	12.68	13.34
**7**	-0.656	0.085	9.92	10.73	11.66	12.73	13.98	10.01	10.49	11.02	12.37	13.06	13.82
**8**	-0.656	0.093	10.08	10.98	12.01	13.22	14.64	10.18	10.71	11.30	12.81	13.60	14.46
**9**	-0.656	0.100	10.22	11.20	12.34	13.70	15.31	10.33	10.91	11.55	13.23	14.12	15.10
**10**	-0.656	0.108	10.35	11.41	12.66	14.15	15.96	10.47	11.09	11.79	13.63	14.62	15.73
**11**	-0.656	0.114	10.61	11.75	13.12	14.76	16.78	10.74	11.41	12.17	14.19	15.29	16.52
**12**	-0.656	0.118	11.06	12.29	13.78	15.59	17.82	11.19	11.92	12.75	14.96	16.17	17.53
**13**	-0.656	0.121	11.66	12.99	14.60	16.56	19.00	11.81	12.59	13.48	15.88	17.19	18.68
**14**	-0.656	0.122	12.30	13.72	15.42	17.51	20.11	12.46	13.29	14.24	16.78	18.18	19.77
**15**	-0.656	0.121	12.78	14.24	16.00	18.16	20.84	12.94	13.80	14.78	17.41	18.85	20.49
**16**	-0.656	0.120	13.02	14.50	16.27	18.43	21.12	13.19	14.06	15.04	17.68	19.13	20.77
**17**	-0.656	0.118	13.19	14.66	16.43	18.57	21.22	13.35	14.22	15.20	17.83	19.26	20.88
**18**	-0.656	0.116	13.36	14.82	16.56	18.68	21.29	13.52	14.38	15.35	17.95	19.36	20.95

Abbreviations: FFMI, free fat mass index (Free fat mass (kg)/height(m)^2) ; SD, standard deviation.

[a] Approximately 2.28th percent

[b] Approximately 15.87th percent

[c] Approximately 50th percent

[d] Approximately 84.13th percent

[e] Approximately 97.72th percent

For peer review only

Supplemental Table 10. LMS parameters and reference centiles of FFMI by age in girls

Age/yrs	L	S	Mean-2SD[a]	Mean-1SD[b]	Mean[c]	Mean+1SD[d]	Mean+2SD[e]	Percentiles					
								P3	P10	P25	P75	P90	P97
**3**	-0.485	0.060	8.97	9.50	10.09	10.72	11.42	9.03	9.35	9.69	10.51	10.91	11.33
**4**	-0.484	0.064	9.00	9.57	10.19	10.87	11.62	9.06	9.40	9.76	10.64	11.07	11.53
**5**	-0.482	0.071	9.06	9.69	10.39	11.16	12.03	9.13	9.50	9.91	10.90	11.40	11.92
**6**	-0.480	0.078	9.11	9.81	10.59	11.46	12.44	9.19	9.60	10.05	11.16	11.72	12.32
**7**	-0.479	0.084	9.19	9.96	10.81	11.79	12.90	9.28	9.73	10.22	11.46	12.09	12.76
**8**	-0.477	0.091	9.34	10.17	11.11	12.19	13.43	9.43	9.92	10.46	11.82	12.52	13.28
**9**	-0.475	0.096	9.55	10.46	11.49	12.68	14.06	9.66	10.19	10.78	12.27	13.04	13.88
**10**	-0.474	0.101	9.84	10.81	11.93	13.22	14.74	9.94	10.52	11.15	12.78	13.63	14.55
**11**	-0.472	0.104	10.17	11.21	12.40	13.80	15.44	10.29	10.90	11.58	13.32	14.24	15.23
**12**	-0.470	0.106	10.51	11.60	12.87	14.34	16.08	10.63	11.28	11.99	13.84	14.81	15.86
**13**	-0.469	0.106	10.81	11.94	13.24	14.77	16.57	10.94	11.60	12.34	14.25	15.25	16.34
**14**	-0.467	0.106	11.05	12.19	13.51	15.06	16.88	11.17	11.85	12.60	14.53	15.54	16.65
**15**	-0.465	0.104	11.21	12.35	13.67	15.21	17.01	11.34	12.01	12.76	14.68	15.69	16.78
**16**	-0.463	0.102	11.31	12.44	13.74	15.25	17.01	11.44	12.11	12.84	14.73	15.72	16.79
**17**	-0.462	0.099	11.39	12.50	13.77	15.24	16.94	11.52	12.17	12.89	14.73	15.69	16.73
**18**	-0.460	0.096	11.48	12.56	13.80	15.22	16.87	11.60	12.24	12.94	14.74	15.66	16.66

Abbreviations: FFMI, free fat mass index (Free fat mass (kg)/height(m)^2) ; SD, standard deviation.

[a] Approximately 2.28th percent

[b] Approximately 15.87th percent

[c] Approximately 50th percent

[d] Approximately 84.13th percent

[e] Approximately 97.72th percent

For peer review only

BMJ Open

Bone mineral density reference standards for Chinese children aged 3-18: Cross-sectional results of the 2013-2015 China Child and Adolescent Cardiovascular Health (CCACH)

For peer review only

Journal:	*BMJ Open*
Manuscript ID	bmjopen-2016-014542.R2
Article Type:	Research
Date Submitted by the Author:	17-Mar-2017
Complete List of Authors:	Liu, Junting; Capital Institute of Pediatrics, Department of Epidemiology<br>Wang, Liang; East Tennessee State University, ; East Tennessee State University<br>Sun, Jinghui ; The First Hospital of Jilin University<br>Liu, Gongshu; Tianjin Women's and Children's Health Center,<br>Yan, Weili; Fudan Univ,<br>Xi, Bo; Shandong University<br>Xiong, Feng; Children's Hospital of Chongqing Medical University, Department of Endocrinology<br>Ding, Wenqing; Ningxia Medical University, Department of Public Health<br>Huang, Guimin; Capital Institute of Pediatrics, Department of Epidemiology<br>Heymsfield, Steven; Pennington Biomedical Research Center, Metabolism - Body Composition<br>Mi, Jie; Capital Institute of Pediatrics, Department of Epidemiology
**Primary Subject Heading**:	Epidemiology
Secondary Subject Heading:	Paediatrics, Public health
Keywords:	BMD, DXA, Children, China

SCHOLARONE™
Manuscripts

Bone mineral density reference standards for Chinese children aged 3-18: Cross-sectional results of the 2013-2015 China Child and Adolescent Cardiovascular Health (CCACH)

Junting Liu[1#], MS, Liang Wang[2#],MD, DrPH, MPH, Jinghui Sun, MD[3†],Gongshu Liu, MD [4†] ,Weili Yan, PhD[5†],Bo Xi[6†], PhD, Feng Xiong[7†], MS, Wenqing Ding[8†],PhD, Guimin Huang[1], MS, Steven B. Heymsfield, PhD,[9] Jie Mi[1*],MD,PhD[1]

[# †]These authors contributed equally to the work.

[1]Department of Epidemiology, Capital Institute of Pediatrics, Beijing 100020, China
[2]Department of Biostatistics and Epidemiology, College of Public Health, East Tennessee State University, TN 37614, USA
[3]Department of Cardiovascular Medicine, The First Hospital of Jilin University, Changchun 130021, China
[4]Project Office, Tianjin Women's and Children's Health Center, Tianjin 300070, China
[5]Department of Clinical Epidemiology, Children's Hospital of Fudan University, Shanghai 200433, China
[6]Department of Public Health, Shandong University, Jinan 250012, China
[7]Department of Endocrinology, Children's Hospital of Chongqing Medical University, Chongqing 400014, China
[8]Department of Public Health, Ningxia Medical University, Yinchuan 750003, China
[9]Department of Metabolism-Body Composition, Pennington Biomedical Research Center, Baton Rouge, LA 70808, USA

Email:
Junting Liu, Junting_liu@163.com; Liang Wang, wangl2@etsu.edu; Jinghui Sun, 13756661617@126.com; Gongshu Liu, Liugongshu727@163.com; Weili Yan, yanwl@fudan.edu.cn; Bo Xi, swei006@163.com; Feng Xiong, xiongfw2013@163.com; Wenqing Ding,13519598269@163.com; Guimin Huang, guiminhuang@163.com; Steven B. Heymsfield, steven.heymsfield@pbrc.edu; Jie Mi, Jiemi@vip.163.com

Corresponding author
Jie Mi, Department of Epidemiology, Capital Institute of Pediatrics, 2 Yabao Road, Chaoyang District, Beijing 100020, China. Tel: +86-10-85695591; Fax: +86-10-85632799; jiemi@vip.163.com.

Word count for main text: 3089

Number of table: 3

Number of figure: 2

Number of supplemental table: 10

Number of supplemental figure: 1

For peer review only - http://bmjopen.bmj.com/site/about/guidelines.xhtml

Abstract

Objectives: No nationwide pediatric reference standards for bone mineral density (BMD) are available in China. We aimed to provide sex-specific BMD reference values for Chinese children and adolescents (3-18 years).

Methods: Data (10,818 participants aged 3-18 years) were obtained from cross-sectional surveys of the China Child and Adolescent Cardiovascular Health (CCACH) in 2015, which included four municipality cities and three provinces. BMD was measured using Hologic Discovery Dual Energy X-ray Absorptiometry (DXA) scanner. The DXA measures were modeled against age, with height as an independent variable. The LMS statistical method using a curve fitting procedure was used to construct reference smooth cross sectional centile curves for dependent versus independent variables.

Results: Children residing in Northeast China had the highest total body less head (TBLH) BMD while children residing in Shandong Province had the lowest values. Among children, TBLH BMD was higher for boys as compared to girls; but, it increased with age and height in both sexes. Furthermore, TBLH BMD was higher among U.S. children as compared to Chinese children. There was a large difference in BMD for height among children from these two countries. U.S. children had a much higher BMD at each percentile (P) than Chinese children; the largest observed difference was at P50 and P3 and the smallest difference was at P97.

Conclusions: This is the first study to present a sex-specific reference dataset for 3-18 year old Chinese children. The data can help clinicians improve interpretation, assessment, and monitoring of densitometry results.

Key words: BMD, DXA, China, Children

For peer review only - http://bmjopen.bmj.com/site/about/guidelines.xhtml

Strengths and limitations of this study

- It is the first study to present a sex-specific reference dataset for 3-18 year old Chinese children.
- A number of reference curves were developed using objective measures from DXA.
- The large sample size enabled us to construct stable reference curves and extended previous studies though covering a wide-range of ages: from 3 to 18 years.
- The data we collected were not nationally representative; hence, we could not generalize the results to the entire Chinese pediatric population.
- Our developed reference curve could not be used alone as a clinical criteria for the diagnosis of pediatric osteoporosis.

For peer review only

For peer review only - http://bmjopen.bmj.com/site/about/guidelines.xhtml

Introduction

Osteoporosis, as a widespread metabolic bone disease, has become a global public health problem. More than 200 million people worldwide and 10 million people in the United States (U.S.) were afflicted with osteoporosis in 2013.[1] In China, the population with osteoporosis in 1997 was 83.9 million, which is projected to increase to 212 million by 2050.[2] The burdens associated with osteoporosis are heavy, with the most common being fractures. It has been estimated that the treatment cost for hip fracture, in China, would be more than 85 billion Chinese Yuan (i.e., about 13 billion U.S. dollars) in 2020, and 1800 billion Chinese Yuan (i.e., about 271 U.S. dollars) in 2050.[3]

Peak bone mass is an important determinant for osteoporotic fracture risk. A higher peak bone mass is associated with lower risk of osteoporotic fractures later in life.[4] In general, individuals have peak bone mass in their early 20s; however, it may occur earlier in women.[5,6] Thus, investigating the pattern of bone mineralization in children is of great interest. Dual-energy X-ray absorptiometry (DXA) has been recommended by the International Society for Clinical Densitometry (ISCD)[7] as a current criterion for measuring body mineral content (BMC) and bone mineral density (BMD). This measurement method has high precision, accuracy, speed, low radiation exposure, ease of use, and a strong correlation with established methods.[8-14]

Pediatric osteoporosis is defined as having a history of pathologic fractures and low bone mineral content or density.[15] To diagnose pediatric osteoporosis, DXA is the most widely used bone densitometry technique in children.[7] Although 60% of peak bone mass is genetically determined, the increase of bone mass can be influenced by other factors, including intake of dietary products (e.g., calcium and vitamin D) and weight-bearing physical activity (e.g., jumping).[16]

For peer review only - http://bmjopen.bmj.com/site/about/guidelines.xhtml

While diagnosing children can be problematic due to the lack of a widely used reference, the Bone Mineral Density in Childhood Study (BMDCS) has developed an online BMD z-score calculator for health care providers (https://bmdcs.nichd.nih.gov/zscore.htm). Widespread reference for multiple diagnostic measurements is important to ensure correct diagnosis among children so that treatment and management for osteoporosis can begin. Uniformity in diagnosis is also imperative for public health so that epidemiologic studies can be done across geographic regions, ethnicities, and social economic status to properly inform prevention practices.

For children, DXA can be used to measure whole body bone area (cm^2) and whole body BMC (g), leading to BMD (i.e., BMC divided by bone area (g/cm^2)) which indicates the pediatric bone status. The use and correct identification of indicators for BMD is particularly important; this requires appropriate reference data that are matched for multiple factors including sex,[17-19] chronological age, height, weight, pubertal development and ethnicity.[17,20-22] The preferred skeletal sites are lumbar spine (L1–4) and total body, excluding the head.[23] The cranium should not be included for the total body scan analysis, because the head constitutes a large portion of the total body bone mass but changes little with growth, activity, or disease. Thus, including the skull is likely to mask gains or losses at other skeletal sites.[24]

Childhood and adolescence are critical periods for bone growth, with half of peak bone mass developed in childhood. If BMD could be evaluated in childhood and adolescence, and children with low BMD could be intervened upon, then peak bone mass can be improved in adulthood. Subsequently, osteoporosis occurrence can be delayed, and severity of osteoporosis can be reduced. A number of studies have shown that optimal BMC and BMD in childhood and adolescence would reduce risk of osteoporotic fracture several decades later.[25] To date, no pediatric reference standards for BMD are available in China. Furthermore, utilization of U.S.

For peer review only - http://bmjopen.bmj.com/site/about/guidelines.xhtml

measures based on reference values derived from the U.S. NHANES data might not be applicable for Chinese pediatric populations. Therefore, it is critically important and urgent to develop reference values of BMD for Chinese children based upon data gathered from Chinese children. These reference standards can help clinicians have a better estimation of the percentile on which Chinese children's DXA-measured bone parameter falls in reference to their age or height. The aim of this study was twofold: 1) to develop sex-specific reference percentile curves for Chinese children based on a large, nationwide sample; and 2) to compare our findings with those of the study conducted by Kelly and colleagues[26] using U.S. NHANES data.

Methods

Participants

The American Heart Association defined a new concept of "ideal cardiovascular health (CVH)" for American adults and children in 2010,[27] which included four health behaviors (nonsmoking, physically active, normal body mass index (BMI), healthy diet) and three health factors (normal blood pressure, total cholesterol, fasting glucose). Based on these seven health behaviors and factors, the construct of CVH comprehensively combines the traditional risk factors proposed by the Framingham Heart Study, as well as lifestyle behaviors, emphasizing the importance of overall strategies in achieving optimal CV health. Our data were obtained from the China Child and Adolescent Cardiovascular Health Study (CCACHs). It is a large, nationwide, ongoing study designed to examine CVH, BMD, body composition, and daily sodium intake among Chinese children ages 3-18 (Figure 1). A stratified cluster random sampling method was used to extract 100 children per age group in each cluster (50 boys and 50 girls). Because the majority of children are Han, accounting for 90% of the total Chinese pediatric population,

For peer review only - http://bmjopen.bmj.com/site/about/guidelines.xhtml

CCACHs mainly collected CVH data among Han children. Children were recruited from seven provinces (centers) covering geographical areas in North, East, Northeast, Northwest, and Southwest regions of China, across latitude 29.6° north to latitude 41.7° north (Supplement Figure 1). Eight exclusion criteria were: (1) the inability to give informed consent, (2) any condition, or use of any drug known to affect bone health, (3) non-removable objects (e.g., prostheses, implants, or casts), (4) body weight greater than 204 kg, and height greater than 197.5 cm, or weight or height beyond the measurement range of machines, (5) participation in research involving ionizing radiation in the past year, (6) no history of bone fracture, (7) some chronic diseases, and (8) pregnancy. Among the 15,548 eligible participants, 11,057 agreed to participate and signed written informed consent documents. Data were collected on 11,457 children; of these 400 Hui children were excluded, 109 were excluded due to kidney or thyroid disease, and 130 were excluded because of implausible values of weight (light or heavy, BMI ≥ 3 SDs). Consequently, 10,818 children (5,509 males and 5,309 females) were used in the final analysis.

The study was approved by the Research Ethics Committee of Capital Institute of Pediatrics of China. The written informed consent documents for children were obtained from their parents or guardians.

--Figure 1 about here--

DXA acquisition and quality control

The whole-body scans were performed using Hologic Discovery (A, W, and Wi) fan-beam densitometers (Hologic, Inc., Bedford, Massachusetts, USA). The coefficient of variation (CV%) was used as the quality control procedure. CV% of A, W and Wi was 0.471%, 0.302% and 0.358%, respectively. Following the recommendations of the ISCD, a high level of quality

For peer review only - http://bmjopen.bmj.com/site/about/guidelines.xhtml

control was maintained throughout the DXA data collection and scan analysis. The specific procedures were as follows: (1) Standard phantom was checked before scanning the participants and calibrating of the DXA machines every morning; (2) an operator at each study location was trained and the same technician certified by ISCD administered DXA procedures. The training materials included the ISCD's official technician hands-on training materials and the manufacturer's handbook including testing procedures and operation methods; (3) at each center, lumbar spine and hip joints of 15 participants were scanned 3 times for the calculation. After each scan, they had to leave the scanner to repose before the next scan. All our operator staff passed the precision quality control required by ISCD, which controlled the CV% to less than 1.9% for lumbar spine scans, 1.8% for hip joint scans, and 2.5% for femoral neck scans (see Table 1); and (4) all participants were requested to remove outer garments and objects that would potentially interfere with testing. All DXA values were analyzed using Hologic Apex software version 4.0 following the manufacturer's guidelines.

--Table 1 about here--

Statistical analysis

The DXA measures for our pediatric participants were modeled against age or height as the independent variable. The LMS statistical method (LMS ChartMaker Pro Version 2.54)[28,29] using a curve fitting procedure was employed to construct reference smooth cross sectional centile curves for BMD/body composition versus age or height. The LMS technique estimates three parameters: median (M), coefficient of variation (S), and power in the Box-Cox transformation (L). This summarizes the distribution of the dependent variable given the covariate by L depending on the skewness of the distribution while M and S gives smooth curves

For peer review only - http://bmjopen.bmj.com/site/about/guidelines.xhtml

for the dependence of L, M, and S on the covariate. These three parameters vary as a function of independent variables fully describing the reference data. Once these parameters are estimated, then centile curves can be constructed using the formula that has been described elsewhere.[10,20] To avoid overfitting of the curves, parsimonious models were used instead of complex models when they had similar goodness of fit. After fitting the curves, the distribution of z-scores was observed and outlier analysis was performed to verify that all values were within physiological range (e.g., observations ≥ 3 SDs were removed). All the analyses were performed using SAS Version 9.4 (SAS Institute, Cary, NC).The z-scores were be calculated by the following equation:

$$Z=\frac{\left(\frac{X}{M}\right)^{L}-1}{LS}$$

If L=0,

$$Z=\ln(X/M)/S$$

Where X is the TBLH BMD, L is the power transformation, M is the median value, and S is the population SD.[23] Percentiles were obtained from z-scores, e.g., z-scores of -1.645, -1.282, 0, 1.282, and 1.645 correspond to the 5th, 10th, 50th, 90th and 95th percentiles, respectively.

Results

Among the 10,818 children residing in seven geographically distributed centers across China, children from Beijing accounted for the largest proportion (16.2%), while children from Ningxia accounts for the smallest proportion (12.2%) (Table 2). In addition, the age- and sex specific TBLH BMD was significantly different among seven centers. Children residing in Northeast China had the highest TBLH BMD while children residing in Shandong Province had the lowest value (Table 2).

--Table 2 about here--

For peer review only - http://bmjopen.bmj.com/site/about/guidelines.xhtml

TBLH BMD of children by sex and height is shown in Table 3. TBLH BMD increased with age for both sexes, e.g., from 0.412 g/cm^2 at age 3 to 0.948 g/cm^2 at age 18 for boys, and from 0.399 g/cm^2 to 0.853 g/cm^2 for girls. Similarly, TBLH BMD also increased with height for boys and girls. Overall, TBLH BMD was higher in boys than in girls.

--Table 3 about here--

The percentiles of TBLH BMD among Chinese and U.S. children were compared and are shown in Figure 2. TBLH BMD increased with age among boys and girls in both countries; however, TBLH BMD was comparatively higher in children living in the U.S. than those in China. Among boys, the differences at each percentile (i.e., P3, P50, and P97) between China and the U.S. were similar, in general; the differences were small in young ages and peaked at age 18. BMD at P3, P50, and P97 was higher in American boys than in Chinese boys. There was a gradual increase in BMD among girls and boys after age 14 and age 16, respectively. Furthermore, BMD for age was higher in boys than in girls in both countries.

--Figure 2 about here--

In both China and the U.S., BMD increased with height in both sexes; U.S. children had a much higher BMD than Chinese children at each percentile, with the largest difference at P50 and P3 and the least difference at P97. The increasing rate of BMD for height was stable, with an obvious increase from height 155 cm to 175 cm among U.S. children. Among girls with low heights (e.g., <135 cm), BMD value at P3 in U.S. children was similar to BMD value at P50 in Chinese children. A clear increase in BMD for height was observed from 150 cm to165 cm among U.S. girls as compared to increase in BMD from 145 to 155 cm among Chinese girls. At

For peer review only - http://bmjopen.bmj.com/site/about/guidelines.xhtml

height greater than 155 cm, the difference in BMD became even larger. BMD peaked at 180 cm where BMD at P3 and P50 in the U.S. children were close to those at P50 and P97 in Chinese children. BMD for height was also higher in boys than in girls.

Discussion

In this study, we developed sex-specific reference standards using Hologic Discovery DXA scanner in a large nationwide sample of Chinese children aged 3-18 years. In addition, we compared the results from this study and references used in NHANES data, and found that BMD was lower in Chinese children than in U.S. children. To the best of our knowledge, this study is the first attempt to develop reference BMD values for Chinese children using a large nationwide pediatric population in China, and compare it with alternative available references.

China has a vast territory with large populations. Disparities in lifestyle behaviors, socioeconomic status, obesity and metabolic disorders may contribute to differences in BMD among children and adolescents. There is an urgent need to establish unified, nationwide BMD reference standards for children and adolescents, to help better evaluate their growth status. In addition, adequate peak bone mass at an early age was positively associated with lifelong health (e.g., osteoporosis at an older age);[30,31] therefore, an accurate assessment of BMD is essential in assisting development of future preventive methods to reduce the physical and economic burden of osteoporosis.

The difference in BMD among children may be due to their different dietary patterns. Compared to children consuming traditional Chinese diet (predominately consisting of vegetables and grains, and featuring plant-based protein which ishigh in fiber, and low in cholesterol and fat), children in western countries are more likely to consume foods that consists

For peer review only - http://bmjopen.bmj.com/site/about/guidelines.xhtml

of more meats and milk. Thus their diet is high in energy density, fat and protein.[32] Different genetic backgrounds should also be an explanation for the difference in BMD among children in China and U.S. There are no reference standards of BMD available for the Chinese pediatric populations based on a large dataset. The present study utilized all body and regional DXA data to establish age-, sex-, and height specific reference values for Chinese children aged 3-18 years, and provided centile curves of BMD, which can be used in both research and clinical settings.

DXA, a widely available technology, is capable of providing regional measures of fat and lean mass. The distribution of fat and lean mass is likely to predict health outcomes,[26] as shown previously in an adult sample.[29] DXA measurements provide precise body composition analysis with a low radiation dose,[33] and are able to detect small changes in body composition. DXA devices from different manufacturers might not give identical results due to differences in calibration and bone edge detection algorithms.[34] Therefore, to calculate reliable results one should use reference data obtained with an identical DXA device. For instance, U.S. NHANES data employed Hologic (Hologic, Inc, Bedford, MA) and GE-Lunar Inc. as the two dominant DXA manufacturers. Despite the similar DXA technology used for by manufacturers, the results of BMD and body composition can be different due to proprietary techniques used, algorithms to calculate the BMD, as well as the regions of interest. This leads to various DXA values for patients who are scanned by different DXA systems.[8] To avoid the comparability problem, our study used Hologic as the only manufacturer. We used our collected data on BMD percentiles among Chinese children to compare with those among U.S. children (BMD values were obtained from U.S. NHANES data).[26]

Compared to our study, another study[35] conducted among southern Chinese pediatric populations is different in selected geographic characteristics, sample size, and age range. For

For peer review only - http://bmjopen.bmj.com/site/about/guidelines.xhtml

example, although 900 children (5-19 years) came from Guangzhou (Southern China) and about 600 (14-19 years) came from Zhejiang (East China), the study did not consider variation in geographic characteristics; and their age range was smaller than ours (3-18 years). Equipment used in that study was GE Lunar, therefore their results cannot be compared with ours, having used Hologic DXA.

Our study has many strengths. First, we developed a number of reference curves generated from objective measures from DXA including sex-, age-, and sex- height specific TBLH BMD, providing nationwide references for Chinese children. Second, the large sample size enabled us to construct stable reference curves and extended previous studies though covering a wide-range of ages from 3 to 18 years. Third, our study followed the recommendation of ISCD and studied TBLH BMD for Chinese children.

Several limitations are noteworthy. First, though our data is nationally and geographically distributed across China, it is not nationally representative; hence, the results may not be generalizable to the entire Chinese pediatric population. For example, although Ningxia Province, representative of Northwest China, has thousands of Hui population, we mainly collected data from schools including Han population. Thus, our sampled participants across China likely only represent Chinese Han children. Moreover, our developed reference curve could not be used alone as a clinical criteria for the diagnosis of osteoporosis. This is because 1) low bone mass may reflect only a small part of the true burden of osteoporosis, and 2) BMD could not demonstrate other important components of bone strength. Despite these limitations, there are some important public health implications. First, the BMD percentile can be used to precisely evaluate child and adolescent growth, and help high-risk populations become connected with better interventions. Second, since childhood obesity has been epidemic in the nation, assessing

For peer review only - http://bmjopen.bmj.com/site/about/guidelines.xhtml

BMD can facilitate association studies between body composition (e.g., fat, muscle, etc.) and bone health, to better determine health status for obese individuals. Furthermore, it can be used for evaluating effectiveness of sports intervention among children. Clinical implications are also notable. First, childhood kidney disease, rheumatism and immune diseases, and bone and joint diseases can directly and indirectly influence bone growth; BMD tests can evaluate these diseases' adverse outcomes on an individual's health status and treatment effects. In addition, antiepileptic drugs and other hormonal drugs can affect osteogenesis, and BMD reference can be used as a standard for evaluation of treatment effects and side effects for children.

Conclusion

This is the first study to develop sex-specific references among 3-18-year-old Chinese children using the Hologic Discovery DXA scanner. The information can be used to guide clinicians and improve interpretation, assessment, and monitoring of BMD results for Chinese children. However, it needs to be combined with other indicators (e.g., blood pressure, serum lipid, and calcium-phosphorus metabolism) to identify the diagnosis cut points for osteopenia.

Acknowledgements

Author Contributions: Dr. Mi had full access to all the data in the study and takes responsibility for the integrity of the data and the accuracy of the data analysis. Study concept and design: Mi. Acquisition, analysis, or interpretation of data: Sun, Liu, Yan, Xi, Xiong, Ding, Jie Mi. Huang, Mi. Drafting of the manuscript: Liu, Wang. Critical revision of the manuscript for important intellectual content: Liu, Wang, Heymsfield, Mi. Statistical analysis: Liu, Huang. Obtained funding: Mi. Administrative, technical, or material support: Sun, Liu, Yan, Xi, Xiong, Ding, Mi.

For peer review only - http://bmjopen.bmj.com/site/about/guidelines.xhtml

Study supervision: Mi.

Conflict of Interest Disclosures: All authors declared no conflicts of interest.

Data sharing statement: Extra data is available by emailing Jiemi@vip.163.com.

Funding/support: The study was supported by grants from the "Twelfth Five-Year Plan" of the China National Science and Technology (2012BAI03B03), the Beijing Health System Leading Talent Grant (2009-1-08), the Beijing Training Project for the Leading Talents in Science and Technology (2011LJ07), and the content is solely the responsibility of the authors and does not necessarily represent the official views of the funders.

Role of the Funder/Sponsor: The funding sources had no role in the design and conduct of the study; collection, management, analysis, and interpretation of the data; preparation, review, or approval of the manuscript; and decision to submit the manuscript for publication.

For peer review only

References

1. Leslie WD, Morin SN: Osteoporosis epidemiology 2013: Implications for diagnosis, risk assessment and treatment. *Curr Opin Rheumatol*. 2014;26(4):440-446.

2. Liu ZH, Zhao YL, Ding GZ, Zhou Y. Epidemiology of primary osteoporosis in China. Osteoporos Int. 1997;7 Suppl 3:S84–S87.

3. Editorial Board of Osteoporosis Prevention and Treatment (China White Paper), China Health Promotion Foundation. White paper on osteoporosis. Chin J Health Manage. 2009. 3(3): 148-154.

4. Hansen MA, Overgaard K, Riis BJ, *et al*. Role of peak bone mass and bone loss in postmenopausal osteoporosis: 12 year study. BMJ. 1991;303:961-4.

5. Matkovic V, Jelic T, Wardlaw GM, et al. Timing of peak bone mass in caucasian females and its implication for the prevention of osteoporosis. Inference from a cross-sectional model. J Clin Invest 1994;93:799-808.

6. Ogle GD, Allen JR, Humphries IRJ, et al. Body-composition assessment by dual-energy x-ray absorptiometry in subjects aged 4-26 y. Am J Clin Nutr 1995;61:746-753.

7. Ward KA, Ashby RL, Roberts SA, Adams JE, Zulf Mughal M.UK reference data for the Hologic QDR Discovery dual-energy X ray absorptiometry scanner in healthy children and young adults aged 6-17 years. Arch Dis Child. 2007; 92(1):53-59.

8. Fan B, Shepherd JA, Levine MA, et al. National Health and Nutrition Examination Survey whole-body dual-energy X-ray absorptiometry reference data for GE Lunar systems. J Clin Densitom. 2014;17(3):344-377.

For peer review only

9. Genant HK, Engelke K, Fuerst T, Glüer CC, Grampp S, Harris ST, et al. Noninvasive assessment of bone mineral and structure: State of the art. J Bone Miner Res 11(6):707–30. 1996.

10. Lohman TG, Chen Z. Dual-energy x-ray absorptiometry. In: Heymsfield SB, Lohman T, Wang ZM, Going SB, eds. Human body composition, 2nd ed. Champaign, IL: Human Kinetics Press. 2005.

11. Njeh CF, Fuerst T, Hans D, Blake GM, Genant HK. Radiation exposure in bone mineral density assessment. Appl Radiat Isot.1999; 50(1):215–236.

12. Thomas SR, Kalkwarf HJ, Buckley DD, Heubi JE. Effective dose of dual-energy x-ray absorptiometry scans in children as a function of age. J Clin Densitom.2005; 8(4):415–422.

13. Wahner HW, Fogelman I. The evaluation of osteoporosis: Dual energy x-ray absorptiometry in clinical practice. London: Martin Dunitz Ltd. 1994.

14. WHO. Assessment of fracture risk and its application to screening for postmenopausal osteoporosis: report of a WHO study group. WHO Technical Report Series 843, WHO, Geneva 1994.

15. Uziel Y, Zifman E, & Hashkes PJ. (2009). Osteoporosis in children: pediatric and pediatric rheumatology perspective: a review. Pediatr Rheumatol Online J. 2009; 7:16.

16. Rizzoli R, Bianchi ML, Garabédian M, McKay HA, Moreno LA. Maximizing bone mineral mass gain during growth for the prevention of fractures in the adolescents and the elderly. Bone. 2010; 42(2): 294-305.

17. Bachrach LK, Hastie T, Wang M-C, et al. Bone mineral acquisition in healthy Asian, Hispanic, black, and Caucasian youth: a longitudinal study. J Clin Endocrinol Metab 1999;84:4702–4712.

For peer review only

For peer review only - http://bmjopen.bmj.com/site/about/guidelines.xhtml

18. Neu C, Manz F, Rauch F, et al. Bone densities and bone size at the distal radius in healthy children and adolescents: a study using peripheral quantitative computed tomography. Bone 2001; 28:227–232.

19. Schoenau E, Neu C, Rauch F, et al. Gender-specific pubertal changes in volumetric cortical bone mineral density at the proximal radius. Bone 2002; 31:110–113.

20. Nelson D, Simpson P, Johnson C, et al. The accumulation of whole body skeletal mass in third- and fourth-grade children: effects of age, gender, ethnicity and body composition. Bone 1997;20:73–78.

21. Wang MC, Aguirre M, Bhudhikanok GS, et al. Bone mass and hip axis length in healthy Asian, black, Hispanic, and white American youths. J Bone Miner Res 1997;12:1922–1935.

22. Bachrach LK, Gordon CM, SECTION ON ENDOCRINOLOGY. Bone densitometry in children and adolescents. Pediatrics. 2016; 138(4).

23. Crabtree NJ, Arabi A, Bachrach LK, et al; International Society for Clinical Densitometry. Dual-energy X-ray absorptiometry interpretation and reporting in children and adolescents: the revised 2013 ISCD Pediatric Official Positions. J Clin Densitom. 2014;17(2):225–242

24. Taylor A, Konrad PT, Norman ME, Harcke HT. Total body bone mineral density in young children: infl uence of head bone mineral density. J Bone Miner Res. 1997;12(4):652–655

25. NIH Consensus Development Panel on Osteoporosis Prevention, Diagnosis, and Therapy. Osteoporosis prevention, diagnosis, and therapy. JAMA. 2001; 285:785–795.

26. Kelly TL, Wilson KE, Heymsfield SB. Dual energy X-ray absorptiometry body composition reference values from NHANES. PLoS One. 2009; 4(9):e7038.

For peer review only - http://bmjopen.bmj.com/site/about/guidelines.xhtml

27. Lloyd-Jones DM, Hong Y, Labarthe D, et al. Defining and setting national goals for cardiovascular health promotion and disease reduction: the American Heart Association's strategic Impact Goal through 2020 and beyond. Circulation. 2010;121:586–613.

28. Cole TJ, Green PJ. Smoothing reference centile curves: the LMS method and penalized likelihood. Stat Med. 1992;11(10):1305-1309.

29. Pan H, Cole TJ. A comparison of goodness of fit tests for age-related reference ranges. Stat Med. 2004; 23(11):1749-1765.

30. Heaney RP, Abrams S, Dawson-Hughes B, Looker A, Marcus R, Matkovic V, Weaver C. Peak bone mass. Osteoporos Int. 2000;11:985–1009.

31. Rizzoli R, Bianchi ML, Garabedian M, McKay HA, Moreno LA. Maximizing bone mineral mass gain during growth for the prevention of fractures in the adolescents and the elderly. Bone. 2010; 46:294–305.

32. Xie QY. Cultural difference between the East and the West. Canadian Social Science 2007; 3(5): 114-117.

33. Mazess RB, Barden HS, Bisek JP, et al. Dual-energy absorptiometry for total body and regional bone mineral and soft tissue composition. Am J Clin Nutr 1990;51:1106–1112.

34. Bonnick SL. Bone densitometry in clinical practice. Totowa, New Jersey: Humana Press Inc., 1998.

35. Guo B, Xu Y, Gong J, Tang Y, Xu H. Age trends of bone mineral density and percentile curves in healthy Chinese children and adolescents. J Bone Miner Metab. 2013;31(3):304-314.

For peer review only - http://bmjopen.bmj.com/site/about/guidelines.xhtml

Figure Legend

Figure 1. Flow chart of the participants' recruitment, China Child and Adolescent Cardiovascular Health

Figure 2. The percentiles of TBLH BMD among Chinese and U.S. children by age and height.

TBLH BMD, Total body less head bone mineral density. US, Data from NHANES

CN, Data from China

For peer review only

For peer review only - http://bmjopen.bmj.com/site/about/guidelines.xhtml

Table 1. The distribution of coefficient of variation (CV, %) for lumbar spine, hip joint, and femoral neck by study location, the China Child and Adolescent Cardiovascular Health Study (n=10 818)

Province	Lumbar spine	Hip Joint	Femoral neck
Beijing	0.88	1.42	1.91
Shanghai	0.97	1.34	2.33
Tianjin	1.06	1.02	1.99
Chongqing	0.59	1.62	2.05
Jilin	1.58	1.79	1.39
Shandong	1.11	1.79	1.97
Ningxia	1.25	1.07	1.89

For peer review only

For peer review only - http://bmjopen.bmj.com/site/about/guidelines.xhtml

Table 2. Geographical distribution of sample, The China Child and Adolescent Cardiovascular Health (CCACH) (n=10 818)

Geographical area	Province	Latitude	N	%	TBLH BMD
Northeast	Jilin	N 40°52'~46°18'	1661	15.4	0.785±0.160
North China	Beijing	N39°26'~41°03'	1739	16.1	0.734±0.151
	Tianjin	N38°33'~40°15'	1653	15.3	
Northwest	Ningxia	N35°14'~39°23'	1315	12.2	0.743±0.129
East China	Shanghai,	N30°40'~31°53'	1613	14.9	0.740±0.131
	Shandong	N34°25'~38°23'	1437	13.3	
Southwest	Chongqing	N28°10'~32°13'	1400	12.9	0.778±0.143
F*					163.11
P					<0.0001

Abbreviations: TBLH, total body less head; BMD, bone mineral density.
*Analysis of covariance, adjusted for age and sex.

For peer review only - http://bmjopen.bmj.com/site/about/guidelines.xhtml

Table 3. TBLH BMD (g/cm^2) of children aged 3 to 18 years by sex and height, The China Child and Adolescent Cardiovascular Health (n=10 818)

Age,Height	Male Sample size	Male Mean±SD	Male TBLH BMD Percentiles 5th	10th	50th	90th	95th	Female Sample size	Female Mean±SD	Female TBLH BMD Percentiles 5th	10th	50th	90th	95th
Age(years)														
3	110	0.412±0.030	0.350	0.360	0.395	0.434	0.446	122	0.399±0.025	0.349	0.357	0.385	0.417	0.426
4	269	0.444±0.037	0.373	0.383	0.421	0.463	0.476	267	0.426±0.032	0.366	0.374	0.406	0.441	0.452
5	322	0.479±0.039	0.409	0.420	0.463	0.511	0.526	259	0.470±0.036	0.401	0.411	0.449	0.492	0.505
6	408	0.531±0.046	0.445	0.458	0.505	0.559	0.576	376	0.515±0.047	0.434	0.446	0.491	0.542	0.558
7	401	0.582±0.050	0.489	0.503	0.557	0.618	0.636	359	0.557±0.049	0.468	0.482	0.535	0.594	0.612
8	399	0.610±0.051	0.523	0.538	0.596	0.663	0.684	333	0.595±0.055	0.498	0.514	0.575	0.642	0.662
9	332	0.644±0.052	0.547	0.564	0.627	0.699	0.721	340	0.629±0.061	0.526	0.544	0.612	0.687	0.710
10	345	0.673±0.059	0.571	0.589	0.656	0.733	0.757	303	0.670±0.072	0.556	0.576	0.652	0.734	0.758
11	327	0.709±0.068	0.597	0.615	0.687	0.770	0.795	293	0.722±0.071	0.593	0.615	0.696	0.784	0.810
12	352	0.748±0.071	0.628	0.648	0.725	0.814	0.842	290	0.765±0.069	0.635	0.658	0.743	0.834	0.861
13	319	0.798±0.075	0.665	0.687	0.771	0.867	0.898	274	0.800±0.071	0.673	0.696	0.781	0.873	0.900
14	299	0.845±0.075	0.704	0.728	0.818	0.923	0.956	298	0.818±0.061	0.701	0.724	0.807	0.897	0.924
15	440	0.877±0.085	0.737	0.762	0.858	0.971	1.006	475	0.829±0.062	0.720	0.741	0.823	0.911	0.937
16	540	0.916±0.096	0.763	0.789	0.891	1.011	1.048	571	0.843±0.068	0.734	0.755	0.835	0.922	0.949
17	405	0.953±0.094	0.791	0.819	0.927	1.054	1.094	456	0.853±0.068	0.745	0.766	0.845	0.933	0.960
18	241	0.948±0.093	0.808	0.836	0.949	1.081	1.123	293	0.853±0.068	0.751	0.771	0.850	0.938	0.966
Height(cm)														
100	126	0.401±0.025	0.366	0.372	0.395	0.422	0.431	171	0.396±0.024	0.360	0.366	0.390	0.417	0.425
105	169	0.434±0.024	0.386	0.393	0.420	0.452	0.462	200	0.428±0.025	0.382	0.389	0.416	0.447	0.457
110	227	0.463±0.028	0.410	0.418	0.449	0.486	0.498	175	0.459±0.030	0.405	0.413	0.443	0.480	0.492
115	275	0.504±0.037	0.437	0.447	0.483	0.526	0.540	278	0.492±0.035	0.431	0.439	0.474	0.519	0.534
120	313	0.538±0.044	0.467	0.478	0.519	0.570	0.586	284	0.534±0.042	0.458	0.468	0.509	0.563	0.582
125	358	0.577±0.045	0.497	0.509	0.555	0.613	0.632	336	0.566±0.050	0.487	0.499	0.546	0.611	0.634
130	363	0.604±0.046	0.524	0.537	0.588	0.652	0.674	299	0.600±0.053	0.513	0.526	0.579	0.653	0.679
135	330	0.642±0.053	0.550	0.564	0.620	0.691	0.714	256	0.630±0.057	0.534	0.548	0.607	0.689	0.718
140	312	0.667±0.054	0.573	0.588	0.649	0.726	0.752	254	0.662±0.068	0.560	0.576	0.642	0.732	0.764
145	258	0.692±0.064	0.595	0.611	0.677	0.761	0.789	277	0.722±0.079	0.597	0.615	0.690	0.787	0.821
150	263	0.726±0.068	0.620	0.637	0.708	0.798	0.829	489	0.784±0.078	0.645	0.665	0.747	0.848	0.882
155	254	0.769±0.075	0.651	0.669	0.745	0.845	0.878	830	0.813±0.070	0.688	0.709	0.793	0.892	0.923
160	336	0.828±0.087	0.686	0.706	0.788	0.897	0.934	838	0.829±0.065	0.718	0.739	0.821	0.913	0.942
165	514	0.859±0.090	0.721	0.742	0.831	0.948	0.989	444	0.855±0.066	0.741	0.762	0.843	0.932	0.959

For peer review only - http://bmjopen.bmj.com/site/about/guidelines.xhtml

170	670	0.896±0.086	0.753	0.776	0.871	0.997	1.040	120	0.878±0.076	0.761	0.783	0.864	0.953	0.980
175	432	0.930±0.095	0.784	0.808	0.908	1.041	1.088	…	…	…	…	…	…	…
180	222	0.977±0.103	0.813	0.838	0.943	1.084	1.134	…	…	…	…	…	…	…

Abbreviations: TBLH, total body less head; BMD, bone mineral density; SD, standard deviation.

For peer review only

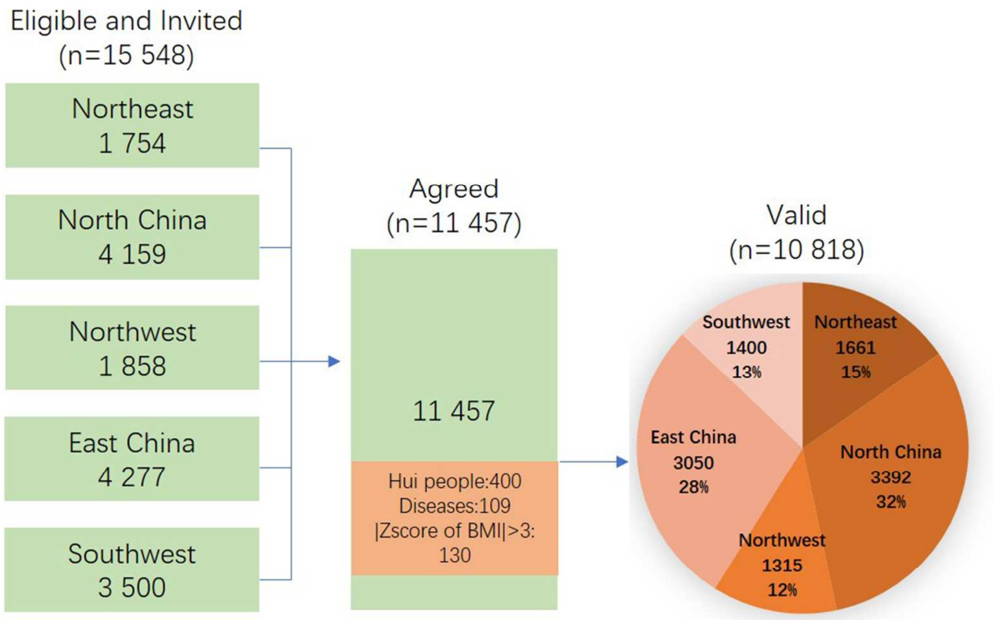

Figure1. Flow chart of the participants' recruitment, China Child and Adolescent Cardiovascular Health

86x57mm (300 x 300 DPI)

view only

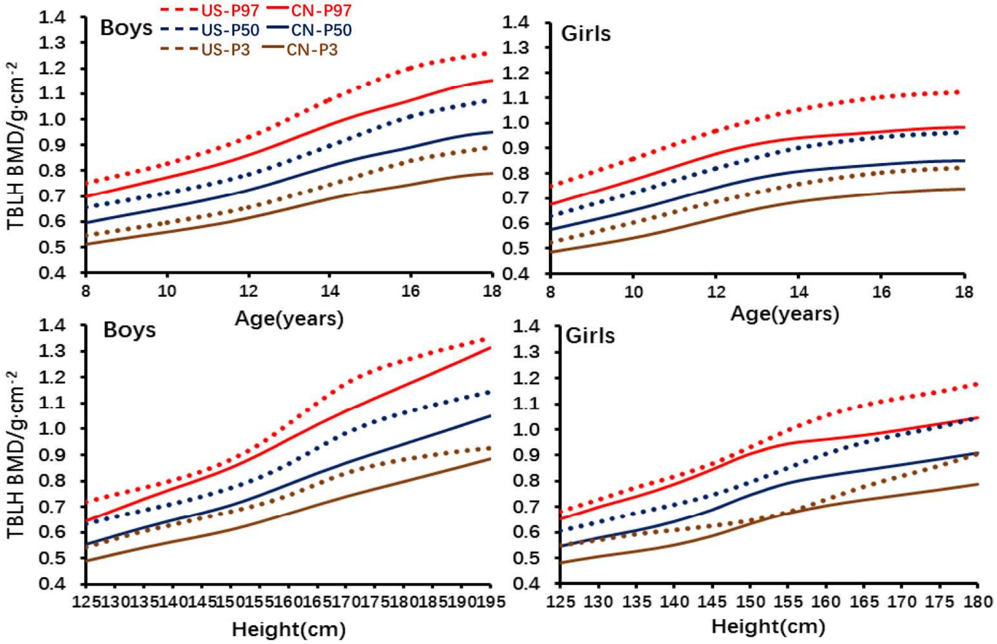

Fig2. The percentiles of TBLH BMD among Chinese and U.S. children by age and height. TBLH BMD, Total body less head bone mineral density.US, Data from NHANES CN, Data from China

217x163mm (300 x 300 DPI)

Supplemental Figure 1. Geographical distribution of sampled pediatric participants

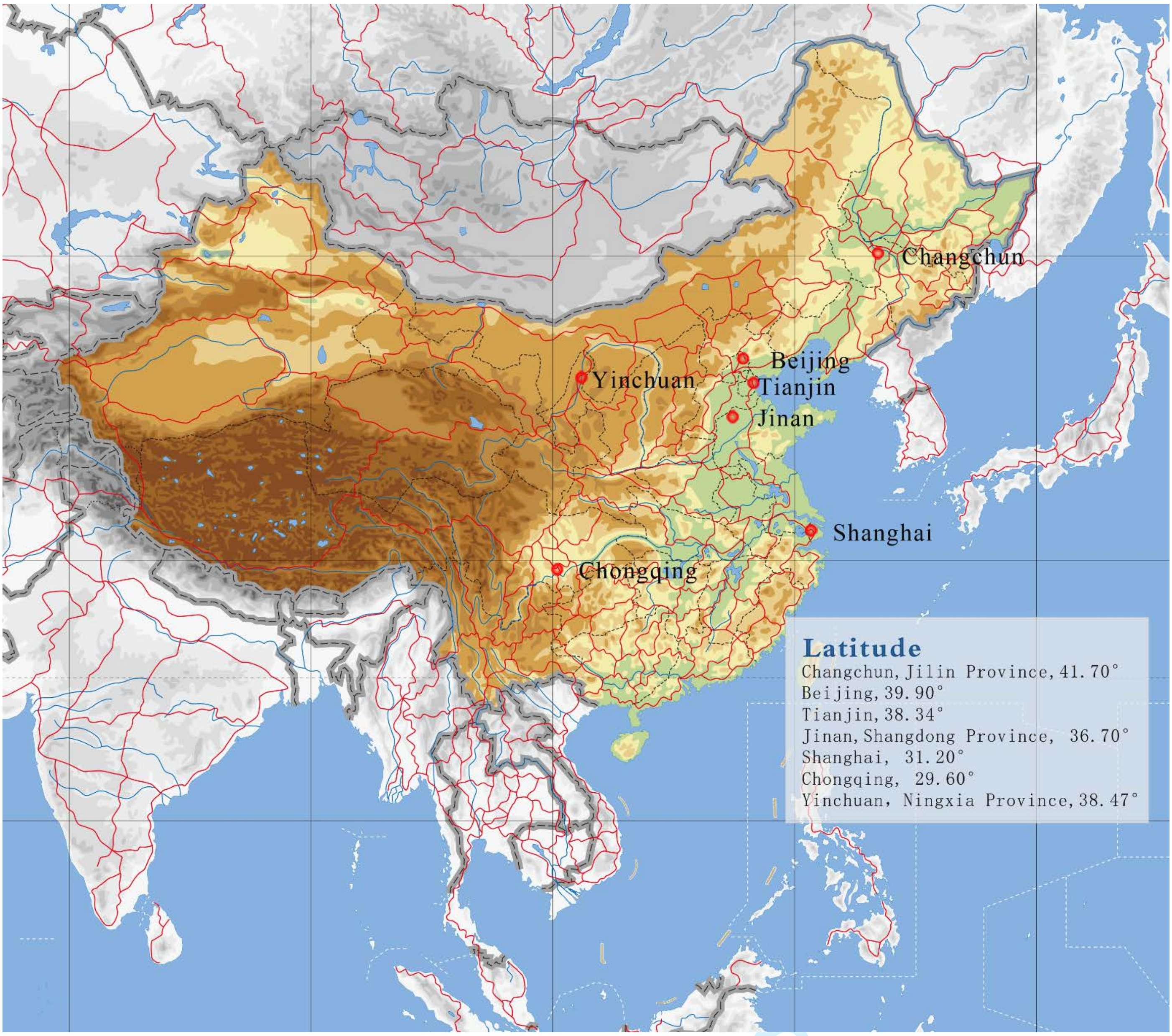

Supplemental Table 1. LMS parameters and reference centiles of TBLH BMD /g·cm^{-2} by age in boys

Age/yrs	L	S	Mean-2SD[a]	Mean-1SD[b]	Mean[c]	Mean+1SD[d]	Mean+2SD[e]	Percentiles					
								P3	P10	P25	P75	P90	P97
**3**	-0.251	0.073	0.342	0.367	0.395	0.425	0.458	0.345	0.360	0.376	0.415	0.434	0.454
**4**	-0.251	0.074	0.364	0.391	0.421	0.454	0.490	0.367	0.383	0.400	0.443	0.463	0.485
**5**	-0.251	0.076	0.399	0.429	0.463	0.500	0.541	0.402	0.420	0.440	0.487	0.511	0.536
**6**	-0.251	0.078	0.433	0.468	0.505	0.547	0.592	0.437	0.458	0.479	0.533	0.559	0.587
**7**	-0.251	0.080	0.476	0.515	0.557	0.604	0.655	0.480	0.503	0.528	0.588	0.618	0.649
**8**	-0.251	0.082	0.508	0.550	0.596	0.648	0.705	0.513	0.538	0.565	0.630	0.663	0.698
**9**	-0.251	0.084	0.532	0.577	0.627	0.682	0.743	0.537	0.564	0.593	0.663	0.699	0.736
**10**	-0.251	0.085	0.555	0.603	0.656	0.715	0.781	0.560	0.589	0.620	0.695	0.733	0.773
**11**	-0.251	0.087	0.579	0.630	0.687	0.751	0.821	0.585	0.615	0.648	0.729	0.770	0.813
**12**	-0.251	0.089	0.609	0.664	0.725	0.793	0.870	0.615	0.648	0.683	0.770	0.814	0.860
**13**	-0.251	0.091	0.645	0.704	0.771	0.845	0.928	0.652	0.687	0.725	0.820	0.867	0.918
**14**	-0.251	0.093	0.683	0.747	0.818	0.899	0.989	0.690	0.728	0.769	0.872	0.923	0.978
**15**	-0.251	0.095	0.713	0.782	0.858	0.944	1.042	0.721	0.762	0.806	0.915	0.971	1.030
**16**	-0.251	0.096	0.738	0.810	0.891	0.983	1.086	0.747	0.789	0.836	0.952	1.011	1.073
**17**	-0.251	0.098	0.765	0.841	0.927	1.024	1.134	0.774	0.819	0.868	0.991	1.054	1.120
**18**	-0.251	0.100	0.781	0.860	0.949	1.050	1.165	0.790	0.836	0.888	1.016	1.081	1.151

Abbreviations: TBLH BMD, total body less head bone mineral density; SD, standard deviation.

[a] Approximately 2.28th percent

[b] Approximately 15.87th percent

[c] Approximately 50th percent

[d] Approximately 84.13th percent

[e] Approximately 97.72th percent

For peer review only

Supplemental Table 2. LMS parameters and reference centiles of TBLH BMD /g·cm^{-2} by age in girls

Age/yrs	L	S	Mean-2SD[a]	Mean-1SD[b]	Mean[c]	Mean+1SD[d]	Mean+2SD[e]	Percentiles					
								P3	P10	P25	P75	P90	P97
**3**	-0.480	0.061	0.342	0.363	0.385	0.409	0.436	0.345	0.357	0.370	0.401	0.417	0.433
**4**	-0.417	0.064	0.358	0.381	0.406	0.433	0.463	0.361	0.374	0.389	0.424	0.441	0.459
**5**	-0.293	0.070	0.391	0.419	0.449	0.482	0.518	0.395	0.411	0.428	0.471	0.492	0.513
**6**	-0.166	0.076	0.423	0.456	0.491	0.531	0.573	0.427	0.446	0.467	0.517	0.542	0.568
**7**	-0.035	0.082	0.455	0.493	0.535	0.581	0.630	0.459	0.482	0.506	0.565	0.594	0.624
**8**	0.092	0.087	0.482	0.527	0.575	0.626	0.682	0.487	0.514	0.542	0.609	0.642	0.676
**9**	0.205	0.091	0.508	0.558	0.612	0.670	0.733	0.514	0.544	0.576	0.651	0.687	0.725
**10**	0.294	0.094	0.537	0.592	0.652	0.715	0.783	0.543	0.576	0.611	0.694	0.734	0.775
**11**	0.358	0.095	0.573	0.633	0.696	0.764	0.836	0.580	0.615	0.653	0.742	0.784	0.827
**12**	0.388	0.092	0.613	0.676	0.743	0.813	0.887	0.620	0.658	0.697	0.790	0.834	0.878
**13**	0.375	0.088	0.651	0.714	0.781	0.852	0.927	0.658	0.696	0.736	0.829	0.873	0.918
**14**	0.314	0.084	0.680	0.742	0.807	0.877	0.950	0.687	0.724	0.763	0.854	0.897	0.941
**15**	0.208	0.080	0.699	0.759	0.823	0.891	0.963	0.706	0.741	0.779	0.868	0.911	0.954
**16**	0.071	0.078	0.714	0.772	0.835	0.902	0.975	0.720	0.755	0.792	0.880	0.922	0.966
**17**	-0.087	0.077	0.725	0.783	0.845	0.913	0.987	0.732	0.766	0.802	0.890	0.933	0.978
**18**	-0.257	0.076	0.732	0.788	0.850	0.918	0.993	0.738	0.771	0.807	0.895	0.938	0.984

Abbreviations: TBLH BMD, total body less head bone mineral density; SD, standard deviation.

[a] Approximately 2.28th percent

[b] Approximately 15.87th percent

[c] Approximately 50th percent

[d] Approximately 84.13th percent

[e] Approximately 97.72th percent

For peer review only

Supplemental Table 3. LMS parameters and reference centiles of TBLH BMD /g·cm^{-2} by height/cm in boys

Height/cm	L	S	Mean-2SD[a]	Mean-1SD[b]	Mean[c]	Mean+1SD[d]	Mean+2SD[e]	Percentiles					
								P3	P10	P25	P75	P90	P97
95	-1.272	0.045	0.343	0.358	0.374	0.392	0.412	0.345	0.354	0.363	0.386	0.397	0.409
100	-1.272	0.050	0.360	0.377	0.395	0.416	0.439	0.362	0.372	0.382	0.409	0.422	0.436
105	-1.272	0.054	0.380	0.399	0.420	0.444	0.472	0.382	0.393	0.405	0.436	0.452	0.468
110	-1.272	0.059	0.403	0.424	0.449	0.477	0.510	0.405	0.418	0.432	0.468	0.486	0.506
115	-1.272	0.064	0.429	0.454	0.483	0.516	0.554	0.432	0.447	0.463	0.504	0.526	0.549
120	-1.272	0.068	0.458	0.486	0.519	0.558	0.603	0.461	0.478	0.496	0.544	0.570	0.597
125	-1.272	0.072	0.486	0.518	0.555	0.599	0.651	0.490	0.509	0.530	0.584	0.613	0.645
130	-1.272	0.076	0.512	0.547	0.588	0.637	0.696	0.516	0.537	0.560	0.620	0.652	0.688
135	-1.272	0.079	0.537	0.575	0.620	0.674	0.739	0.541	0.564	0.589	0.655	0.691	0.731
140	-1.272	0.082	0.560	0.601	0.649	0.708	0.780	0.564	0.588	0.615	0.687	0.726	0.770
145	-1.272	0.085	0.581	0.624	0.677	0.740	0.819	0.585	0.611	0.640	0.718	0.761	0.809
150	-1.272	0.087	0.604	0.651	0.708	0.776	0.862	0.609	0.637	0.668	0.752	0.798	0.851
155	-1.272	0.090	0.634	0.684	0.745	0.820	0.915	0.639	0.669	0.703	0.794	0.845	0.902
160	-1.272	0.093	0.667	0.722	0.788	0.870	0.974	0.673	0.706	0.742	0.841	0.897	0.960
165	-1.272	0.095	0.701	0.760	0.831	0.919	1.032	0.707	0.742	0.781	0.888	0.948	1.017
170	-1.272	0.097	0.733	0.795	0.871	0.965	1.087	0.739	0.776	0.818	0.932	0.997	1.071
175	-1.272	0.098	0.762	0.827	0.908	1.008	1.138	0.769	0.808	0.852	0.973	1.041	1.121
180	-1.272	0.100	0.790	0.859	0.943	1.049	1.187	0.797	0.838	0.884	1.012	1.084	1.169
185	-1.272	0.101	0.818	0.890	0.979	1.091	1.236	0.826	0.868	0.917	1.051	1.128	1.217
190	-1.272	0.102	0.847	0.922	1.015	1.133	1.286	0.855	0.899	0.950	1.091	1.171	1.265
195	-1.272	0.103	0.876	0.955	1.052	1.175	1.337	0.884	0.931	0.984	1.132	1.216	1.315

Abbreviations: TBLH BMD, total body less head bone mineral density; SD, standard deviation.

[a] Approximately 2.28th percent

[b] Approximately 15.87th percent

[c] Approximately 50th percent

[d] Approximately 84.13th percent

[e] Approximately 97.72th percent

For peer review only

Supplemental Table 4. LMS parameters and reference centiles of-TBLH BMD /g·cm^{-2} by height/cm in girls

Height/cm	L	S	Mean-2SD[a]	Mean-1SD[b]	Mean[c]	Mean+1SD[d]	Mean+2SD[e]	Percentiles					
								P3	P10	P25	P75	P90	P97
95	-0.926	0.047	0.339	0.354	0.371	0.389	0.409	0.340	0.350	0.359	0.383	0.395	0.407
100	-1.168	0.050	0.355	0.371	0.390	0.411	0.434	0.356	0.366	0.377	0.404	0.417	0.431
105	-1.413	0.054	0.376	0.395	0.416	0.439	0.467	0.378	0.389	0.401	0.431	0.447	0.463
110	-1.665	0.058	0.398	0.419	0.443	0.471	0.504	0.401	0.413	0.427	0.461	0.480	0.500
115	-1.871	0.064	0.423	0.446	0.474	0.508	0.550	0.425	0.439	0.455	0.496	0.519	0.544
120	-1.980	0.072	0.449	0.476	0.509	0.550	0.603	0.452	0.468	0.486	0.536	0.563	0.596
125	-2.008	0.078	0.477	0.508	0.546	0.595	0.659	0.480	0.499	0.520	0.578	0.611	0.650
130	-1.950	0.083	0.501	0.536	0.579	0.634	0.708	0.505	0.526	0.549	0.614	0.653	0.698
135	-1.776	0.088	0.521	0.560	0.607	0.668	0.750	0.525	0.548	0.574	0.647	0.689	0.739
140	-1.499	0.093	0.545	0.589	0.642	0.710	0.798	0.550	0.576	0.605	0.686	0.732	0.786
145	-1.146	0.096	0.580	0.630	0.690	0.763	0.856	0.585	0.615	0.648	0.737	0.787	0.844
150	-0.764	0.095	0.626	0.682	0.747	0.824	0.916	0.632	0.665	0.702	0.798	0.848	0.905
155	-0.417	0.089	0.668	0.727	0.793	0.869	0.954	0.674	0.709	0.747	0.843	0.892	0.944
160	-0.159	0.083	0.698	0.756	0.821	0.892	0.970	0.704	0.739	0.777	0.868	0.913	0.961
165	-0.004	0.078	0.721	0.779	0.843	0.912	0.986	0.727	0.762	0.800	0.889	0.932	0.977
170	0.096	0.077	0.741	0.800	0.864	0.933	1.007	0.747	0.783	0.821	0.910	0.953	0.998
175	0.187	0.076	0.760	0.822	0.887	0.956	1.030	0.767	0.804	0.842	0.933	0.976	1.021
180	0.281	0.075	0.781	0.844	0.910	0.980	1.054	0.788	0.826	0.865	0.957	1.000	1.045

Abbreviations: TBLH BMD, total body less head bone mineral density; SD, standard deviation.

[a] Approximately 2.28th percent

[b] Approximately 15.87th percent

[c] Approximately 50th percent

[d] Approximately 84.13th percent

[e] Approximately 97.72th percent

For peer review only

Supplemental Table 5. LMS parameters and reference centiles of FMP by age in boys

Age/yrs	L	S	Mean-2SD[a]	Mean-1SD[b]	Mean[c]	Mean+1SD[d]	Mean+2SD[e]	Percentiles					
								P3	P10	P25	P75	P90	P97
**3**	-0.651	0.101	26.1	28.6	31.5	35.0	39.1	26.3	27.8	29.5	33.8	36.1	38.6
**4**	-0.585	0.120	24.4	27.2	30.6	34.7	39.7	24.7	26.4	28.3	33.3	36.0	39.0
**5**	-0.483	0.151	22.2	25.4	29.4	34.3	40.7	22.5	24.4	26.6	32.6	36.0	39.8
**6**	-0.388	0.179	20.5	24.1	28.7	34.5	42.1	20.9	23.0	25.5	32.5	36.5	41.1
**7**	-0.301	0.204	19.4	23.3	28.4	35.1	43.9	19.8	22.1	24.9	32.7	37.3	42.7
**8**	-0.230	0.224	18.6	22.9	28.5	35.9	45.7	19.1	21.6	24.6	33.2	38.4	44.4
**9**	-0.184	0.240	18.1	22.7	28.7	36.6	47.4	18.6	21.2	24.4	33.8	39.4	45.9
**10**	-0.163	0.252	17.6	22.3	28.6	37.0	48.4	18.1	20.9	24.2	34.0	39.9	46.9
**11**	-0.168	0.261	17.0	21.7	28.0	36.6	48.4	17.5	20.3	23.6	33.5	39.6	46.8
**12**	-0.198	0.267	16.3	20.8	27.0	35.5	47.4	16.7	19.4	22.6	32.4	38.4	45.8
**13**	-0.250	0.269	15.5	19.8	25.6	33.9	45.7	15.9	18.4	21.5	30.9	36.8	44.1
**14**	-0.318	0.270	14.8	18.8	24.3	32.2	43.9	15.2	17.5	20.4	29.3	35.1	42.3
**15**	-0.397	0.269	14.3	18.0	23.2	30.9	42.5	14.7	16.8	19.5	28.1	33.7	40.9
**16**	-0.481	0.266	14.0	17.5	22.5	29.9	41.6	14.3	16.4	18.9	27.1	32.6	39.9
**17**	-0.564	0.264	13.8	17.1	21.9	29.1	40.9	14.1	16.1	18.5	26.4	31.8	39.2
**18**	-0.645	0.261	13.6	16.7	21.3	28.3	40.3	13.9	15.7	18.0	25.7	31.1	38.5

Abbreviations: FMP, fat mass percentage (%); SD, standard deviation.

[a] Approximately 2.28th percent

[b] Approximately 15.87th percent

[c] Approximately 50th percent

[d] Approximately 84.13th percent

[e] Approximately 97.72th percent

For peer review only

Supplemental Table 6. LMS parameters and reference centiles of FMP by age in girls

Age/yrs	L	S	Mean-2SD[a]	Mean-1SD[b]	Mean[c]	Mean+1SD[d]	Mean+2SD[e]	Percentiles					
								P3	P10	P25	P75	P90	P97
**3**	0.285	0.106	27.8	31.1	34.7	38.5	42.6	28.2	30.2	32.2	37.2	39.6	42.1
**4**	0.285	0.118	26.6	30.1	34.0	38.1	42.7	27.0	29.1	31.3	36.7	39.4	42.1
**5**	0.285	0.140	24.5	28.4	32.8	37.6	42.9	24.9	27.3	29.8	36.0	39.0	42.2
**6**	0.285	0.158	22.8	27.1	31.8	37.2	43.1	23.3	25.8	28.6	35.4	38.8	42.4
**7**	0.285	0.173	21.6	26.1	31.1	36.9	43.3	22.1	24.8	27.7	34.9	38.6	42.5
**8**	0.285	0.183	20.9	25.4	30.7	36.7	43.4	21.4	24.1	27.1	34.6	38.5	42.6
**9**	0.285	0.187	20.5	25.1	30.5	36.6	43.5	21.0	23.8	26.8	34.5	38.4	42.6
**10**	0.285	0.188	20.4	25.0	30.4	36.5	43.4	20.9	23.7	26.7	34.4	38.3	42.5
**11**	0.285	0.185	20.6	25.1	30.4	36.4	43.2	21.1	23.8	26.8	34.4	38.2	42.3
**12**	0.285	0.179	21.0	25.5	30.6	36.5	43.1	21.5	24.1	27.1	34.5	38.3	42.3
**13**	0.285	0.173	21.6	26.0	31.1	36.8	43.2	22.1	24.7	27.6	34.8	38.5	42.4
**14**	0.285	0.165	22.4	26.8	31.7	37.3	43.5	22.9	25.5	28.3	35.4	38.9	42.7
**15**	0.285	0.157	23.4	27.6	32.5	37.9	43.9	23.8	26.4	29.1	36.0	39.5	43.1
**16**	0.285	0.150	24.2	28.4	33.1	38.4	44.2	24.7	27.2	29.9	36.6	39.9	43.5
**17**	0.285	0.143	24.9	29.0	33.5	38.6	44.2	25.3	27.8	30.4	36.9	40.1	43.5
**18**	0.285	0.137	25.3	29.3	33.7	38.5	43.9	25.8	28.1	30.7	36.9	40.0	43.2

Abbreviations: FMP, fat mass percentage (%); SD, standard deviation.

[a] Approximately 2.28th percent

[b] Approximately 15.87th percent

[c] Approximately 50th percent

[d] Approximately 84.13th percent

[e] Approximately 97.72th percent

For peer review only

Supplemental Table 7. LMS parameters and reference centiles of FMI by age in boys

Age/yrs	L	S	Mean-2SD[a]	Mean-1SD[b]	Mean[c]	Mean+1SD[d]	Mean+2SD[e]	Percentiles					
								P3	P10	P25	P75	P90	P97
**3**	-0.682	0.133	3.93	4.41	5.01	5.76	6.72	3.98	4.27	4.59	5.50	6.00	6.59
**4**	-0.667	0.167	3.57	4.12	4.83	5.77	7.05	3.63	3.95	4.33	5.43	6.09	6.88
**5**	-0.645	0.220	3.13	3.75	4.61	5.84	7.72	3.19	3.56	4.00	5.38	6.29	7.45
**6**	-0.625	0.268	2.85	3.54	4.53	6.08	8.71	2.92	3.32	3.82	5.49	6.68	8.31
**7**	-0.608	0.311	2.70	3.44	4.57	6.45	9.99	2.77	3.20	3.75	5.72	7.22	9.43
**8**	-0.593	0.347	2.63	3.43	4.70	6.92	11.46	2.71	3.17	3.77	6.04	7.86	10.70
**9**	-0.578	0.373	2.62	3.47	4.86	7.40	12.91	2.70	3.19	3.84	6.38	8.51	11.97
**10**	-0.565	0.392	2.61	3.51	5.00	7.79	14.07	2.70	3.21	3.91	6.66	9.03	12.97
**11**	-0.553	0.404	2.59	3.51	5.05	7.97	14.70	2.68	3.20	3.92	6.78	9.29	13.52
**12**	-0.541	0.410	2.55	3.46	5.02	7.97	14.80	2.63	3.16	3.88	6.77	9.30	13.60
**13**	-0.530	0.412	2.49	3.40	4.93	7.84	14.52	2.58	3.09	3.80	6.65	9.14	13.34
**14**	-0.520	0.410	2.44	3.32	4.82	7.65	14.04	2.52	3.03	3.72	6.50	8.91	12.93
**15**	-0.510	0.407	2.40	3.27	4.73	7.47	13.53	2.48	2.98	3.66	6.36	8.68	12.48
**16**	-0.501	0.402	2.37	3.23	4.66	7.31	13.05	2.45	2.95	3.61	6.24	8.47	12.07
**17**	-0.492	0.398	2.35	3.20	4.60	7.16	12.61	2.43	2.92	3.58	6.13	8.27	11.69
**18**	-0.483	0.393	2.33	3.16	4.54	7.01	12.18	2.41	2.89	3.53	6.02	8.08	11.32

Abbreviations: FMI, fat mass index (Fat mass (kg)/height(m)^2) ; SD, standard deviation.

[a] Approximately 2.28th percent

[b] Approximately 15.87th percent

[c] Approximately 50th percent

[d] Approximately 84.13th percent

[e] Approximately 97.72th percent

For peer review only

Supplemental Table 8. LMS parameters for the reference centiles of FMI by age in girls

Age/yrs	L	S	Mean-2SD[a]	Mean-1SD[b]	Mean[c]	Mean+1SD[d]	Mean+2SD[e]	Percentiles					
								P3	P10	P25	P75	P90	P97
**3**	-0.731	0.146	4.11	4.66	5.36	6.25	7.45	4.17	4.49	4.87	5.94	6.55	7.28
**4**	-0.697	0.167	3.87	4.46	5.23	6.25	7.65	3.93	4.28	4.69	5.88	6.60	7.46
**5**	-0.635	0.206	3.47	4.13	5.01	6.25	8.08	3.54	3.92	4.38	5.79	6.69	7.82
**6**	-0.577	0.239	3.19	3.89	4.86	6.29	8.51	3.26	3.67	4.17	5.76	6.81	8.19
**7**	-0.523	0.266	3.00	3.74	4.79	6.38	8.94	3.07	3.50	4.04	5.79	6.98	8.57
**8**	-0.472	0.287	2.90	3.69	4.82	6.56	9.41	2.98	3.44	4.01	5.91	7.22	8.98
**9**	-0.423	0.300	2.90	3.73	4.95	6.82	9.89	2.98	3.47	4.08	6.12	7.53	9.44
**10**	-0.376	0.308	2.96	3.84	5.14	7.12	10.34	3.04	3.56	4.21	6.38	7.87	9.87
**11**	-0.331	0.309	3.06	4.00	5.38	7.45	10.74	3.16	3.70	4.39	6.67	8.22	10.26
**12**	-0.288	0.306	3.21	4.21	5.65	7.79	11.09	3.32	3.90	4.62	6.99	8.57	10.61
**13**	-0.246	0.299	3.40	4.46	5.95	8.11	11.35	3.51	4.12	4.88	7.31	8.89	10.89
**14**	-0.205	0.288	3.62	4.72	6.25	8.41	11.54	3.73	4.37	5.16	7.62	9.18	11.10
**15**	-0.165	0.276	3.85	4.99	6.53	8.66	11.64	3.97	4.63	5.44	7.89	9.40	11.23
**16**	-0.126	0.263	4.06	5.22	6.76	8.83	11.64	4.18	4.86	5.67	8.08	9.53	11.26
**17**	-0.088	0.250	4.24	5.40	6.91	8.90	11.52	4.36	5.04	5.84	8.19	9.56	11.17
**18**	-0.051	0.237	4.38	5.53	7.01	8.90	11.33	4.50	5.18	5.97	8.23	9.52	11.01

Abbreviations: FMI, fat mass index (Fat mass (kg)/height(m)^2) ; SD, standard deviation.

[a] Approximately 2.28th percent

[b] Approximately 15.87th percent

[c] Approximately 50th percent

[d] Approximately 84.13th percent

[e] Approximately 97.72th percent

For peer review only

Supplemental Table 9. LMS parameters and reference centiles of FFMI by age in boys

Age/yrs	L	S	Mean-2SD[a]	Mean-1SD[b]	Mean[c]	Mean+1SD[d]	Mean+2SD[e]	Percentiles					
								P3	P10	P25	P75	P90	P97
**3**	-0.656	0.058	9.58	10.12	10.71	11.36	12.07	9.64	9.96	10.30	11.14	11.55	11.99
**4**	-0.656	0.062	9.66	10.25	10.90	11.61	12.41	9.73	10.08	10.45	11.37	11.83	12.31
**5**	-0.656	0.070	9.80	10.46	11.20	12.04	12.98	9.87	10.27	10.69	11.76	12.29	12.87
**6**	-0.656	0.078	9.86	10.60	11.43	12.38	13.47	9.94	10.38	10.86	12.06	12.68	13.34
**7**	-0.656	0.085	9.92	10.73	11.66	12.73	13.98	10.01	10.49	11.02	12.37	13.06	13.82
**8**	-0.656	0.093	10.08	10.98	12.01	13.22	14.64	10.18	10.71	11.30	12.81	13.60	14.46
**9**	-0.656	0.100	10.22	11.20	12.34	13.70	15.31	10.33	10.91	11.55	13.23	14.12	15.10
**10**	-0.656	0.108	10.35	11.41	12.66	14.15	15.96	10.47	11.09	11.79	13.63	14.62	15.73
**11**	-0.656	0.114	10.61	11.75	13.12	14.76	16.78	10.74	11.41	12.17	14.19	15.29	16.52
**12**	-0.656	0.118	11.06	12.29	13.78	15.59	17.82	11.19	11.92	12.75	14.96	16.17	17.53
**13**	-0.656	0.121	11.66	12.99	14.60	16.56	19.00	11.81	12.59	13.48	15.88	17.19	18.68
**14**	-0.656	0.122	12.30	13.72	15.42	17.51	20.11	12.46	13.29	14.24	16.78	18.18	19.77
**15**	-0.656	0.121	12.78	14.24	16.00	18.16	20.84	12.94	13.80	14.78	17.41	18.85	20.49
**16**	-0.656	0.120	13.02	14.50	16.27	18.43	21.12	13.19	14.06	15.04	17.68	19.13	20.77
**17**	-0.656	0.118	13.19	14.66	16.43	18.57	21.22	13.35	14.22	15.20	17.83	19.26	20.88
**18**	-0.656	0.116	13.36	14.82	16.56	18.68	21.29	13.52	14.38	15.35	17.95	19.36	20.95

Abbreviations: FFMI, free fat mass index (Free fat mass (kg)/height(m)^2) ; SD, standard deviation.

[a] Approximately 2.28th percent

[b] Approximately 15.87th percent

[c] Approximately 50th percent

[d] Approximately 84.13th percent

[e] Approximately 97.72th percent

For peer review only

Supplemental Table 10. LMS parameters and reference centiles of FFMI by age in girls

Age/yrs	L	S	Mean-2SD[a]	Mean-1SD[b]	Mean[c]	Mean+1SD[d]	Mean+2SD[e]	Percentiles					
								P3	P10	P25	P75	P90	P97
**3**	-0.485	0.060	8.97	9.50	10.09	10.72	11.42	9.03	9.35	9.69	10.51	10.91	11.33
**4**	-0.484	0.064	9.00	9.57	10.19	10.87	11.62	9.06	9.40	9.76	10.64	11.07	11.53
**5**	-0.482	0.071	9.06	9.69	10.39	11.16	12.03	9.13	9.50	9.91	10.90	11.40	11.92
**6**	-0.480	0.078	9.11	9.81	10.59	11.46	12.44	9.19	9.60	10.05	11.16	11.72	12.32
**7**	-0.479	0.084	9.19	9.96	10.81	11.79	12.90	9.28	9.73	10.22	11.46	12.09	12.76
**8**	-0.477	0.091	9.34	10.17	11.11	12.19	13.43	9.43	9.92	10.46	11.82	12.52	13.28
**9**	-0.475	0.096	9.55	10.46	11.49	12.68	14.06	9.66	10.19	10.78	12.27	13.04	13.88
**10**	-0.474	0.101	9.84	10.81	11.93	13.22	14.74	9.94	10.52	11.15	12.78	13.63	14.55
**11**	-0.472	0.104	10.17	11.21	12.40	13.80	15.44	10.29	10.90	11.58	13.32	14.24	15.23
**12**	-0.470	0.106	10.51	11.60	12.87	14.34	16.08	10.63	11.28	11.99	13.84	14.81	15.86
**13**	-0.469	0.106	10.81	11.94	13.24	14.77	16.57	10.94	11.60	12.34	14.25	15.25	16.34
**14**	-0.467	0.106	11.05	12.19	13.51	15.06	16.88	11.17	11.85	12.60	14.53	15.54	16.65
**15**	-0.465	0.104	11.21	12.35	13.67	15.21	17.01	11.34	12.01	12.76	14.68	15.69	16.78
**16**	-0.463	0.102	11.31	12.44	13.74	15.25	17.01	11.44	12.11	12.84	14.73	15.72	16.79
**17**	-0.462	0.099	11.39	12.50	13.77	15.24	16.94	11.52	12.17	12.89	14.73	15.69	16.73
**18**	-0.460	0.096	11.48	12.56	13.80	15.22	16.87	11.60	12.24	12.94	14.74	15.66	16.66

Abbreviations: FFMI, free fat mass index (Free fat mass (kg)/height(m)^2) ; SD, standard deviation.

[a] Approximately 2.28th percent

[b] Approximately 15.87th percent

[c] Approximately 50th percent

[d] Approximately 84.13th percent

[e] Approximately 97.72th percent

For peer review only

STROBE Statement—checklist of items that should be included in reports of observational studies

	Item No	Recommendation
**Title and abstract** Page 2	1	(*a*) Indicate the study's design with a commonly used term in the title or the abstract
		(*b*) Provide in the abstract an informative and balanced summary of what was done and what was found
**Introduction**		
Background/rationale Page 4	2	Explain the scientific background and rationale for the investigation being reported
Objectives Page 6	3	State specific objectives, including any prespecified hypotheses
**Methods**		
Study design Page 6	4	Present key elements of study design early in the paper
Setting Page 6	5	Describe the setting, locations, and relevant dates, including periods of recruitment, exposure, follow-up, and data collection
Participants Page 7	6	(*a*) *Cohort study*—Give the eligibility criteria, and the sources and methods of selection of participants. Describe methods of follow-up<br>*Case-control study*—Give the eligibility criteria, and the sources and methods of case ascertainment and control selection. Give the rationale for the choice of cases and controls<br>*Cross-sectional study*—Give the eligibility criteria, and the sources and methods of selection of participants
		(*b*) *Cohort study*—For matched studies, give matching criteria and number of exposed and unexposed<br>*Case-control study*—For matched studies, give matching criteria and the number of controls per case
Variables Page 7	7	Clearly define all outcomes, exposures, predictors, potential confounders, and effect modifiers. Give diagnostic criteria, if applicable
Data sources/ measurement Page 8	8*	For each variable of interest, give sources of data and details of methods of assessment (measurement). Describe comparability of assessment methods if there is more than one group
Bias Page 8	9	Describe any efforts to address potential sources of bias
Study size Page 9	10	Explain how the study size was arrived at
Quantitative variables Page 9	11	Explain how quantitative variables were handled in the analyses. If applicable, describe which groupings were chosen and why
Statistical methods Page 9	12	(*a*) Describe all statistical methods, including those used to control for confounding
		(*b*) Describe any methods used to examine subgroups and interactions
		(*c*) Explain how missing data were addressed
		(*d*) *Cohort study*—If applicable, explain how loss to follow-up was addressed<br>*Case-control study*—If applicable, explain how matching of cases and controls was addressed<br>*Cross-sectional study*—If applicable, describe analytical methods taking account of sampling strategy

For peer review only

		(*e*) Describe any sensitivity analyses
**Results**		
Participants<br>Page 9	13*	(a) Report numbers of individuals at each stage of study—eg numbers potentially eligible, examined for eligibility, confirmed eligible, included in the study, completing follow-up, and analysed
		(b) Give reasons for non-participation at each stage
		(c) Consider use of a flow diagram
Descriptive data<br>Page 10	14*	(a) Give characteristics of study participants (eg demographic, clinical, social) and information on exposures and potential confounders
		(b) Indicate number of participants with missing data for each variable of interest
		(c) *Cohort study*—Summarise follow-up time (eg, average and total amount)
Outcome data<br>Page 10	15*	*Cohort study*—Report numbers of outcome events or summary measures over time
		Case-control study—Report numbers in each exposure category, or summary measures of exposure
		Cross-sectional study—Report numbers of outcome events or summary measures
Main results<br>Page 10	16	(*a*) Give unadjusted estimates and, if applicable, confounder-adjusted estimates and their precision (eg, 95% confidence interval). Make clear which confounders were adjusted for and why they were included
		(*b*) Report category boundaries when continuous variables were categorized
		(*c*) If relevant, consider translating estimates of relative risk into absolute risk for a meaningful time period
Other analyses<br>Page 10	17	Report other analyses done—eg analyses of subgroups and interactions, and sensitivity analyses
**Discussion**		
Key results<br>Page 11	18	Summarise key results with reference to study objectives
Limitations<br>Page 13	19	Discuss limitations of the study, taking into account sources of potential bias or imprecision. Discuss both direction and magnitude of any potential bias
Interpretation<br>Page 13	20	Give a cautious overall interpretation of results considering objectives, limitations, multiplicity of analyses, results from similar studies, and other relevant evidence
Generalisability<br>Page 13	21	Discuss the generalisability (external validity) of the study results
**Other information**		
Funding<br>Page 15	22	Give the source of funding and the role of the funders for the present study and, if applicable, for the original study on which the present article is based

*Give information separately for cases and controls in case-control studies and, if applicable, for exposed and unexposed groups in cohort and cross-sectional studies.

Note: An Explanation and Elaboration article discusses each checklist item and gives methodological background and published examples of transparent reporting. The STROBE checklist is best used in conjunction with this article (freely available on the Web sites of PLoS Medicine at http://www.plosmedicine.org/, Annals of Internal Medicine at http://www.annals.org/, and Epidemiology at http://www.epidem.com/). Information on the STROBE Initiative is available at www.strobe-statement.org.

For peer review only

For peer review only - http://bmjopen.bmj.com/site/about/guidelines.xhtml
